# Uncovering a Macrophage Transcriptional Program by Integrating Evidence from Motif Scanning and Expression Dynamics

**DOI:** 10.1371/journal.pcbi.1000021

**Published:** 2008-03-21

**Authors:** Stephen A. Ramsey, Sandy L. Klemm, Daniel E. Zak, Kathleen A. Kennedy, Vesteinn Thorsson, Bin Li, Mark Gilchrist, Elizabeth S. Gold, Carrie D. Johnson, Vladimir Litvak, Garnet Navarro, Jared C. Roach, Carrie M. Rosenberger, Alistair G. Rust, Natalya Yudkovsky, Alan Aderem, Ilya Shmulevich

**Affiliations:** Institute for Systems Biology, Seattle, Washington, United States of America; Massachusetts Institute of Technology and Harvard University, United States of America

## Abstract

Macrophages are versatile immune cells that can detect a variety of pathogen-associated molecular patterns through their Toll-like receptors (TLRs). In response to microbial challenge, the TLR-stimulated macrophage undergoes an activation program controlled by a dynamically inducible transcriptional regulatory network. Mapping a complex mammalian transcriptional network poses significant challenges and requires the integration of multiple experimental data types. In this work, we inferred a transcriptional network underlying TLR-stimulated murine macrophage activation. Microarray-based expression profiling and transcription factor binding site motif scanning were used to infer a network of associations between transcription factor genes and clusters of co-expressed target genes. The time-lagged correlation was used to analyze temporal expression data in order to identify potential causal influences in the network. A novel statistical test was developed to assess the significance of the time-lagged correlation. Several associations in the resulting inferred network were validated using targeted ChIP-on-chip experiments. The network incorporates known regulators and gives insight into the transcriptional control of macrophage activation. Our analysis identified a novel regulator (TGIF1) that may have a role in macrophage activation.

## Author Summary

Macrophages play a vital role in host defense against infection by recognizing pathogens through pattern recognition receptors, such as the Toll-like receptors (TLRs), and mounting an immune response. Stimulation of TLRs initiates a complex transcriptional program in which induced transcription factor genes dynamically regulate downstream genes. Microarray-based transcriptional profiling has proved useful for mapping such transcriptional programs in simpler model organisms; however, mammalian systems present difficulties such as post-translational regulation of transcription factors, combinatorial gene regulation, and a paucity of available gene-knockout expression data. Additional evidence sources, such as DNA sequence-based identification of transcription factor binding sites, are needed. In this work, we computationally inferred a transcriptional network for TLR-stimulated murine macrophages. Our approach combined sequence scanning with time-course expression data in a probabilistic framework. Expression data were analyzed using the time-lagged correlation. A novel, unbiased method was developed to assess the significance of the time-lagged correlation. The inferred network of associations between transcription factor genes and co-expressed gene clusters was validated with targeted ChIP-on-chip experiments, and yielded insights into the macrophage activation program, including a potential novel regulator. Our general approach could be used to analyze other complex mammalian systems for which time-course expression data are available.

## Introduction

Dynamic cellular processes, such as the response to a signaling event, are governed by complex transcriptional regulatory networks. These networks typically involve a large number of transcription factors (TFs) that are activated in different combinations in order to produce a particular cellular response. The macrophage, a vital cell type of the mammalian immune system, marshals a variety of phenotypic responses to pathogenic challenge, such as secretion of pro-inflammatory mediators, phagocytosis and antigen presentation, stimulation of mucus production, and adherence. In the innate immune system, the first line of defense against infection, the macrophage's Toll-like receptors (TLRs) play a crucial role by recognizing distinct pathogen-associated molecular patterns (PAMPs), such as flagellin, lipopeptides, or double-stranded RNA [1],[2]. TLR signals are first channeled through adapter molecules (e.g., TICAM1/TRIF [3],[4] and MyD88 [5]) and then through parallel cross-talking signal pathways. These activated pathways initiate a transcriptional program in which over 1,000 genes [6] and hundreds of TF genes [7] can be differentially expressed, and which is tailored to the type of infection [8],[9]. The transcriptional network underlying macrophage activation can exhibit many distinct steady-states which are associated with tissue- and infection-specific macrophage functions [10]. The transcriptional response is also dynamic and characterized by temporal waves of gene activation [6],[7],[9], each enriched for distinct sets of gene functions [7],[9] and likely to be controlled by different combinations of transcriptional regulators [6],[7]. Long-term, elucidating the transcriptional network underlying TLR-stimulated macrophage activation, and identifying key regulators and their functions, would greatly enhance our understanding of the innate immune response to infection and potentially yield new ideas for vaccine development.

Computational analysis of high-throughput experimental data is proving increasingly useful in the inference of transcriptional regulatory interaction networks [11]–[15] and in the identification and prioritization of potential regulators for targeted experimental validation [6],[7]. Time-course microarray expression measurements have been used to infer dynamic transcriptional networks in yeast [14],[15] and static “influence” networks in mammalian cell lines [11]. In the context of primary macrophages, expression-based computational reconstruction of the transcriptional control logic underlying the activation program is not straightforward and progress is difficult to measure, for several reasons. First, transcriptional control within mammalian networks in general [16], and for key TLR-responsive genes in particular [7], is combinatorial. Second, many induced TFs are subject to post-translational activation [17] and dynamic control of nuclear localization [6]. Third, targeted genetic perturbations are presently infeasible to perform on a large scale in a mammalian animal model, and expression knockdown (RNAi) is difficult in macrophages due to the tendency of the vector to stimulate TLRs. Finally, the few transcriptional regulatory interactions that have been validated through targeted experiments in TLR-stimulated primary macrophages are not available in a single “gold standard” dataset. Therefore, in the context of transcriptional regulation in the mammalian macrophage, with presently accessible expression data sets, large-scale computational inference is primarily useful for statistically identifying potential regulatory interactions, rather than as an inference tool for predicting the transcriptional control logic for specific target genes.

For the reasons described above, in order to computationally infer transcriptional regulatory interactions in a mammalian system, it is necessary to include additional sources of evidence (beyond expression data) to constrain or inform the transcriptional network model selection. Computational scanning of the promoter sequences of clusters of co-expressed genes for known transcription factor binding site (TFBS) motifs has proved particularly valuable when combined with global expression data [6],[18],[19]. Recently, Nilsson *et al.* [7] used a combination of expression clustering and promoter sequence scanning for TFBS motifs to construct an initial transcriptional network of the macrophage stimulated with the TLR4 stimulus lipopolysaccharide (LPS). Their work identified two novel regulators, but the clustering was based on an expression dataset with a single stimulus, limited biological replicates, and few time points. Moreover, TFBS motif scanning of co-expressed clusters, without utilizing expression dynamics, provides only a limited and static picture of the underlying transcriptional network. Many TFBS motifs are often recognized by multiple TFs, making difficult the unambiguous identification of the regulating TF from TFBS enrichment alone. Furthermore, because of the tendency of TFBS motifs to co-occur [20], it is difficult to determine from among a set of co-occurring motifs which associated TF is the most relevant to the condition-specific regulation of the target cluster. In the TLR-stimulated macrophage, core transcription factors already expressed in the cell (e.g., NFkB, AP1, and CREB) are rapidly activated and initiate transcriptional regulation of “second wave” TF genes [6]. Such transcriptionally regulated TF genes are key candidates for an integrated analysis combining TF-specific dynamic expression data and sequence-based motif scanning data.

This work is concerned with using computational data integration to identify a set of core differentially expressed transcriptional regulators in the TLR-stimulated macrophage and, in the form of statistical associations, the clusters of co-expressed genes that they may regulate. The clusters are differentiated based on temporal and stimulus-specific activation, and in this sense, the inferred associations constitute a preliminary dynamic transcriptional network for the TLR-stimulated macrophage. To achieve this, we used a novel computational approach incorporating TFBS motif scanning and statistical inference based on time-course expression data across a diverse array of stimuli. Our approach involved four steps. (i) A set of genes was identified that were differentially expressed by wild-type macrophages under at least one TLR stimulation experiment. (ii) These genes were clustered based on their expression profiles across a wide range of conditions and strains, grouping genes based on the similarity of the timing and stimulus-dependence of their induction. Gene Ontology annotations were used to identify functional categories enriched within the gene clusters. (iii) Promoter sequences upstream of the genes within each cluster were scanned for a library of TFBS motifs, each recognized by at least one differentially expressed TF, to identify possible associations between TFs and gene clusters. (iv) Across eleven different time-course studies, dynamic expression profiles of TF genes and target genes were compared in order to identify possible causal influences between differentially expressed TF genes and clusters.

Several techniques have been developed specifically for model inference from time-course expression data, notably dynamic Bayesian networks (DBN) [21] and ODE-based model selection [12]. However, the parametric complexity of these model classes makes it difficult to apply them to infer a network underlying a specific cellular perturbation (e.g., TLR activation in the macrophage) with a limited expression dataset. Here, potential transcriptional regulatory influence is inferred from time-course expression data using the time-lagged correlation (TLC) statistic, which has been used to infer biochemical interaction networks [22] as well as transcriptional networks [23]–[29]. The TLC has the advantage that it accounts for the time delay between differential expression of an induced TF and differential expression of a target gene. In contrast to standard correlation-based methods that identify co-expressed genes, the TLC method uses temporal ordering of expression to determine whether the time lag between two correlated genes is consistent with a causal interaction. We developed a novel method to identify the optimal time lag for each gene pair, and used a prior probability distribution of transcriptional time delays to score possible interactions.

By combining the promoter scanning-based evidence with the evidence obtained by the time-lagged correlation analysis of the expression data, we were able to identify a network of statistically significant associations between 36 TF genes and 27 co-expressed clusters. Overall, 63% of differentially expressed genes are included in the network. The network provided insights into the temporal organization of the transcriptional response and into combinations of TFs that may act as key regulators of macrophage activation. Finally, the analysis identified a potential transcriptional regulator, TGIF1 (*Tgif1*), which was not previously known to play a role in macrophage activation. As a targeted experimental validation of the inferred network, two transcriptional regulators, p50 (a component of NFkB) and IRF1, were assayed for binding to *cis*-regulatory elements in LPS-stimulated macrophages using ChIP-on-chip, and were confirmed to bind the promoters of genes in four out of five predicted target clusters at significantly higher proportions than expected for a random set of TLR-responsive genes.

## Results

### Gene selection and clustering

To probe a diverse set of transcriptional responses of Toll-like receptor (TLR)-stimulated macrophages, primary bone marrow-derived macrophages (BMMs) from five mouse strains (wild-type and four mutant strains; see Table S1) were stimulated with six purified TLR agonists representing various pathogen-associated molecular patterns (PAMPs). The TLR agonists include bacterial-associated (lipopolysaccharide, Pam_2_CSK_4_, Pam_3_CSK_4_, CpG), viral-associated (poly I:C), and anti-viral (R848) stimuli, and are listed in Table S2. The mutant strains, which were included to increase the diversity of the TLR-stimulated gene expression dataset and to increase the number of time-course measurements used, consisted of null mutations of the two key adapter molecules for the TLR signaling pathway (TRIF [3] and MyD88 [5]) and two TFs predicted to be involved in TLR activation (ATF3 [30] and CREM [31]). Genome-wide expression measurements of 45,037 probesets, representing 23,259 annotated genes, were made for time courses of up to 48 hours post-stimulation, using oligonucleotide microarrays (see Materials and Methods). In all, expression measurements were made for 95 distinct combinations of strain, stimulus, and elapsed time (hereafter, “experiments”; see Table S3). Using a spline-based multivariate regression method specifically adapted for significance testing of temporal expression datasets [32], annotated probesets were analyzed for differential expression across seven TLR-stimulated wild-type expression time-courses. After filtering for minimum absolute expression intensity and differential expression under at least one TLR-stimulation experiment (see Materials and Methods), 1,960 probesets were identified as significantly differentially expressed, with each probeset mapped to a unique gene (see Table S4). Of these, 44% were found to be upregulated in LPS-stimulated wild-type macrophages. Additionally, a set of 80 TF genes (for which corresponding position-weight matrices are available in the TRANSFAC database [33]) were found to be differentially expressed in the TLR-stimulated wild-type macrophage (Table S5). Those of TF families with established relevance in macrophage activation included two NFkB [34] component genes (*Rel*, *Nfkb1*), three AP1 [35] components (*Jun*, *Junb*, *Fos*), two ATF family genes [6] (*Atf1, Atf3*), six IRF family TF genes (*Irf1/2/3/5/7/9*) [17], and four STAT family TF genes [36] (*Stat1/3/4/5a*). The 80 TF genes were taken to constitute the set of potential regulators in the TLR-stimulated macrophage network.

**Figure 1:**
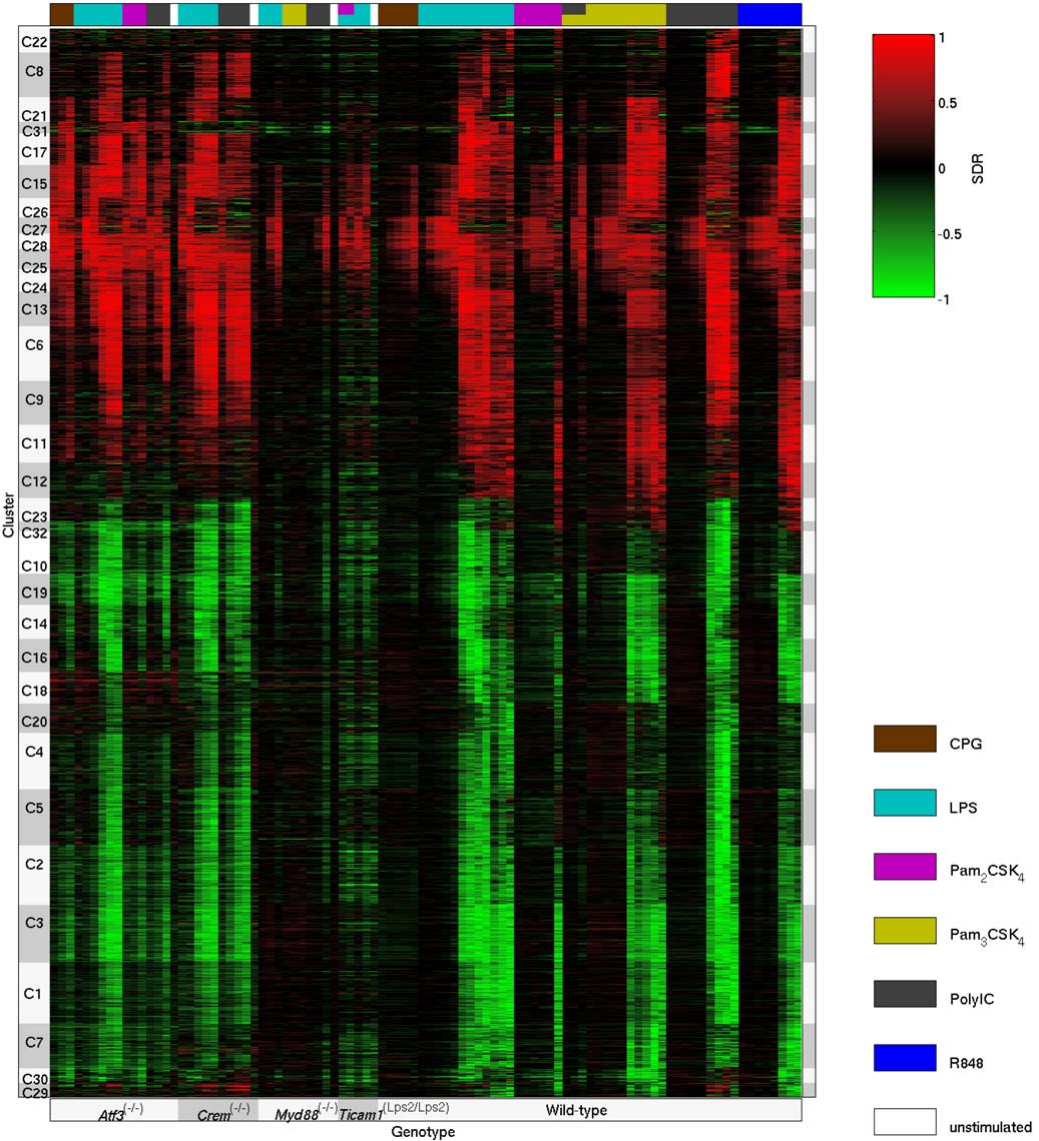
Global heat-map of differential gene expression in TLR-stimulated murine macrophages, organized by clusters of co-expressed genes. Each row is one of the 1,960 genes that are differentially expressed in macrophages under TLR stimulation, and each column is a replicate-combined microarray experiment. Red/green coloring indicates the differential expression level (SDR-normalized, see Equation 1). Red indicates upregulation relative to wild-type unstimulated macrophages. Green indicates downregulation relative to wild-type unstimulated macrophages. Genotypes are indicated along the bottom edge. Clusters are indicated along the left edge. Stimuli are indicated along the top edge, with the color scheme given in the lower right corner. Clusters have been ordered based on pairwise similarity, as described in Materials and Methods, Expression Clustering.

Clustering was used to identify cohorts of genes that were co-expressed across the diverse set of TLR-stimulation experiments, based on the assumption that genes within a cluster are likely to share common *cis*-regulatory elements such as TF binding sites [18]. In order to focus on TF control of the *timing* and *stimulus specificity* of gene expression, genes were clustered based on the normalized profile of expression, rather than based on the fold-change. Expression measurements were transformed based on a single universal reference experiment (wild-type unstimulated macrophages) so that the transformed measurements would all lie between −1 and 1, with zero indicating the intensity in the reference experiment. This technique, which we call the signed difference ratio (SDR), has previously proved useful in clustering genes based on temporal expression in a mammalian system [37]. Each log_2_ intensity measurement *y_pj_* for probeset *p* and non-reference experiment *j*, was transformed to an SDR value *x_pj_* by
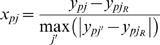
(1)where *j_R_* is the index of the global reference experiment (*j*′ has the same range of values as *j*). By construction, −1≤*x_pj_*≤1 for all *p* and *j*. A positive SDR value indicates higher expression than in the reference experiment, and a negative value indicates lower expression. The SDR-transformed log_2_ intensities of all 1,960 target genes across all 94 non-reference experiments were clustered using an unsupervised algorithm (*K*-means with Euclidean distance), with the number of clusters chosen using the Bayesian information criterion (BIC) [38] (see Materials and Methods, and Figure S1). The target genes were partitioned into 32 clusters (see Table S4, column 5). The differences in temporal and stimulus-specific expression between the clusters are clearly visible in a heat-map representation of the SDR-transformed expression data (hereafter, “expression data”) (Figure 1; see also Figure S2 for the cluster-median expression heat-map).

**Figure 2:**
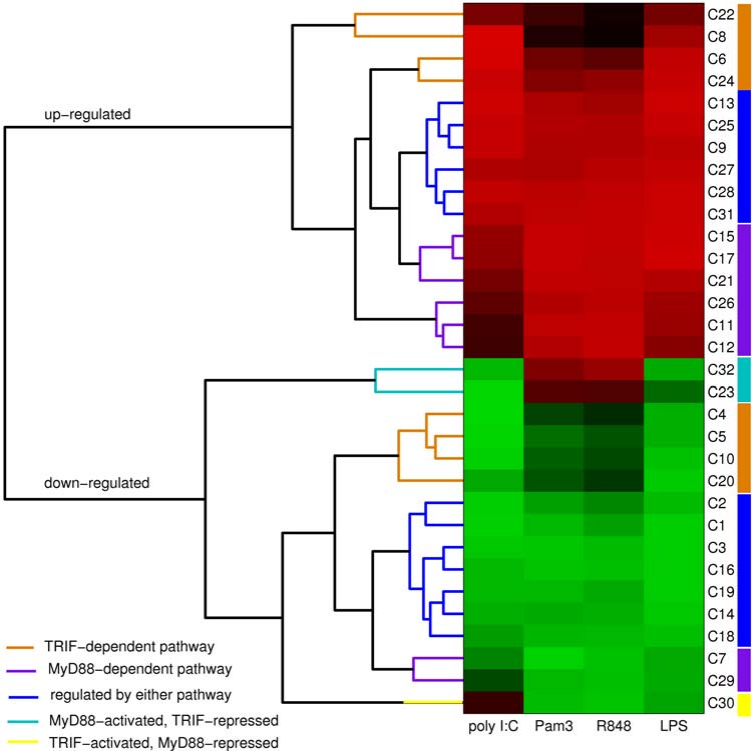
Hierarchical organization of differentially expressed gene clusters from TLR-stimulated macrophages reveals pathway-specific transcriptional responses. The color of a rectangle in the heat-map shows the cluster-median differential expression (relative to wild-type unstimulated macrophages) under stimulation with the TLR agonist indicated by the column label (bottom of figure), for the cluster indicated by the row label (right-hand side). The column label Pam3 denotes the TLR agonist Pam_3_CSK_4_. The differential gene expression level is computed using the signed difference ratio (SDR, see Equation 1). Clusters (rows) have been ordered for display based on similarity of overall transcriptional response to the four indicated TLR agonists (see Materials and Methods, Expression Clustering). In the heat-map, green indicates downregulation, and red indicates upregulation. Colored subtrees of the dendrogram indicate specific inferences that can be made about the likely signaling pathway (MyD88-dependent, TRIF-dependent, or a combination) on which the transcriptional regulation of the cluster depends. The legend in the lower-left corner explains the color scheme for denoting the inferred signaling pathway-dependence of the clusters. Clusters without a color bar on the right appear to respond through either signaling pathway. The regulation of clusters C7, C11, C12, C15, C17, C21, C26, and C29 appears to be primarily MyD88-dependent; regulation of clusters C4, C5, C6, C8, C10, C20, C22, and C24 appears to be primarily TRIF-dependent; and clusters C23, C30, and C32 appear to be regulated oppositely by the two signaling pathways. This plot shows only the extremal differential response to TLR agonists; the clusters also differ in temporal expression (see Figure S3, Figure S4, Figure S5, and Figure S6).

The clusters (Table S6), which ranged in size from 18 to 113 genes, exhibit a significant diversity of timing and TLR-specificity of response. The wild-type LPS time-course was used to characterize the time scale for each cluster to respond transcriptionally (see Materials and Methods, and Table S6 columns 3–4). A small number of clusters reach peak induction within the first hour (C28, C27, C25, C26), but the majority of clusters (representing 55% of genes) respond between 2–4 hours. The temporal profiles of the clusters in wild-type BMMs under stimulation by LPS, Pam_3_CSK_4_, poly I:C, and R848 are shown in Figure S3, Figure S4, Figure S5, and Figure S6, respectively. The clusters exhibit distinct temporal profiles of transcriptional activation and repression that vary in the time of initial response and the duration of differential expression. Across all four stimuli, cluster C28 is induced first (and has sustained induction), followed by cluster C27 (which undergoes transient (2–3 h) upregulation), and then by induction of C25 and C26. Induction of C27 and C28 is delayed approximately 1 h under poly I:C stimulation, while C26 fails to fully induce under poly I:C. A comparison of the responses of clusters under 8 hours post-stimulation (LPS, Pam_3_CSK_4_, poly I:C, and R848) enabled the segregation of these clusters based on the signal transduction pathway through which they are likely primarily regulated (Figure 2). Groups include those primarily induced (C11, C12, C15, C17, C21, C26) and downregulated (C7, C29) by the MyD88-dependent pathway, and those primarily induced (C6, C8, C22, C24) and downregulated (C4, C5, C10, C20) by the TRIF-dependent pathway. Although “core early response” clusters C27 and C28 appear to be inducible through either signaling pathway, a comparison of the wild-type LPS vs. poly I:C response and of the wild-type vs. *Ticam1* ^(Lps2/Lps2)^ and *Myd88* ^(−/−)^ responses under LPS (see Table S7) together indicate that the MyD88-dependent pathway is responsible for the early response (within the first hour), and the TRIF-dependent pathway is responsible for sustaining the induction of these clusters. Early induced TF genes (*Egr1/2/3, Junb, Rel, Irf1*) also appear to be inducible through either pathway, from analysis of the LPS response in *Ticam1* ^(Lps2/Lps2)^ and *Myd88* ^(−/−)^ macrophages.

**Figure 3:**
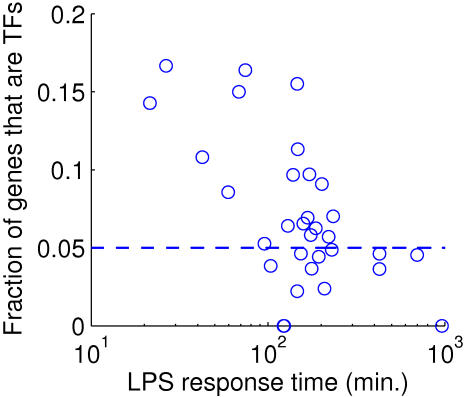
Early induced gene clusters are enriched for transcription factors. Each circular data point indicates a cluster. The horizontal axis is the estimated time scale for the differential expression level of the genes within the cluster to reach 25% of the maximum absolute differential expression (the “response time”). The response time was computed under LPS stimulation of wild-type macrophages (see Materials and Methods, Expression Clustering). The horizontal dashed line indicates the average fraction of genes that are known transcription factors, among all annotated genes in the mouse genome (0.053, see Materials and Methods, Selection of Transcription Factors). The slope of the best-fit line to the scatter plot is −3.84 (Pearson's *R* = −0.74).

To characterize the functional role of each gene cluster in macrophage activation, gene ontology (GO) information was used to identify GO term enrichments within the gene clusters (see Materials and Methods). The 460 GO term enrichments identified within the 32 gene clusters are listed in Table S8. Many of the downregulated gene clusters are enriched for cell cycle related genes (C1, C3, C7). Clusters C15, C25, and C28 appear to be enriched for cytokines–C28 includes the pro-inflammatory cytokine *Tnf* (TNFa) as well as *Ccl3*, *Ccl4*, *Cxcl1*, and *Cxcl2*; C25 includes the cytokines *Cxcl10* and *Il10*; and C15 includes the interleukin cytokine genes *Il1b*, *Il6*, and *Il12b*. Cluster C24, enriched for signal transduction genes, also includes the important cytokine *Ifnb1* (IFNb). The early-unregulated clusters, C24–28, show a high proportion of induced TFs and are enriched for TFs relative to the genome (see Table S6 and Materials and methods). Across clusters, the fraction of TFs was generally found to decrease with increasing induction time (Figure 3). Subsequent analysis focused on identifying statistically significant associations between the 80 differentially expressed TF genes and the 32 co-expressed clusters.

### Expression dynamics analysis

Noting the high proportion of induced TFs in early-upregulated clusters, we chose a signal processing technique, the time-lagged correlation (TLC), to assess potential transcriptional regulatory interactions using the time-course expression data [22], [23], [25]–[28]. The approach is based on the observation that when an induced TF affects a target gene's expression through its own differentially regulated mRNA level (and through its own differential protein expression), the induction of the target gene's mRNA expression will occur with a time lag relative to the induction of the regulator [39]–[42]. This time lag is due to the combined effects of the translation, folding, nuclear translocation, and turnover time-scales for the regulatory protein, and the time scale for elongation of the target gene mRNA. In our application of the TLC method, both the correlation magnitude and the time lag are used to assess significance, as we describe below.

**Figure 4:**
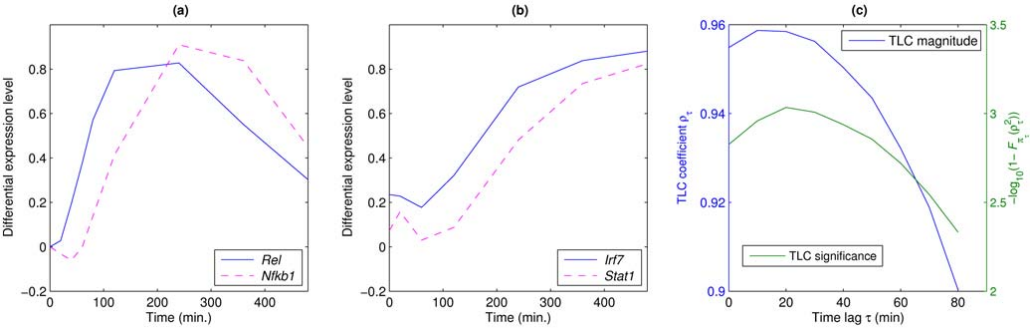
Two validated transcriptional regulatory interactions exhibiting high time-lagged correlations. (A) *Rel* and *Nfkb1*. The solid line shows the expression of *Rel* (c-REL), and the dotted line shows the expression of *Nfkb1* (p50/p105) in LPS-stimulated wild-type macrophages, over eight hours. The genes exhibit a high time-lagged correlation with a time delay of 60 minutes (across the eleven time-course experiments listed in Table S9, *ρ_τ_* = 0.91 and *P* = 0.011; see Materials and Methods, Time-lagged Correlation, for an explanation of the statistical test). The NFκB heterodimers c-REL-p50 and c-REL-p65 are known to regulate expression of *Nfkb1* [43]. The correlation at zero time lag is 0.81. (B) *Irf7* and *Stat1*. The solid line shows the expression of *Irf7* (IRF7) and the dotted line shows the expression of *Stat1* (STAT1) in LPS-stimulated *Atf3* ^(−/−)^ macrophages. The genes exhibit a high time-lagged correlation with a time delay of 20 minutes (across the ten experiments, *ρ_τ_* = 0.96 and *P* = 0.002). The transcription factor IRF7 has been shown to regulate the *Stat1* gene expression in the innate immune response to viral infection [44]. The correlation at zero time lag is 0.95. (C) Time-lagged correlation coefficient and time-lagged correlation significance measure 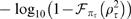 (see Equation 4) as a function of the time lag *τ*, for *Irf7* and *Stat1*. The peak value of *ρ_τ_* ^2^ occurs at *τ* = 10, but the peak significance value (taking into account the lag-specific null distribution) occurs at *τ* = 20 min.

Let *g*_1_ denote a differentially expressed TF gene, and let *g*_2_ denote a differentially expressed gene. We wish to estimate our degree of confidence in the null hypothesis, that *g*_1_ does not transcriptionally regulate *g*_2_, given time-course expression data for both genes. In the simplest case, the alternative hypothesis could be that *g*_1_ codes for a TF protein that binds the promoter of *g*_2_, thereby regulating its transcriptional activity. Let *t* be a fixed time lag for which the TLC between *g*_1_ and *g*_2_ is to be computed. Let *T* denote a set of discrete time points at which gene expression is measured, and let *T′* denote the set of time points *T*+*t*. Let *X_T_*(*g*_1_) denote the vector of expression measurements of *g*_1_ at the time points *T*, and let *X_T′_*(*g*_1_) denote the measurements of *g*_2_ at times *T′* (which can be estimated by interpolation). The time-lagged correlation (TLC) coefficient between *g*_1_ and *g*_2_ with time lag *t* is defined as
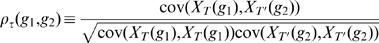
(2)where “cov” is the standard covariance. As with the standard correlation, a TLC that is close to 1 represents a perfect correlation, and a TLC that is close to −1 represents a perfect anti-correlation. This definition is easily extended to multiple time-courses (see Materials and Methods). We note that although Equation 2 is defined in terms of *g*_1_ being a TF, it can be applied to any gene pair, for example, to obtain a background distribution of TLC coefficients of gene pairs satisfying the null hypothesis. Two examples of a TF exhibiting a high time-lagged correlation with a target gene are shown in Figure 4. Both interactions (*Rel*→*Nfkb1* [43] and *Irf7*→*Stat1* [44]) correspond to known transcriptional regulatory interactions, and in both cases, the correlation with zero time lag is poorer than the correlation obtained with a time lag.

Assessing the significance of an observed sequence of time-lagged correlations between two genes (as a function of the time lag) as an indicator of possible transcriptional regulation necessitates formulating our prior expectation (i.e., prior probability distribution) for the time lag of a true transcriptional regulatory interaction. For a TF gene *g*_1_ and a target gene *g*_2_, the overall transcriptional regulatory time delay *t*_c_ (where “c” stands for the combined gene-gene delay) can be decomposed as a sum of two contributions, one for translation of the TF and post-translational processing/translocation (∼10.5±4 min [41],[45]), and one for transcription and post-transcriptional processing of the target gene (∼20–40 min [41],[42]). The total delay *t*_c_ was modeled using the gamma distribution with a mean value of 45 min and a variance of ∼250 min^2^ (see Text S1, Section 3). Because it is conditioned on the existence of a transcriptional regulatory interaction (TRI) between *g*_1_ and *g*_2_, we denote this probability distribution by *P*(*τ*_c_|*H̅*_0_) (the symbol *H̅*_0_ means that the null hypothesis, i.e., that there is no TRI, is false). This distribution was discretized to the set of time lags for which the TLC was computed, to obtain an estimate of the discrete probability for observing a given optimal time lag, *P*(*τ*|*H̅*_0_) (see Figure S7). These probabilities were then combined with the *P* value for the squared time-lagged correlation coefficient, *ρ_τ_* ^2^(*g*_1_, *g*_2_), whose derivation we describe next.

For each pair (*g*_1_,*g*_2_) for which the TLC approach was to be applied, an “optimal time lag” *θ*(*g*_1_,*g*_2_) was selected, so that a single representative TLC could be obtained for the pair. The set of time lags and the set of time-course experiments to use were selected according to a constraint (imposed to minimize interpolation error) that the target gene expression at maximum time lag must be interpolated from at least three measurements. Based on this constraint, and taking into account the expected precision at which the optimal time lag can be estimated (±5 min, based on the replicate variability in the expression data–see Materials and Methods), the set of time lags was chosen to be *t* ∈ {0, 10, 20, 30, 40, 50, 60, 70, 80 min}. Eleven time-course experiments satisfied the criteria (combining six stimuli and three genotypes, see Table S9). The TLC *ρ_τ_* ^2^(*g*_1_, *g*_2_) was computed for each of the *t* values, for each pair of genes, using data from all eleven time-course experiments combined (see Materials and Methods). The next step was to determine the optimal time lag for (*g*_1_,*g*_2_) from the squared TLC coefficient *ρ_τ_* ^2^(*g*_1_, *g*_2_). It is not ideal to simply select the *t* at which *ρ_τ_* ^2^(*g*_1_, *g*_2_) is maximal, as some studies have done [23],[26],[46], because of two competing bias effects, as we now explain. Consider a pair of genes (*h*_1_,*h*_2_) satisfying the null hypothesis, and let *t*_max_≡max(*T*), where *T* is the set of time points for a single time-course. In practice the expression of *h*_2_ cannot be extrapolated beyond *t*_max_, so the effective number of data points for computing the TLC *ρ_τ_* ^2^(*h*_1_, *h*_2_) is limited to the number of time points within *T* that are less than *t*_max_−*t*. Thus, the number of measurements that can be used to compute the TLC is *t*-dependent, and the distribution of TLCs for pairs of genes satisfying the null hypothesis depends on *t*. Therefore, one will more frequently observe (by chance) a TLC exceeding a given value (say, 0.9), by selecting the largest possible *t*. In addition, the high degree of synchronization within the transcriptional response, as well as the fact that all the SDR-transformed expression levels are zero at the initial time point, result in a second bias towards zero time lag. This effect is strengthened as the number of time points in the data set (per time-course) decreases. Therefore, selecting the optimal *t*to maximize *ρ_τ_* ^2^(*g*_1_, *g*_2_) introduces an unwanted bias towards the smallest and largest *t*values investigated (see Figure S8), and against *t* values in the middle of the range of time lags (i.e., 20–60 min).

To avoid the above-described bias, a background cumulative distribution of squared time-lagged correlation coefficient values, denoted by 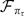 (where *p_t_* is the squared correlation *ρ_τ_* ^2^) was computed separately for each time lag *t*, using a large set *H* of gene pairs such that there is no direct transcriptional regulatory interaction (TRI) for each gene pair in the set (see Materials and Methods). The functions 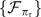 were used to select the optimal time lag *θ*(*g*_1_,*g*_2_),
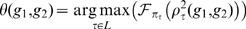
(3)and the fractional significance of the lag-specific squared correlation coefficient *ξ*(*g*_1_,*g*_2_),
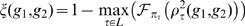
(4)Making use of the discretized distribution *P*(*τ*|*H̅*_0_) defined above, a probability ratio *R*(*τ*) was computed as the ratio of the probability of the null hypothesis (that there is no direct TRI between *g*_1_ and *g*_2_) given the measured optimal time lag, to the marginal probability of the null hypothesis,
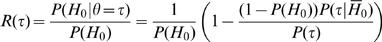
(5)It should be noted that the uncertainty in *q* due to the discretization of time lags (a practical necessity in the context of microarray-derived expression data) leads to uncertainty in the estimation of *R*(*t*). However, the effect of this uncertainty on the cluster-combined *P* value (see Equation 10 below) is small, due to the fact that time lag estimation errors for genes in a cluster are not strongly correlated. The marginal probability *P*(*τ*) was estimated from the optimal time lags of all gene pairs, and the marginal probability *P*(*H*_0_) was estimated from data in the literature (see Materials and Methods). Using this probability ratio, and in analogy with Fisher's method, a combined score for the gene pair (*g*_1_,*g*_2_) was constructed, taking into account both the optimal time lag *θ*(*g*_1_,*g*_2_) and the fractional lag-specific significance *ξ*(*g*_1_,*g*_2_),
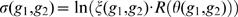
(6)Using the cumulative distribution 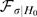 of *s* scores for gene pairs satisfying the null hypothesis, the significance of the association between *g*_1_ and *g*_2_ based on expression data can be computed,
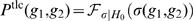
(7)This formula was applied for all pairs (*g*_1_,*g*_2_) where *g*_1_ ranged over the set of 80 TFs, *g*_2_ ranged over the set of all 1,960 differentially expressed genes, and *g*_1_≠*g*_2_ (see Materials and Methods). The expression data for the TFs are provided in Table S10 and the expression data for all 1,960 differentially expressed genes are provided in Table S4.

To estimate the overall significance (based on time-course expression data) of the association between a TF gene *f* and a cluster *C*, the *P* values *P* ^tlc^(*f*,*g*) were combined into a *P* value for the cluster, *P* ^exp^(*f*,*C*). For each pair (*f*,*C*), a Fisher score *F* ^exp^(*f*,*C*) was computed,
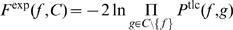
(8)where *C*\{*f*} means that if the TF gene *f* was a member of cluster *C*, the self-association *P* ^tlc^(*f*,*f*) was excluded. For each cluster *C*, the number of degrees of freedom, denoted by *d*(*C*), was estimated using *K*-means clustering (see Materials and methods). The *d*(*C*) values were used to obtain a TF-to-cluster *P* value, *P* ^exp^(*f*,*C*), using a χ^2^ test (see Materials and methods). The number of pairs for which *P* ^exp^(*f*,*C*)≤10^−3^, was 23. The differential expression levels for the strongest (TF,cluster) pairs in wild-type time-courses following stimulation by LPS (one of the time-courses used for the TLC analysis; see Table S9) are shown in Figure S9. They show a high degree of correlation between the TF gene and target cluster. The distribution of *P* ^exp^(*f*,*C*) over all TF-to-cluster pairs, and the estimated false discovery rate (FDR), are shown in Figure S10.

### Promoter scanning of co-expressed gene clusters

To provide an independent source of evidence for association between a differentially expressed TF gene and a co-expressed gene cluster, the promoters of differentially expressed genes were scanned using position-weight matrices (PWMs) representing motifs recognized by murine TFs. A motif was selected if it is recognized by at least one TF of which at least one component protein was differentially expressed in the expression dataset, ensuring that the TF had at least one expression profile that could be compared with (potential) target genes using the TLC. For each PWM, the fraction of genes with at least one above-threshold match within the promoter was computed, within a reference set of all genes detected as expressed within the TLR-stimulated macrophage, and within each co-expressed gene cluster. A total of 150 position-weight matrices were selected from the TRANSFAC database [33] for motif scanning, corresponding to the 80 differentially expressed murine TF genes (see Table S5, and Materials and Methods). Promoter sequences 2 kbp upstream of the transcription start site were obtained for 1,713 out of the 1,960 differentially expressed genes, and for 7,492 out of 8,788 expressed genes (used as a reference set; see Materials and Methods) from the UCSC genome annotation database [47]. For each PWM, a minimum match score was determined at which the PWM had a match on average once per 10 kb, within a set of 7,503 promoter sequences for genes not detectably expressed in the macrophage (to avoid biasing the match score threshold calculation with true TF targets; see Materials and Methods). Using these PWM match score thresholds, promoters were scanned within the reference set of genes, and within each co-expressed cluster of genes. The distribution of distances of matches from the transcription start site (Figure S11) has a median of 537 bp.

**Figure 5:**
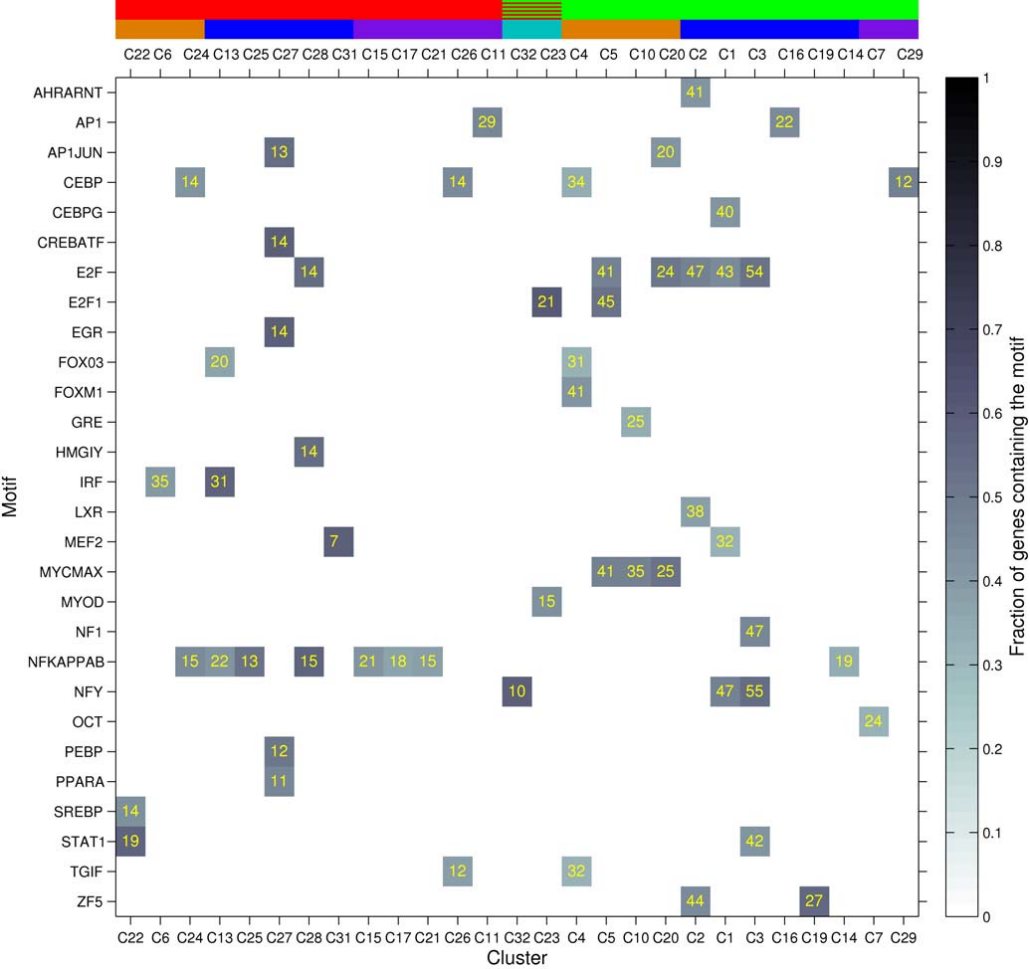
Patterns of high-confidence motif enrichments within promoters of target clusters reveal associations between regulatory elements and expression patterns. Each row in the matrix represents a TF binding element, and each column represents a cluster of differentially expressed genes. Clusters are ordered as in Figure 2, and thus are grouped hierarchically by similarity of their extremal expression fold-change under the four TLR agonists LPS, Pam_3_CSK_4_, poly I:C, and R848. Each motif (row) is associated with one or more position-weight matrices (the V$ prefix and numeric suffixes are omitted, and results for multiple position-weight matrices representing the same motif were combined for each column, by taking the matrix with the maximum number of matches within the indicated cluster). Each colored block in the matrix indicates pair of a motif and target cluster for which the fraction of genes in the cluster with a motif match, is enriched relative to the overall fraction of genes expressed in the macrophage that possess the motif (*P*≤10^−2^, Fisher's exact test). The color of each matrix element (block) in the interior of the figure indicates the fraction scanned of genes within the cluster containing at least one match for the indicated motif. The number of scanned genes within the cluster that contained a match for the indicated motif is shown in yellow typeface. The red/green colored blocks above the top horizontal axis shows whether each cluster is upregulated (red) or downregulated (green) at its most extremal fold-change under stimulation with the aforementioned TLR agonists. The hatched green/red pattern indicates a cluster whose extremal fold-change direction (up/down) is stimulus-dependent (see Figure 2). The colored (blue, cyan, orange, yellow, purple) blocks above the top of the matrix indicate the likely pathway through which the cluster is differentially expressed; the color scheme corresponds to that shown in the dendrogram in Figure 2.

As a next step towards inferring a transcriptional network, enrichments of TFBS motifs were computed for individual gene clusters. For each cluster *C* and position-weight matrix *m*, enrichment statistics were computed based on the fraction of genes in *C* possessing at least one match for *m*. For each pair (*m*,*C*) for which the fraction of genes containing a match for *m* within the cluster *C* was greater than in the reference set of genes, a *P* value was computed using Fisher's exact test (see Materials and Methods, and [48]) and denoted by *P* ^scan^(*m*,*C*). This *P* value represented the significance of the enrichment of matrix *m* within the promoters of cluster *C*, relative to the reference set of promoters (expressed genes). A matrix representation of the strongest motif enrichments (56 associations with *P* ^scan^(*m*,*C*)≤10^−2^) with the clusters grouped by expression similarity (Figure 5) reveals several associations between TF motifs and patterns of differential expression. First, NFκB and IRF recognition elements are associated with upregulated clusters, while E2F and MYCMAX elements are associated with downregulated clusters. The IRF element was strongly associated with TRIF-dependent cluster C6 and STAT1 was strongly associated with C22. Many TF motifs were associated with the core early response cluster C27, including AP1, CREB/ATF, EGR, PEBP, and PPARA. The quantitative results of the cluster-wise statistical tests (numbers of matches and *P* values) are provided in Table S11.

To enable integration of the promoter scanning evidence with the time-lagged correlation evidence, PWMs that were enriched for matches within gene clusters, were mapped to differentially expressed TF genes as follows. For each PWM *m*, a list of genes coding for TFs (or TF components) that bind the motif corresponding to *m* were obtained from a TRANSFAC-derived mapping (see Materials and Methods). For each TF gene *f* and cluster *C*, a *P* value for the association between *f* and *C* based on promoter scanning evidence, *P* ^scan^(*f*,*C*), was defined as the minimum over all *P* ^scan^(*m*,*C*) for all matrices *m* that are associated with the TF gene *f*. The distribution of the resulting *P* values and the false discovery rate (as a function of *P* value) are shown in Figure S12. A total of 31 factor-to-cluster associations were identified with *P* ^scan^(*f,C*)≤10^−3^, indicating a statistical power that is slightly higher than with the TLC-based evidence.

### Data integration and network extraction

To identify the set of all possible TF gene-to-target interactions consistent with motif scanning evidence, for each TFBS motif match within the promoter of a target gene, the time-lagged correlation was computed for all possible TF genes that map to the TFBS motif. The resulting list of 54,253 pairs (*f*,*g*) of TF gene *f* and target gene *g*, provided as Table S12, shows that many known transcriptional regulatory interactions have high ranking based on time-lagged correlation–for example, NFκB/*Rel* associated with *Icam1* [49] and *Cebpd* associated with *Il6* [50]. Although the TLC-ranked list of motif targets has some potential utility for identifying specific transcriptional regulatory interactions, even the high-ranking elements of the list will contain many false positives (and will miss many true transcriptional regulatory interactions) due to the uncertainty in motif PWMs and the prevalence of post-translational regulation that may obscure the time-lagged correlation. Therefore, further data reduction is necessary to gain insight into the global transcriptional program of the TLR-stimulated macrophage. By using a statistical test that compares the relative frequency of motif occurrence within a cluster relative to a background set of genes, a more reliable estimate of TF association with a co-expressed cluster can be obtained.

To construct a combined transcriptional network of the TLR-stimulated macrophage, *P* values for associations between TF genes and co-expressed gene clusters based on expression dynamics and promoter scanning were combined. For each pair (*f*,*C*) where *f* is one of 80 TF genes and *C* is one of 32 gene clusters, a combined *P* value *P* ^comb^(*f*,*C*) was computed from the *P* values for the scanning and expression evidences, *P* ^scan^(*f*,*C*) and *P* ^exp^(*f*,*C*). The *P* values were combined using Fisher's method (see Materials and Methods), a standard tool for meta-analysis of independent tests of a hypothesis [51]. TF-cluster pairs were then ordered by increasing *P* value *P* ^comb^(*f*,*C*), and a cutoff was selected so that the estimated false discovery rate did not exceed 0.025 (resulting in a cutoff *P* ^comb^(*f*,*C*)≤0.0248). Additionally, two filtering criteria were imposed: (i) *P* ^scan^(*f*,*C*)≤0.05, to ensure that there is a minimal enrichment of TFBS; and (ii) a cluster-average optimal time lag between *f* and *C* that was greater than 10 min, i.e., 〈*θ*〉*_f,C_*≥10 min (see Materials and Methods). A scatter plot of the *P* values for the two evidences is shown in Figure S13, and indicates that for the data points that were rejected based on the *P* ^comb^(*f*,*C*) cutoff, no dependency between the evidences is evident. A total of 90 interactions involving 36 TF genes and 27 clusters (comprising 86% differentially expressed genes), were accepted based on the above criteria (see Table 1). If the TLC *P* values were not included, and if the same rate of false discovery were imposed, the network would be significantly less parsimonious (∼150 interactions), due to the large number of TF gene families that map to a common motif. Overall network coverage was estimated by taking the fraction of differentially expressed genes that (i) are members of the 27 clusters in the network; and (ii) possess a match for a motif recognized by one or more of the TFs associated with the cluster. From this estimate the network contains 1,232 genes, or 63% of the 1,960 genes that are differentially expressed under TLR stimulation.

**Table 1:** Network of inferred transcription factor–cluster associations

Clust	TF	TF Clust	-log_10 _ *P* ^scan^	Position-Weight Matrix Name	FracBind	Scan Hits	-log_10_ *P* ^exp^	<*θ*>	Mean Corr
1	*Cebpg*	22	5.74	V$CEBPGAMMA_Q6	0.41	40	0.03	13.5	−0.21
1	*E2f1*	2	2.84	V$E2F_02	0.44	43	0.82	75.4	0.72
1	*E2f7*	3	1.41	V$E2F1_Q4_01	0.41	40	4.42	73.4	0.86
1	*Irf2*	13	1.60	V$IRF_Q6_01	0.32	31	1.35	68.8	−0.74
1	*Irf7*	6	1.60	V$IRF_Q6_01	0.32	31	3.26	68.7	−0.83
1	*Isgf3g*	6	1.60	V$IRF_Q6_01	0.32	31	2.13	71.3	−0.80
1	*Mef2a*	2	2.73	V$MEF2_Q6_01	0.33	32	1.50	75.0	0.78
1	*Mef2c*	16	2.73	V$MEF2_Q6_01	0.33	32	2.23	67.4	0.81
1	*Nfyc*	4	6.08	V$NFY_Q6	0.48	47	0.17	78.1	0.59
2	*E2f1*	2	3.68	V$E2F_02	0.46	46	3.16	34.2	0.85
2	*E2f6*	10	2.97	V$E2F_03	0.47	47	2.72	65.9	0.82
2	*E2f7*	3	2.97	V$E2F_03	0.47	47	3.62	33.1	0.85
2	*Myc*	20	1.95	V$MYCMAX_01	0.34	34	1.36	18.4	0.74
2	*Rxra*	14	2.55	V$LXR_DR4_Q3	0.38	38	1.61	57.6	0.77
3	*E2f1*	2	3.86	V$E2F_Q6_01	0.53	54	2.39	47.2	0.83
3	*E2f6*	10	3.86	V$E2F_Q6_01	0.53	54	2.26	69.0	0.81
3	*E2f7*	3	3.86	V$E2F_Q6_01	0.53	54	8.00	33.5	0.93
3	*Myc*	20	1.32	V$MYCMAX_03	0.32	33	1.62	16.1	0.78
3	*Nfic*	19	3.98	V$NF1_Q6	0.46	47	0.26	77.6	0.63
3	*Nfyc*	4	7.73	V$NFY_Q6_01	0.54	55	0.28	58.3	0.65
3	*Rxra*	14	1.36	V$LXR_DR4_Q3	0.33	34	1.24	64.0	0.77
3	*Stat1*	6	2.12	V$STAT1_03	0.41	42	2.95	12.1	−0.86
4	*Cebpa*	19	2.05	V$CEBP_Q2	0.34	34	0.43	70.0	0.65
4	*Foxm1*	3	6.18	V$FOXM1_01	0.41	41	0.63	52.6	0.68
4	*Mef2a*	2	1.68	V$MEF2_04	0.30	30	1.56	40.2	0.79
4	*Myc*	20	1.99	V$MYCMAX_B	0.44	44	0.63	45.5	0.68
4	*Nfyc*	4	1.43	V$NFY_Q6_01	0.36	36	1.06	68.0	0.74
4	*Tgif1*	27	3.12	V$TGIF_01	0.32	32	0.16	42.5	0.10
5	*E2f1*	2	3.85	V$E2F1_Q6_01	0.52	45	2.31	50.3	0.81
5	*E2f6*	10	2.71	V$E2F_03	0.48	41	2.84	72.5	0.81
5	*E2f7*	3	2.71	V$E2F_03	0.48	41	1.27	47.3	0.76
5	*Myc*	20	2.54	V$MYCMAX_B	0.48	41	1.00	27.9	0.69
5	*Rxra*	14	1.87	V$PPARA_02	0.30	26	1.72	63.7	0.77
6	*Irf1*	25	3.65	V$IRF_Q6	0.40	35	0.09	79.1	0.56
6	*Irf2*	13	3.65	V$IRF_Q6	0.40	35	3.32	44.3	0.81
6	*Irf3*	12	3.65	V$IRF_Q6	0.40	35	0.05	35.4	−0.09
6	*Irf5*	6	3.65	V$IRF_Q6	0.40	35	1.21	68.4	0.75
6	*Irf7*	6	3.65	V$IRF_Q6	0.40	35	5.20	26.6	0.88
6	*Isgf3g*	6	1.84	V$IRF_Q6_01	0.33	29	3.24	25.0	0.84
7	*Pou2f2*	9	2.10	V$OCT_C	0.32	24	0.42	45.9	−0.64
9	*Myc*	20	1.54	V$MYC_Q2	0.36	26	1.26	29.3	−0.67
10	*Atf1*	14	1.73	V$CREB_Q3	0.34	25	0.86	38.1	0.73
10	*Myc*	20	2.18	V$MYCMAX_B	0.47	35	0.43	11.2	0.68
10	*Nfyc*	4	1.67	V$NFY_Q6_01	0.39	29	1.31	35.8	0.78
10	*Nr3c1*	6	2.79	V$GR_Q6_01	0.34	25	1.86	48.5	−0.79
11	*Fos*	27	3.23	V$AP1_Q2_01	0.45	29	0.11	47.0	−0.05
11	*Jun*	20	3.23	V$AP1_Q2_01	0.45	29	0.20	41.4	−0.37
11	*Junb*	28	3.23	V$AP1_Q2_01	0.45	29	0.05	70.3	0.24
13	*Foxo3a*	14	2.41	V$FOXO3_01	0.37	20	0.77	14.2	−0.75
13	*Irf1*	25	7.47	V$IRF_Q6_01	0.57	31	0.32	77.5	0.64
13	*Irf3*	12	7.47	V$IRF_Q6_01	0.57	31	0.07	42.8	0.07
13	*Irf5*	6	7.47	V$IRF_Q6_01	0.57	31	0.49	22.7	0.71
13	*Nfkb1*	15	2.32	V$NFKB_Q6_01	0.41	22	2.41	31.3	0.82
13	*Rel*	25	1.65	V$CREL_01	0.37	20	0.88	71.9	0.72
14	*Nfkb1*	15	2.14	V$NFKB_C	0.34	19	3.37	51.3	−0.84
15	*Rel*	25	2.42	V$CREL_01	0.42	21	1.78	29.0	0.78
16	*Cebpa*	19	1.43	V$CEBP_C	0.32	16	1.05	73.4	0.74
16	*E2f1*	2	1.78	V$E2F_01	0.40	20	2.52	52.3	0.80
16	*Jun*	20	2.39	V$AP1_Q2_01	0.44	22	0.14	59.3	0.43
16	*Myc*	20	1.90	V$MYCMAX_02	0.40	20	0.83	20.5	0.63
16	*Rxra*	14	1.32	V$FXR_IR1_Q6	0.30	15	1.33	63.8	0.75
17	*Nfatc1*	14	1.71	V$NFAT_Q4_01	0.36	18	1.78	33.6	−0.79
17	*Nfatc2*	14	1.71	V$NFAT_Q4_01	0.36	18	1.60	12.2	−0.80
17	*Nfkb1*	15	2.02	V$NFKB_Q6	0.36	18	2.25	60.3	0.80
17	*Sfpi1*	17	1.35	V$ETS_Q6	0.42	21	1.34	14.8	0.78
18	*Pou2f2*	9	1.78	V$OCT_Q6	0.33	16	0.68	53.9	−0.67
19	*Nr3c1*	6	1.62	V$PR_Q2	0.33	16	1.24	37.2	−0.74
19	*Rxra*	14	1.61	V$T3R_Q6	0.37	18	1.65	14.6	0.79
19	*Zfp161*	19	2.98	V$ZF5_01	0.55	27	0.90	49.8	0.73
20	*E2f7*	3	2.11	V$E2F_03	0.50	24	0.64	66.5	0.66
20	*Myc*	20	2.36	V$MYCMAX_B	0.52	25	0.66	53.6	0.64
21	*Nfkb1*	15	2.13	V$NFKAPPAB_01	0.38	15	2.25	28.2	0.78
22	*Stat1*	6	3.22	V$STAT1_01	0.58	19	0.16	55.2	0.39
23	*E2f1*	2	3.24	V$E2F1_Q4_01	0.60	21	0.08	47.6	0.54
23	*E2f6*	10	3.24	V$E2F1_Q4_01	0.60	21	0.48	46.0	0.66
23	*E2f7*	3	3.24	V$E2F1_Q4_01	0.60	21	0.05	50.7	0.49
25	*Irf1*	25	1.41	V$IRF_Q6_01	0.40	10	1.42	15.1	0.79
26	*Cebpa*	19	2.50	V$CEBP_01	0.45	14	0.01	52.6	0.14
26	*Tgif1*	27	2.14	V$TGIF_01	0.39	12	0.28	49.4	0.36
27	*Atf1*	14	2.57	V$CREBATF_Q6	0.58	14	0.25	69.0	0.52
27	*Cbfb*	4	2.39	V$PEBP_Q6	0.50	12	0.45	65.3	0.52
27	*E2f7*	3	1.62	V$E2F_Q4_01	0.54	13	1.01	61.0	0.63
27	*Egr1*	27	2.62	V$KROX_Q6	0.58	14	1.37	16.0	0.75
27	*Egr2*	27	2.62	V$KROX_Q6	0.58	14	1.16	11.7	0.75
27	*Jun*	20	2.63	V$CREBP1CJUN_01	0.54	13	0.24	47.3	0.46
27	*Rxra*	14	2.46	V$PPARA_02	0.46	11	0.61	63.3	0.53
28	*E2f1*	2	2.75	V$E2F_01	0.54	14	0.05	37.9	−0.05
28	*Nfkb1*	15	4.48	V$NFKAPPAB_01	0.58	15	0.07	25.7	0.27
29	*Cebpa*	19	2.48	V$CEBP_01	0.48	12	0.04	65.9	−0.11
31	*Mef2a*	2	2.39	V$MEF2_03	0.58	7	0.16	35.9	−0.51

Column 1 indicates the target gene cluster. Column 2 indicates the transcription factor gene that is associated with the cluster, based on the two sources of evidence. Column 3 indicates the cluster of which the transcription factor gene is a member. Column 4 indicates the -log_10_ *P* value of the promoter scanning-based evidence. Column 5 indicates the name of the position-weight matrix that had the smallest scanning-based *P* value of association with the cluster, for the indicated transcription factor gene (the “V$” prefix is not shown). Column 6 indicates the fraction of scanned genes within the cluster that had at least one match for the indicated position-weight matrix. Column 7 contains the number of scanned genes within the cluster that had at least one match for the indicated position-weight matrix. Column 8 indicates the negative log_10_ *P* value of the time-lagged correlation evidence. Column 9 indicates the cluster-wide average time lag *θ* with respect to the indicated transcription factor gene. Column 10 contains the average optimal time-lagged correlation between the indicated transcription factor gene, and the genes within the cluster.

The distribution of the number of targets regulated by TFs, the so-called out-degree distribution of the transcriptional network, is one key measure of the network's interconnectedness [52]. For each TF that was included in the transcriptional network, the number of targets was estimated using the promoter scanning data (see Materials and Methods). The out-degree varied approximately 20-fold over the set of 36 TF genes (Figure S14). The transcription factor MYC (which is involved in development and cellular differentiation [53]) was found to be the most highly connected in the network (consistent with the high out-degree for MYC found in [11]), followed by members of the E2F family of TFs (believed to play a role in cell cycle regulation [54]). Other highly connected TFs include NFYC (a repressor in the TGFβ signaling pathway [55] and member of a TF family involved in monocyte differentiation [56]) and RXRA (a component of heterodimeric TFs that regulate inflammatory signaling and cholesterol metabolism [57]). Also strongly connected in the network are the NFκB TF family members cREL and NFKB1/p50 (key early regulators of the immune response [58]); the IRF family members IRF1, IRF3, IRF5, IRF7, and IRF9 (regulators of interferon-induced immune response [17]); and STAT1 (a key regulator of apoptosis and mediator of interferon signaling [59]). Both the IRF and E2F family TFs had strong *P* values for association with target clusters (Figure S14). The out degree distribution appears to be scale-free, consistent with previous reports for mammalian networks [11],[60]. The number of TF genes associated each cluster (in degree) ranged from 1 to 9, with an average in-degree of 3.3.

**Figure 6:**
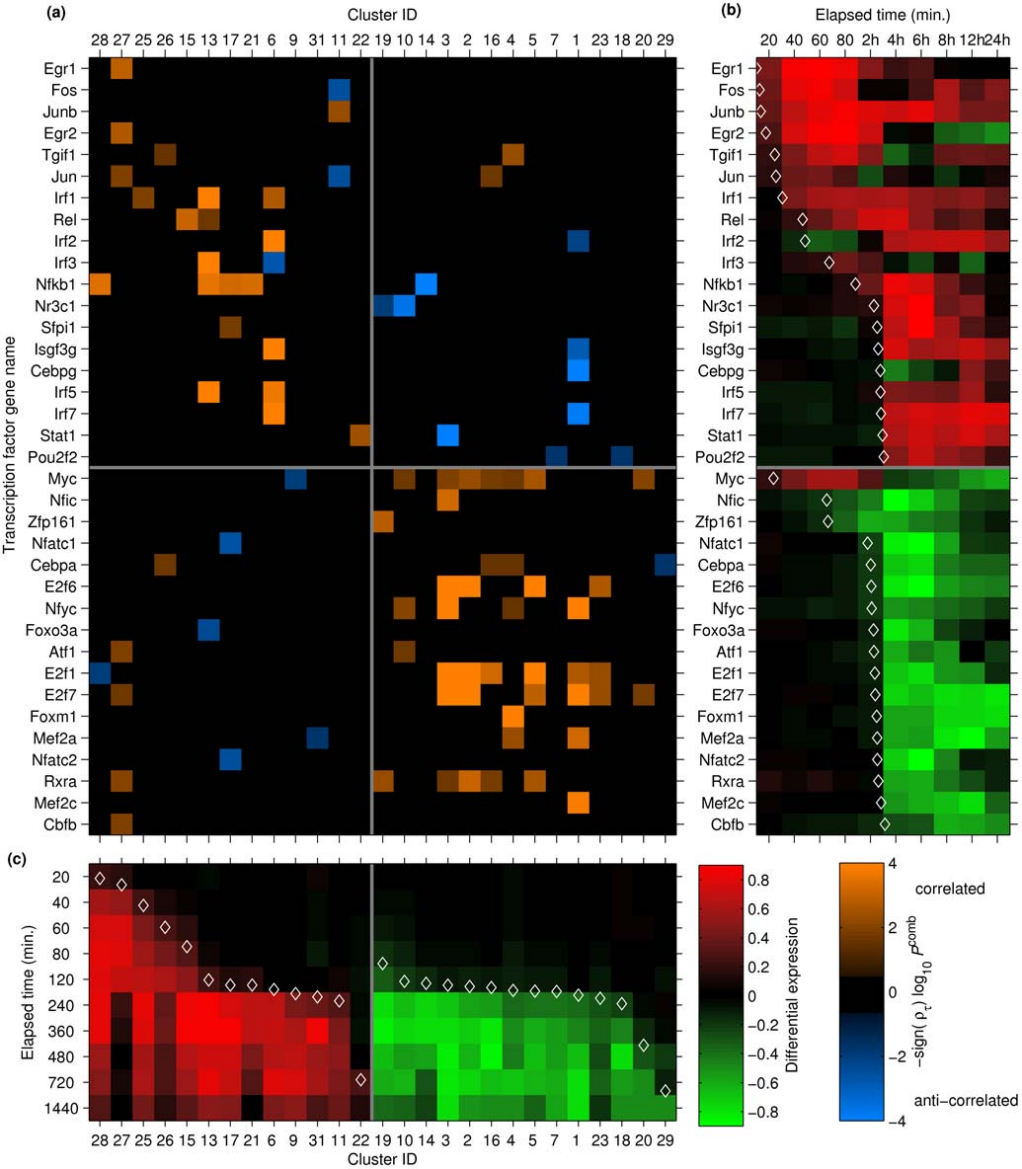
Transcription factor genes associated with clusters in the inferred transcriptional network. (A) The matrix shows associations between transcription factor genes and co-expressed gene clusters. Each column represents one of the 27 clusters within the inferred network, and each row represents one of the 36 transcription factor genes in the network. Clusters are ordered based on the LPS response time, defined as the time (under LPS stimulation) at which the cluster-median differential expression level reaches 25% of the maximum differential expression (see Materials and Methods, Expression Clustering). Transcription factor genes are ordered based on the LPS response time. The vertical gray line separates upregulated clusters (left half) from downregulated clusters (right half). The horizontal gray line separates upregulated transcription factors (top) from downregulated transcription factors (bottom). An orange or blue square indicates a statistically significant association between the transcription factor gene and the cluster, based on both promoter scanning and expression dynamics. An orange solid rectangle represents a positive average time-lagged correlation with genes in the cluster; a blue solid rectangle represents a negative average time-lagged correlation. (B) The red-green matrix is a heat-map showing transcription factor gene expression. The color indicates the normalized differential expression of the indicated transcription factor gene (over time), in LPS-stimulated wild-type macrophages (SDR, see Equation 1). Red indicates upregulation relative to unstimulated macrophages and green indicates downregulation. A diamond symbol indicates the transcription factor response time. (C) Each column of the red-green matrix indicates the median normalized differential expression of the genes in the indicated cluster (over time), in LPS-stimulated wild-type macrophages. The diamond indicates the average LPS response time of the genes within the cluster.

To reveal patterns among TFs that may regulate multiple clusters, the connections between the 36 TFs and the 27 clusters in the inferred network were arranged in a matrix in which each row represents an induced TF and each column represents a cluster of differentially expressed genes (Figure 6). Both the TFs and clusters were divided into subsets that are induced or repressed under LPS stimulation, and ordered within these subsets based on the time of 25% differential expression under LPS (see Materials and Methods). Thus, the matrix is divided into quadrants; for example, the upper left quadrant contains connections between induced TF genes and induced clusters, and the lower-right quadrant contains connections between downregulated TF genes and downregulated clusters. The upper left and lower right quadrants contain primarily positive correlations, with most anti-correlated connections found in the upper right and lower left quadrants. In the upper left quadrant, the connections generally fall along an arc indicating the temporal sequence of TF gene activation. The anti-correlated “off arc” connections within this quadrant generally indicate the association between the falling edge of a transiently induced TF gene and the rising edge of a late-induced gene cluster. The only correlated “off arc” connections within this quadrant (*Nfkb1*→C28, and *Junb*→C11) have weak time-lagged correlation evidence, but a very significant motif scanning *P* value. In contrast, the downregulated gene clusters and TF genes are not as stratified as the upregulated clusters in terms of the time of differential expression, and thus associations appear throughout the lower-right quadrant.

The network of associations between TF genes and clusters (based on combined scanning and expression evidence) directly leads to hypotheses regarding TF regulation of clusters. For example, a statistical association between any of the TF genes *Jun*, *Junb*, or *Fos* and a cluster would suggest a hypothesis that the TF AP1 regulates that cluster. The network also recapitulates several known transcriptional regulatory interactions. First, the NFκB component *Rel* is associated with C15, which is enriched for cytokines and contains many NFκB targets including *Nfkb1* [43], *Il6*, and *Il12b* [6]. Second, *Jun*, a component of AP1 (a regulator of stress response such as response to ultraviolet radiation or pathogenic insult [61]), is associated with C27, an early-upregulated cluster that is enriched for cell cycle-related genes and genes involved in the DNA damage response. Furthermore, C27 contains *Egr1*, which is a known target of AP1 under genotoxic stress conditions [61]. Third, IRF1 is strongly associated with the antiviral cluster C13, which contains the validated IRF1 target gene, *Ccl5* [62]. The network also includes the TF genes *Egr1* (a key regulator of LPS-induced cytokine signaling [63]) and *Egr2* (implicated in adhesion and phagocytosis [64] as well as cell proliferation [65]) as regulators of C27. Finally, the TF gene *Sfpi1* (PU.1) is associated with C17, an induced gene cluster enriched for endosome-associated genes (PU.1 over-expression is known to block viral escape from the endosome [66]).

Several interactions in the network were detected only through the integration of expression data with promoter scanning evidence. For example, based on scanning evidence alone, with a FDR of 0.1 (*P* ^scan^≤0.0033), the association between *Nfkb1* and C17 would not have been detected, but by including the effect of the strong TLCs between *Nfkb1* and C17 genes, an association between *Nfkb1* and C17 was detected. As a second example, the network includes an association between the TF gene *Irf1* and cluster C25; based on promoter scanning evidence alone, only a general association of the IRF family with the cluster would have been possible (see Table 1).

In order to investigate the possible co-operative regulation of clusters by TFs in the network, protein interactions were obtained for human orthologs of protein units associated with the 36 TF genes shown in Figure 6. Protein interactions between the TFs were obtained from the Human Protein Reference Database [67] and the Biomolecular Interaction Network Database [68] (see Materials and Methods). The resulting interaction diagram, shown in Figure S15, reveals that upregulated TFs are highly interconnected at the level of protein-protein interactions [6]. Furthermore, the diagram shows 15 pairs of interacting TFs whose corresponding genes co-associate with clusters in the network. An example corresponding to a known transcriptional complex is the pair c-JUN (an AP1 component) and EGR1 [69]; both are associated with C27.

A notable induced TF gene in the network is *Tgif1* (TGIF1, or TG-interacting factor 1, named for the core TGIF1 binding sequence, 5′-TGTCA-3′ [70]), a transcriptional repressor in the TGFβ signaling pathway [71]. TGIF1 has not been previously implicated in classical macrophage activation. It is associated (*P* ^scan^<0.01) with C26, a cluster containing genes involved in immune response, ubiquitin cycle, and leukocyte activation. Specifically, C26 contains the cytokines *Csf2* (which stimulates differentiation of macrophages and granulocytes, and is pro-inflammatory [72]) and *Gm1960* (a mediator of neutrophil chemotaxis [73]). The *Csf2* promoter appears to have a TGIF1 binding site motif match (match score>0.96) in the region (−254,−244) relative to the transcription start site, and *Gm1960* also has three TGIF1 motif matches approximately 1.5 kbp upstream of the start site (best match score>0.95). In humans, TGIF1 is known to interact with several protein members of the SMAD/AP1 transcriptional complex (Figure S16) [71],[74].

To validate the microarray-based expression measurement, *Tgif1* expression was measured in murine BMMs using quantitative PCR (qPCR; see Materials and Methods). Consistent with the microarray-based results, *Tgif1* expression was found to be ∼3-fold upregulated after 1 hour of stimulation by LPS or Pam_3_CSK_4_ (data not shown). Furthermore, from microarray-based measurement (Affymetrix probeset 1422286_a_at), *Tgif1* expression is ∼2.4-fold reduced in unstimulated *Ticam1* ^(Lps2/Lps2)^ BMMs relative to wild-type (with no apparent effect in *MyD88* ^(−/−) ^BMMs relative to wild-type), suggesting that basal expression of *Tgif1* is TRIF-dependent.

### Targeted validation using ChIP-on-chip

Genome location analysis based on chromatin immunoprecipitation-on-chip (ChIP-on-chip) hybridization was used to validate five high-confidence associations in the transcriptional network, between NFκB/p50 and clusters C13, C17 and C28; and between IRF1 and clusters C13 and C25. This validation consisted of demonstrating a statistical enrichment of ChIP-on-chip–identified binding for a given TF among genes within a cluster with which the TF was associated through our computational method, as compared to randomly selected TLR-responding genes. A custom-fabricated oligonucleotide microarray was used, with probes tiling up- and downstream of genes that were differentially expressed under TLR stimulation in a murine macrophage-like cell line (see Materials and Methods). Macrophages were stimulated with LPS and then ChIP was carried out using TF-specific antibodies at 1 and 2 h, and (for IRF1 only) 4 h. Binding of p50 was highly enriched within the genes of clusters C13 and C28 represented on the tiling array (18 out of 23 and 20 out of 21 genes were bound, respectively) but not significantly enriched for C17 (11 out of 20). IRF1 binding was enriched within the genes of C13 and C25 (18 out of 23, and 18 out of 22, respectively). In four out of five cases, the enrichment relative to the overall rate of binding to differentially expressed genes represented on the tiling array satisfied *P*<0.01 (Fisher's Exact Test; see Table 2). ChIP-on-chip results for individual target genes within the aforementioned clusters are provided in Table S13, and results for all clusters that were represented on the array (see Materials and Methods) are shown in Table S14. For each of the two TFs assayed with ChIP-on-chip, and for those clusters that were identified as targets of the TF through the network analysis, the fraction of clusters found to have significant TF binding to their genes was higher than for clusters selected randomly from among all clusters represented on the tiling array (1.7-fold overall). Additionally, the association between IRF1 and C30 was significant (*P*<0.05) based on scanning, but not significant based on *P* ^comb^. Consistent with the integrated analysis, C30 was not significantly enriched for IRF1 binding, based on the ChIP-on-chip assay.

**Table 2:** Validation of transcription factor-to-cluster associations using ChIP-on-chip

TF	Matrix	Stim.	Clust	Time Points	In Clust	On Chip	Bound	*P*-Value
NFκB/p50	NFKB_Q6	LPS	C13	1 h, 2 h	64	23	18	1.1×10^−3^
NFκB/p50	NFKB_Q6	LPS	C17	1 h, 2 h	58	20	11	2.5×10^−1^
NFκB/p50	NFKAPPAB_01	LPS	C28	1 h, 2 h	28	21	20	1.1×10^−6^
IRF1	IRF_Q6_01	LPS	C13	1 h, 2 h, 4 h	64	23	18	2.3×10^−3^
IRF1	IRF_Q6_01	LPS	C25	1 h, 2 h, 4 h	37	22	18	8.8×10^−4^

Shown are five (TF,cluster) associations for which at least 30% of the genes within the cluster are represented on the tiling array, along with the results of the ChIP-on-chip assay for binding of the indicated TF to the promoters of genes within the indicated cluster. Column 1 indicates the transcription factor antibody target. Column 2 indicates the position-weight matrix that was used for scanning the promoters of genes in the cluster. Column 3 indicates the stimulus used. Column 4 indicates the gene cluster whose promoters the indicated TF is predicted to bind. Column 5 indicates the time points at which ChIP-on-chip assays were performed. Column 6 indicates the number of genes in the cluster. Column 7 indicates how many of these genes have probes tiled on the chip, in the flanking 5′ intergenic region (due to the much smaller microarray expression dataset used to select genes for the tiling array, only about 22% of the 1,960 differentially expressed genes were represented on the tiling array, as described in Materials and Methods). Column 8 indicates the number of these genes that were identified positively by ChIP-on-chip as having the indicated transcription factor bound to chromatin, in the 5′ flanking intergenic region. Column 9 indicates the *P* value for the enrichment of ChIP-on-chip hits among genes within the cluster identified by promoter scanning, as compared to the set of all genes on the array (Fisher's exact test). The ChIP-on-chip results for individual genes are provided in Table S13.

## Discussion

In this study we inferred a transcriptional network underlying dynamic TLR-stimulated activation of the murine macrophage. This network consists of statistical associations between differentially expressed transcription factor (TF) genes and co-expressed clusters of genes, each indicating a possible role for the associated TF in regulating the cluster. Such associations have proved useful for generating and prioritizing testable hypotheses regarding transcriptional regulation [6],[7]. A novel computational approach was used that combined sequence- and expression-based evidence. Using expression data acquired under a comprehensive set of TLR stimuli (and sampled densely in time), differentially expressed genes were partitioned into clusters of co-expressed genes that revealed a diversity of induction time scales, functional enrichments, and stimulus-dependent activation patterns. The clustering enabled sensitive identification of TFBS enrichments despite uncertainty (due to limited sampling) in the position-weight matrices and in the appropriate score threshold for motif scanning. In addition, using the SDR-normalized expression data for clustering ensured that genes were clustered based on their temporal (and stimulus-dependent) activation profiles, rather than by the magnitude of fold-change. Early-upregulated clusters were found to be enriched for TFs, consistent with the idea that many regulators of the transcriptional program are themselves produced on-demand in response to TLR stimulation [6]. The early induction of a large number of TFs was an important indicator of the potential utility of analyzing temporal expression as an evidence for transcriptional regulatory interactions (TRIs).

The time-lagged correlation (TLC) was used to analyze temporal gene expression for TFs and gene clusters, and in addition to the correlation strength, the biological plausibility of the estimated optimal time lag was factored into the significance assessment for the TLC. This time lag is useful for distinguishing between genes that are linked by a regulatory interaction and genes that are merely co-expressed. The TLC is efficient to compute, and in general requires fewer measurements than methods that rely on estimating the joint probability distribution of the expression of two genes (e.g., pairwise mutual information [11]). This observation is related to the most notable drawback of the TLC, namely, that it is sensitive only to the covariance of the joint probability distribution, and not higher order moments (with significantly more expression measurements, a possible extension of this method could be to use time-lagged mutual information [75]). A second limitation of the TLC (and of any evidence based solely on lagged expression comparison) is that in practice it can be difficult to distinguish between indirect transcriptional regulation through a rapid-acting intermediary, and direct transcriptional regulation. Finally, while it is not a significant issue in the cluster analysis described in this work, we note that the proposed method for estimating the significance of the expression data for a single gene pair (Equation 6) is potentially not robust with respect to noise in the data. For the purpose of single-gene analysis, it could be improved by using a polynomial fit to the *τ* dependence of 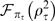, or by defining the optimal time lag to be the time lag that minimizes *σ*.

The specific implementation of the TLC approach used in this study has two key advantages. First, by selecting the optimal time lag for a TF–gene pair based on minimizing the lag-dependent *P* value rather than maximizing the squared correlation coefficient, the inherent bias of the TLC technique in selecting time lags was avoided. This made it possible to include the contributions of (i) the magnitude of the correlation, and (ii) the probability of observing the optimal time lag, to the significance of a pairwise association. Second, the probability distribution for time lags among true interactions was incorporated as a prior in the significance calculation. This enabled taking into account the biological plausibility of the time lag in computing the significance. This significance test for the TLC has not, to our knowledge, been previously reported.

With any network inference method based on pair-wise comparison of the expression profiles of a regulator and a possible target (including the TLC method), it is difficult to accurately resolve the multi-factorial control of a target gene. This is particularly true when the effect of one TF is simply to modulate (amplify or dampen) the time-varying influence of another TF on a target gene. Several additional mechanisms can confound or eliminate the correlation between the expression level of a TF gene and the chromatin-bound activity of the corresponding TF, including multimeric TF assembly from protein products of several genes, post-translational activation of the TF, dynamically regulated nuclear translocation, and dynamically regulated TF protein turnover. For example, in the case of ATF3, there is little correlation between differential expression and nuclear localization [6], and as a result, this TF is not strongly implicated in the network via TLC. However, we note that the CREB/ATF binding motif was identified as enriched within the core early response cluster C27. Additionally, we note that given that the expression data set used in this work is densely sampled at early times (1–2 hours) and sparsely sampled at late times, our ability to leverage expression data as an evidence for TRI is reduced for very late-responding TF genes (e.g., *Lmo2*). In summary, with a limited expression dataset, a high-significance TLC by itself should not be regarded as sufficient evidence to infer a TF-to-target association, underscoring the importance of incorporating additional sources of evidence.

In the present work, promoter sequence scanning was used to identify TFBS motifs enriched within co-expressed gene clusters. Due to the often one-to-many mapping between TFBS motifs and TFs, the scanning-based evidence often identifies multiple candidate TFs with a gene cluster, of which perhaps a single TF may be the relevant regulator in the given condition. The TLC approach described here provides an objective statistical framework for evaluating the suitability of a proposed TF-to-target association based on a large set of time-course expression measurements. In particular, the approach enabled the preferential identification of TF-to-target associations for which the optimal time lag is biologically plausible, and the rejection of associations with a biologically implausible (e.g., zero) time lag. Four (TF,cluster) associations were validated using ChIP-on-chip assays, in which enriched binding of the relevant TF was shown among genes within the relevant cluster. The ChIP-on-chip enrichment *P* values are conservative estimates of the genome-wide binding enrichment, due to the fact that genes were selected for inclusion in the tiling array based on differential expression under LPS stimulation in a macrophage-like murine cell line (RAW 264.7). We note that for each of the two TFs assayed, two (TF,cluster) pairs were found to be enriched for binding based on ChIP-on-chip, but not based on the network analysis. Such false-negative predictions may be the result of binding sites sometimes occurring upstream of the 2 kbp region selected for TFBS motif scanning, the target TF being cross-linked to a DNA-bound co-regulator recognizing a different motif than the TF, or due to the TF recognizing a TFBS motif variant not represented in the motif database.

The inferred transcriptional network resulting from our analysis associates at least one TF with 27 of the 32 clusters. The 27 clusters comprise 86% of all differentially expressed genes, with an overall network coverage (including motif matches for individual targets) of 63%. An average of 3.3 TF genes were associated with each cluster, which may reflect the prevalence of combinatorial control in the transcriptional network. The TFs implicated in the network are also highly interconnected at the level of protein-protein interactions, and interacting TFs are found to co-associate with clusters in the network. Many TFs known to play a role in macrophage activation were strongly associated with clusters in the inferred network (e.g., NFκB, AP1, IRF family members, and STAT1). NFκB and AP1 appear to be the most prolific activators in the network. EGR family members appear to be associated with early-induced clusters, and IRF family members are associated with later-induced clusters. In particular, the network associated specific TFs with immunologically important gene clusters (e.g., EGR1/2 and AP1 regulating cluster C27; and NFκB and IRF1 regulating cluster C13). Finally, incorporating expression data enabled identifying a specific TF from among members of a large TF family recognizing a motif enriched within a target cluster; for example, the predicted interaction between IRF1 and C25 was validated by ChIP-on-chip. However, we note that more ChIP-on-chip data, with a variety of TF targets, would be required to quantitatively assess the performance of the combined network analysis compared to single-evidence analysis using sequence data or expression data alone.

We note that by including in the analysis only TFBS motifs for which at least one associated TF gene was differentially expressed, the inferred network does not include TFs for which there is *no* transcript-level differential expression; this trade-off enabled network inference based on *dual* criteria of motif match enrichment and the estimated time lag prior probability. Work is in progress to extend the analysis to include all 208 TFBS motifs corresponding to TFs that are transcriptionally expressed in the TLR-stimulated macrophage. Another limitation related to sequence scanning is that the promoter sequence data set used is purely upstream of the annotated transcription start site (TSS); recent evidence suggests that some TFs may be equally likely localized downstream of the annotated TSS [76]. In future work, it could be productive to scan for TFBS motifs both upstream and downstream of the annotated TSS.

In addition to recapitulating known regulators, the analysis identified a potential transcriptional regulator not previously known to play a direct role in TLR-stimulated macrophage activation, TGIF1. TGIF1 is a three-amino acid loop extension homeobox protein that acts as an obligate repressor through either direct binding to the retinoic acid responsive element on DNA, or through its interaction with SMAD2 in the TGFβ pathway [71]. Its associated TFBS motif is enriched within the promoters of genes within cluster C26 (*P*<10^−2^) and cluster C4 (*P*<10^−2^), and *Tgif1* is strongly (11-fold) upregulated in murine macrophages in response to *Streptococcus pyogenes* infection [77]. Particularly intriguing is the possibility that, in light of motif scanning evidence, TGIF1 may act as a transcriptional repressor of the cytokines *Csf2* and *Gm1960.*

The approach of combining promoter scanning-based evidence with expression dynamics-based evidence enabled more specific identification of the TF gene(s) regulating a cluster than would have been possible using promoter scanning alone. Time-course expression data allowed, in some cases, the disambiguation of which TF gene (out of a family of TF genes associated with a given TFBS motif) is the likely regulator of a cluster enriched for the corresponding TFBS motif. Inclusion of expression data provided a second source of evidence to indicate the relevance of a given TF gene for predicting the condition- and time-specific expression of a target gene cluster. In total, these results validate the strategy of computationally integrating two distinct large-scale data sources (expression and genomic sequence) to infer a murine macrophage transcriptional network. In a future study, additional sequence-based data sources, such as evolutionarily conserved elements in the *cis*-regulatory region, could be incorporated into the method.

## Materials and Methods

All data were analyzed in MatLab (MathWorks, Natick, MA) unless otherwise stated. In all cases where Fisher's exact test was performed, the test was one-tailed, using the cumulative distribution function (CDF) of the hypergeometric distribution.

### Microarray expression measurements

Mutant strains (see Table S1) were generated in the 129SVJ background and backcrossed to C57BL/6 (Jackson Laboratories), ten times. Femurs from the C57BL/6 and mutant strains were flushed with complete RPMI (RPMI 1640 supplemented with 10% FBS, 2mM L-glutamine, 100 IU/mL penicillin and 100 µg/mL streptomycin, all from Cellgro, Mediatech, except the FBS which was from Hyclone). Bone marrow cells were plated on non-tissue culture treated plastic in complete RPMI supplemented with recombinant human M-CSF (rhM-CSF) at 50 ng/mL (gift from Chiron). On day 4 the cells were washed two times with RPMI with no additions and then grown 2 more days in complete RPMI supplemented with 50 ng/mL of rhM-CSF. On day 6 the cells were lifted from the non-tissue culture treated plastic, counted and plated at a density of 1.04×10^5^ cells/cm^2^ (1×10^6^ cells per well in a 6-well dish) on tissue culture-treated plastic. On day 7 cells were stimulated with TLR agonists at the concentrations indicated in Table S2, without changing the media. Stimulus reagent sources are shown in Table S15. Stimulation of the cells was verified by the presence of TNFα in the culture supernatants detected by ELISA (Duoset ELISA Assay Development System, R&D Systems). Total RNA was isolated using TRIzol (Invitrogen) and analyzed for overall quality using an Agilent 2100 Bioanalyzer. mRNA was labeled using the Affymetrix One-Cycle Target Labeling protocol and reagents for eukaryotic target preparation. The labeled cRNA was hybridized to an Affymetrix GeneChip Mouse Genome 430 2.0 array using standard protocols and reagents from Affymetrix. Probe intensities were measured using the Affymetrix GeneChip Scanner 3000 and processed into CEL files using Affymetrix GeneChip Operating Software.

### Microarray data processing

Expression data were acquired from 216 microarray hybridization experiments comprising 95 combinations of strain, stimulus, and time point (hereafter, “experiments”; see Table S3), of which 41 were in mutant strains, and 54 in wild-type. Data in the form of CEL files were background-subtracted and normalized with the Robust Multi-chip Average (RMA) method [78] using the software Bioconductor [79], then exported to MatLab for further analysis. For each of the 95 experiments, normalized expression measurements for each probeset were averaged across biological replicates using the log_2_ intensities [78] to obtain the replicate-combined probeset intensity.

### Differential expression testing

Significance testing was performed using mean log_2_ intensities from 7 wild-type TLR-stimulation time-course experiments comprising 54 assays (where “assays” refers to a specific combination of strain, stimulus, and elapsed time; see Table S3) for which at least two replicates were available, relative to the mean log_2_ intensities of unstimulated wild-type macrophages (hereafter, the “reference experiment”). For each probeset and for each of the wild-type TLR-stimulation time-course experiments, a differential expression test was performed using a spline-based multivariate regression method [32] to obtain a *P* value for the difference in the sum-squared residuals under the alternative and the null hypotheses. A fourth-order polynomial basis was used, with 1,000 iterations for the bootstrap resampling. For each time-course experiment, a separate *P* value threshold was selected based on a maximum Benjamini-Hochberg false discovery rate (FDR) [80] as described below.

### Probeset selection

A probeset selection algorithm was carried out to select a representative probeset for each gene, eliminating probesets that are annotated as cross-hybridizing to transcripts from different genes.

Representative probesets from among the 45,037 probesets (excluding on-chip control probesets) on the Affymetrix Mouse GeneChip 430.2 were selected based on four criteria. A probeset was selected if and only if: (i) it possessed an Entrez GeneID in the Affymetrix probeset annotation database [81]; (ii) it had a log_2_ intensity exceeding a fixed cutoff, in at least one replicate-combined experiment; (iii) it had a *P* value less than a fixed cutoff, for at least one experiment; and (iv) its probeset name did not contain “_x_” or “_s_”, and was not associated (by GeneID annotation) with transcripts of two distinct genes. Criterion (iv) was imposed in order to eliminate probesets containing probes that cross-hybridize to transcripts from different genes [82]. Whenever multiple probesets mapped to the same GeneID (or the same collection of GeneIDs), the probeset with the smallest minimum *P* value, across all non-reference experiments, was selected as the “representative probeset” for the GeneID (or GeneID list).

This selection procedure was applied with four different cutoffs for log_2_ intensity and *P* value, as summarized in Table S16. (i) To generate a set of differentially expressed genes suitable for expression clustering (hereafter, the “target” genes), a log_2_ intensity cutoff of 6 was used, and a *P* value cutoff of 10^−4^ was used. The resulting number of representative probesets for target genes was 1,960. The complete list of the 1,960 target genes, and their expression measurements, are provided in Table S4. (ii) To generate a set of differentially expressed TF genes, the algorithm was re-run for probesets that are annotated as TFs, and for which a TRANSFAC matrix is available (see Selection of Transcription Factors), with a FDR cutoff of 0.05. A total of 80 differentially expressed TF genes were identified, as described in Selection of Transcription Factors below. (iii) To generate a set of all genes that were expressed in the macrophage, in at least one experiment, the probeset selection was run with a log_2_ intensity cutoff of 6 and no filtering for differential expression. The 8,788 resulting genes were used as the reference set for applying Fisher's exact test to the promoter scanning results (see Promoter Scanning below). (iv) To generate the set of all genes represented by “_at” or “_a_at” probesets on the GeneChip, the algorithm was run with no filtering for minimum intensity or differential expression. This generated a list of 20,905 genes that constituted the genome-wide set used in the gene ontology enrichment analysis (see Functional Enrichment Analysis below).

### Selection of transcription factors

A set of 388 position-weight matrices (PWMs) corresponding to murine TFs was obtained from the TRANSFAC Professional database version 10.3 [33]. These PWMs were mapped using TRANSFAC as well as literature searching, to 273 mouse genes that code for corresponding TFs or TF components. Of these, 80 TF genes were identified as differentially expressed (FDR≤0.05) as described in Probeset Selection above (see Table S5). The 80 TF genes are represented by 150 TRANSFAC position-weight matrices. Table S10 contains the microarray expression measurements for these TF genes.

To estimate the fraction of genes in the mouse genome that are TFs, a genome-wide list of 1,245 murine TF genes (and probable TF genes) was assembled by mapping a list of 1,800 human TF genes from the literature [83] to mouse orthologs present on the Mouse GeneChip and integrating the set of genes possessing GO annotations for transcription factor activity (GO:0003700).

### Expression clustering

The SDR values *x_pj_* for log_2_ intensity, where *p* indicates the probeset and *j* indicates the experiment, were clustered using a fast implementation of the *K*-means algorithm [84], with a minimum cluster size of 1. The number of clusters *K* was chosen to minimize the Bayesian Information Criterion (BIC) [38]. The BIC is a function of *K* represented as *BIC*(*K*),
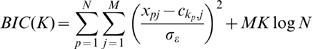
(9)where *k_p_* is the cluster to which the *p* ^th^ probeset is assigned, 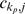 is the *j* ^th^ coordinate of the centroid of the *k* ^th^ cluster in the SDR-transformed space of expression measurements, *N* = 1,960 (the number of target genes), *M* = 94 (the number of non-reference experiments), and *σ_ε_^2^* is the average intra-cluster variance evaluated at *K* = 3. The *K*-means clustering was carried out for integer values 18≤*K*≤50, for 1,000 iterations at each value of *K*; the optimal clustering (lowest average BIC over the 1,000 iterations) occurred at *K* = 32 (see Figure S1). The cluster expression profiles were characterized using the within-cluster median of the SDR; as a result, the *cluster* expression profile will not necessarily have a maximum value of 1 across all data points. This is because, in general, the genes within a cluster will not all reach a maximum value at the same time point. The induction time scale for the median SDR expression within each cluster was estimated using linear interpolation between the time points for the wild-type LPS time-course, and finding the time at which the absolute value of the SDR first exceeded 0.25. Clusters were displayed (in Figure 1 and Figure S2) in the cluster order that minimized the sum of Euclidean distances between adjacent clusters, obtained using simulated annealing [85] with 5000 iterations and a cooling rate of 0.5. The cluster expression profiles in Figure 2 were ordered for display using hierarchical agglomerative linkage using the Euclidean distance of extremal SDR expression level in time-course microarray experiments under the four indicated TLR agonists.

### Functional enrichment analysis

Jackson Laboratory Mouse Genome Informatics GO annotations [86] were added to the Affymetrix Mouse GeneChip GO annotations [81] by string matching on the gene symbol field for each annotated probeset. For each of the 20,945 GO term IDs [87], the number of occurrences of the GO term ID (or a descendent of the GO term ID) in the GO hierarchy was computed for all 20,905 genes represented on the Affymetrix Mouse GeneChip (see Probeset Selection above) as well as for each co-expressed gene cluster. For each GO hierarchy (process, component, and function) the total number of genes possessing at least one GO annotation for the hierarchy was computed (see Table S17). The *P* value for GO enrichment was computed for each pair (*i*,*C*) of a GO term ID *i* and gene cluster *C*, using Fisher's exact test (under-occurrences of a GO term relative to the reference set were discarded). Any pairs (*i*,*C*) in which less than 5% of the genes within *C* possess GO term ID *i*, or with a term level in the GO hierarchy less than 3, were discarded. The resulting 629 (*i*,*C*) pairs were ordered by *P* value, and a *P* value cutoff was selected by demanding that the estimated false discovery rate be 0.02 (*P*≤0.0148, or −log_10_ *P*≥1.83). The resulting 460 GO term enrichments are shown in Table S8.

The list of 32 TLR-regulated murine cytokines was obtained by screening for all differentially expressed genes possessing an annotation for cytokine or chemokine activity, and by refining the list by using NCBI PubMed searches to determine whether each gene is a cytokine.

### Selection of genes for null distribution

To form the null distribution of time-lagged correlation, a set of non-TF genes was generated. From the set of 1,960 differentially expressed genes, a set *Q* of 484 genes were selected such that each gene: (i) does not correspond to a TRANSFAC transcription factor as described above; (ii) has at least two GO process and two GO function annotations; (iii) is not annotated as “regulation of transcription, DNA-dependent” (GO:0008015); (iv) does not have a gene name with the prefix “Zfp” (zinc finger protein); and (v) is not listed among the 1800 TF genes (see Selection of Transcription Factors). The time-lagged correlations between genes within this group were taken as the null distributions of time-lagged correlations, for the purpose of computing the *P* value of a time-lagged correlation between a TF and a gene (see Time-lagged Correlation below).

### Constructing the prior distribution of time lags

Given the time resolution of the expression data (for which the smallest Δ*t* is 20 min), the set *L* of time lags was chosen to be 0–80 min (inclusive), at 10 min intervals. The precision at which the optimal time lag can be estimated, at |*ρ*_τ_|≥0.9, was determined to be ±5 min, based on simulated independent Gaussian noise added to the replicate-combined array data with standard deviation given by the measured replicate-standard deviation of the log_2_ intensity in each experiment. The upper limit of 80 min was selected to ensure that in each time-course with time points *T*, the target gene expression evaluated at time points {*t*+*τ*|*t*∈*T* and *t*+*τ*≤max(*T*)} would always be based on measurements from at least three time points. The conditional probability density *P*(*τ*_c_|*H̅*_0_) of the overall transcriptional time delay *τ*_c_, for true interacting TF–target gene pairs, was defined using the gamma distribution (see Text S1, Section 3). This probability density was integrated for bins of *τ*_c_ centered at the discrete time lags *τ*∈*L*, to obtain an estimate of the discrete probability for observing an optimal time lag, where Δ*τ* = 10 min. Using the distribution *P*(*τ*_c_|*H̅*_0_), the upper limit of 80 min for *τ* included approximately 97% of transcriptional delays.

### Time-lagged correlation

The time-lagged correlation (TLC) was computed for all possible triples (*f*,*g,τ*) of TF gene *f*, potential target gene *g*, and time lag *τ* ∈ *L*. There were 80 TFs and 1,960 target genes. The TLC was computed as follows, for a given (fixed) time lag *τ*. Let the vectors *X_T_*(*f*) and *X_T_*(*g*) represent the log_2_-transformed, SDR-normalized expression measurements for *f* and *g* in a time-course, where *T* is the set of time points, and let *t*_max_≡max(*T*). Let *T_τ_*≡{*t*∈*T*|*t*≤*t*_max_−*τ*}. Let 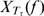 and 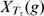 represent the measurements of *f* and *g,* respectively, at the times *T_τ_*. We now define the set of shifted time points *T'_τ_*≡*T_τ_*+*τ* = {*t*+*τ*|*t*∈*T_τ_*}. The expression values 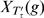 were computed using linear interpolation between the adjacent time points. Expression values 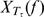 for each time course were concatenated together to obtain a combined multi-experiment vector 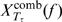 of measurements for *f* and a combined vector 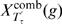 of time-boosted measurements for *g*. The TLC *ρ_τ_*(*f*,*g*) was then computed using Equation 2 and using 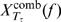 and 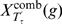. The criteria for inclusion of a time-course experiment in the TLC calculation were (i) a minimum of three points in the set *T_τ_*, and (ii) a minimum of three measurements contributing to the interpolated values 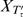. A total of eleven time-course experiments comprising 72 independent time points were included in the TLC analysis, as shown in Table S9.

To build the background (null) TLC distribution 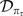 (as defined in Text S1, Section 2) for each time lag *τ*, the TLC was computed for a set *H* consisting of all non-identical pairs of genes (*h*_1_,*h*_2_), where *h*_1_ and *h*_2_ are drawn from the set *Q* of non-TF genes (see Selection of Genes for Null Distribution above). The background distributions were constructed from the *ρ_τ_* ^2^(*h*_1_,*h*_2_) values, using Gaussian kernel density estimation [38] (see also Text S1, Section 4) with a smoothing length of 0.005 (chosen to maximize the number of pair-wise associations in the non-background set for which *P* ^tlc^≤10^−3^). For each *τ* and each *ρ_τ_*(*f*,*g*), the complementary CDF 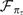 was computed by integration of 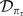 using the extended Simpson's Rule (closed interval) [85] with 200 bins.

The TLC was then analyzed for the set *G* of gene pairs (*g*_1_,*g*_2_), where *g*_1_ was drawn from the set of 80 TFs (see Selection of Transcription Factors above), *g*_2_ was drawn from the set of 1,960 differentially expressed (“target”) genes (see Probeset Selection above), and *g*_1_≠*g*_2_ (the inequality avoids perfect zero-time-lagged correlations that would bias the significance test). For each pair (*g*_1_,*g*_2_), the time lag that maximized 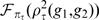 was selected as the optimal time lag for the pair, and denoted by *θ*(*g*_1_,*g*_2_).

The probability ratio *R*(*τ*) was computed using Equation 5. The marginal probability *P*(*H*_0_) was estimated to be ∼0.94 based on an analysis of the transcriptional network of [7], taking the average out-degree of the TFs in Fig. 4B and dividing by the number of differentially expressed genes in that study (1,784 genes). The marginal probability *P*(*τ*)was obtained from *θ*(*H*).

The combined, cumulative, TLC-based *P* value for (*f*,*g*), denoted by *P* ^tlc^(*f*,*g*), was computed according to Equation 7 (for which a detailed mathematical derivation is given in Text S1). Empirical evidence showing the approximate independence of *ξ* and *R* under the null hypothesis is shown in Figure S17. For each pair (*f*,*C*) of TF gene *f* and gene cluster *C* (see Expression Clustering above), an overall *F* score, *F* ^exp^ (*f*,*C*) was computed using Equation 8, combining the |*C*\{*f*}|*P* values. Because the genes within a cluster are grouped by expression similarity, their TLCs with respect to *f* are not independent, even under the null hypothesis that *f* does not regulate any of the genes within the cluster. Thus, among a large collection of pairs (*f*,*C*) satisfying the null hypothesis, the *F* scores *F* ^exp^ (*f*,*C*) will not be distributed according to the χ^2^ distribution with 2|*C*\{*f*}| degrees of freedom. Instead, the number of intra-cluster degrees of freedom was computed for each cluster by clustering the SDR expression profiles of the genes within a cluster (across all 94 non-reference experiments) using the *K*-means algorithm. For a range of numbers *k* of sub-clusters, the BIC was computed using the variance at *k* = 3 for normalizing the bias term [38]. The number of sub-clusters *k* at which the BIC was minimized was doubled to obtain the effective number of degrees of freedom, *d*(*C*), within each cluster. The average over all clusters was 〈*d*(*C_k_*)〉_k_ = 11.03, where *C_k_* denotes the *k* ^th^ cluster. The χ^2^ test was applied with *d*(*C*) degrees of freedom, to obtain an overall *P* value for the association between *f* and *C*:
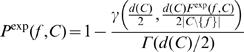
(10)where *F* ^exp^(*f*,*C*) is defined in Equation 8, and *γ* is the incomplete gamma function [85].

A second statistic, the average time lag, was computed for each pair (*f*,*C*),
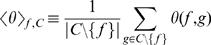
(11)and used as an additional criterion in the network inference (see Network Inference below).

### Promoter scanning

Mouse position-weight matrices (150 in total) corresponding to the 80 differentially expressed TF genes, were obtained from TRANSFAC Professional (see Selection of Transcription Factors above, and Table S5) [33]. Promoter sequences of 2 kbp upstream of 17,254 mouse genes were obtained from the UCSC genome database [47] (UCSC annotation build mm8, based on the NCBI mouse genome assembly m36), each identified by NCBI RefSeq ID. The [−2 kb, 0] coordinate range relative to the transcription start site was selected based on Figure 2c from [88]. For each representative probeset (see Probeset Selection above), the corresponding RefSeq ID (if available) was obtained from the Affymetrix GeneChip annotation file [81]. The 8,788 expressed genes mapped to 7,492 unique promoter sequences (hereafter, the “reference” set, denoted by *μ*_exp_ = 7,492). Of the 12,117 genes that were not expressed in any of the microarray experiments, 7,503 were mapped to UCSC promoter sequences (hereafter, the “background” set). The 1,960 differentially expressed genes were mapped to 1,713 unique promoter sequences. Low-complexity repeats were masked from all promoter sequences prior to motif scanning, using RepeatMasker [89]. Scanning was performed using MotifLocator version 3.2 [90], using a first-order background model with frequencies computed from the first 496 genes on chromosome 17, obtained from the 5 kbp upstream promoter sequence file from NCBI mouse genome assembly 32 (UCSC build mm4), and using motif matrix score thresholds selected as described below. The background sequences were scanned with all matrices with no cutoff. For each matrix, the score threshold was computed at which an above-threshold match would occur on average in one out of every 5 promoter sequences (i.e., once per 10 kb). The motif match score thresholds are given in Table S11. The reference promoter set was scanned using these score thresholds, and for each matrix *m*, the number of promoter sequences in the reference set that had at least one above-threshold match was denoted by *ν*_exp_ (*m*). For each cluster *C*, the mapped promoter sequences for the genes within the cluster (the number of which was denoted by *μ*(*C*)) were scanned, and the number of sequences with at least one above-threshold match was denoted by *ν*(*m,C*). For each matrix *m* and cluster *C*, a *P* value *P* ^scan^ (*m*,*C*) was computed from the values *μ*_exp_, *ν*_exp_ (*m*), *μ*(*C*), *ν*(*m,C*), using Fisher's exact test. Let Φ denote the mapping between the 80 TF genes and subsets of the 150 TRANSFAC matrices (see Table S5), so that Φ(*f*) is the set of TRANSFAC matrices associated with the TF gene *f*. For each TF gene *f* and cluster *C*, a *P* value representing the association between *f* and *C* was computed as follows,
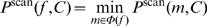
(12)The values of *μ*_exp_, *ν*_exp_ (*m*), *μ*(*C*), and *ν*(*m,C*) for all clusters, are provided in Table S11.

### Network inference

For each pair (*f*,*C*) of TF gene *f* and co-expressed gene cluster *C*, an overall combined *P* value, *P* ^comb^ (*f*, *C*) for the significance of the association between *f* and *C* based on both promoter scanning and expression time-course data, was computed using Fisher's method,
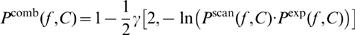
(13)The set of all pairs (*f*,*C*) were selected, satisfying the following criteria: (i) *P* ^comb^ (*f*, *C*)≤0.0248 (or −log_10_ *P* ^comb^ (*f*,*C*)≥1.61, where the *P* value cutoff was obtained using an FDR of 0.025); (ii) *P* ^scan^ (*f*,*C*)≤0.05 (or −log_10_ *P* ^scan^ (*f*,*C*)≥1.3); and (iii) 〈*θ*〉*_f,C_*≥10 min. Criterion (iii) was used to ensure that a pair (*f*,*C*) would not be accepted based solely on a very low *P* ^scan^ (*f*,*C*) value; the average optimal time lag must be biologically plausible. A total of three TF-cluster associations were rejected, that passed criteria (i) and (ii), but not criterion (iii). A total of 90 TF-cluster associations were identified based on these criteria, involving 36 TF genes. The out-degree of a TF gene *f* within the network was estimated by summing (over all clusters for which (*f*,*C*) was accepted) the product *z*(*f*,*C*) |*C*\{*f*}|, where *z*(*f*,*C*) is the fraction of genes within *C* that have at least one binding site for any matrix *m* ∈ Φ(*f*).

The diagrams shown in Figures S15 and S16 were generated using Cytoscape [91] version 2.5.0. Protein interactions were obtained from the Human Protein Reference Database [67], Release 6 (2007/01/01) and the Biomolecular Interaction Network Database [68] (2007/10/14). The 36 differentially expressed TF genes were mapped to human orthologs using NCBI Entrez Gene. For the protein network diagram shown in Figure S16, a minimum log_2_ microarray probeset intensity cutoff of 6.5 was required in at least one array experiment (with the exception of *Smad6,* whose human ortholog protein is expressed in HL60 macrophage differentiation [92]).

### Quantitative PCR

Total RNA was isolated from bone marrow-derived macrophages using TRIzol (Invitrogen), treated with DNAase (Ambion), and used as template for reverse transcription (Superscript II, Invitrogen) according to the manufacturers' instructions. qPCR was performed using Applied Biosystems ABI 7900 HT. Expression units were computed relative to the housekeeping gene *Eef1a1* [6],[93]. Primer reagents for *Tgif1* and *Eef1a1* were obtained as described in Table S15.

### ChIP-on-chip validation

Five (TF,cluster) pairs were selected for ChIP-on-chip validation based on several criteria: (1) the gene members of the cluster needed to be well-represented on the tiling array (at least 30% of the genes in the cluster must be represented on the ChIP-on-chip array); (2) a correlation between TF gene and cluster expression consistent with known function (activator or repressor) for the TF; (3) the availability of a high-quality polyclonal murine antibody for a relevant TF protein; (4) demonstrated specificity of the antibody based on Western blot analysis; (5) a successful ChIP assay for several known targets of the TF. Genome location was assayed using ChIP-on-chip hybridization as described in [6], with polyclonal antibodies for murine IRF1 and p50 (*Nfkb1*) (Table S15). A custom Affymetrix GeneChip microarray was used, consisting of 25-mer oligonucleotides selected to densely tile 20 kbp upstream and 20 kbp downstream (and selectively, the coding regions) of genes selected based on differential expression in preliminary microarray expression studies involving murine RAW 264.7 cells stimulated for 60 minutes by LPS, Pam_3_CSK_4_, or Pam_2_CSK_4_ [94]. Of the 1,960 differentially expressed genes identified in Probeset Selection, 517 are represented on the tiling array. Hybridization to the custom tiling array was carried out using standard protocols and reagents from Affymetrix. ChIP-on-chip microarray scans were background-adjusted and quantile normalized as described in [6]. ChIP-on-chip data were processed as follows. First, probes were sorted based on chromosomal location. The sample/control absolute intensity ratio was computed for each probe, where the control intensity was taken from an experiment with antibody, but without LPS stimulation. A smoothed intensity profile was then generated using a sliding window algorithm based on Tukey's biweight kernel [95] with a 100 bp window size (as was used in [6]). Probes were then selected for which the intensity ratio was higher than a statistical cutoff (*P*≤0.01). If there were multiple significant probes within a 200 bp region, the combined statistical significance of region was computed by performing a *t*-test in which the distribution of probe intensities within the 200 bp region is compared to a background region of probe intensities. For each identified chromosomal region, the annotated gene nearest to the region in the 5′ direction was recorded, along with the distance to the nearest flanking gene. Significance testing of the enrichment of ChIP-on-chip binding among genes within a specific cluster was carried out using Fisher's exact test with a background set consisting of all 520 differentially expressed mouse genes (see Differential Expression Testing above) for which at least one probe on the array is located within 20 kbp upstream of the TSS.

### Accession numbers

All microarray expression data from this study have been deposited into the ArrayExpress [96] public database under accession number E-TABM-310. NCBI Entrez Gene identifiers can be found for all differentially expressed genes considered in this study, in Tables S4 and S5. Mouse Genome Informatics Allele accession numbers are provided for each mutant strain, in Table S15.

## Supporting Information

Text S1 Mathematical Derivations. This document provides a complete mathematical description of the significance test used for the time-lagged correlation. In addition, it provides background information on the Gaussian kernel density estimation method and some key theorems supporting the derivation of the method.
(0.23 MB PDF)

Figure S1 The optimal number of clusters was determined using the Bayesian Information Criterion (BIC). The horizontal axis indicates the number of clusters *K* used for *K*-means clustering. The cluster analysis was repeated for *K* varying between 18 and 50, with the BIC computed for each number of clusters. The optimal number of clusters, for which the BIC is minimized, was found to be *K* = 32 (see Materials and Methods, Expression Clustering).
(0.15 MB TIF)

Figure S2 Differential expression profiles of gene clusters, in TLR-stimulated macrophages, across all microarray expression experiments. Each row represents an experiment (a specific combination of strain, stimulus, and time point), and each column represents a cluster. Clusters are displayed in the order that minimizes the sum of pairwise distances between adjacent clusters (see Materials and Methods, Expression Clustering). Each colored rectangle within the heat-map indicates the centroid of the expression levels for genes within the indicated cluster, for the indicated experiment. The differential expression level (SDR, see Equation 1) is indicated in red/green color, and varies between -1 (bright green) and 1 (bright red), with 0 (black) indicating no change from the expression level in the unstimulated wild-type macrophage. The shaded light gray/charcoal regions in the far left column indicate the genotype. The color-coding in the second-to-left column indicates the stimulus (color code legend in lower right; and see Table S2 for the concentrations). The four-digit numbers to the right of the color-code column, indicate the elapsed time (min) post-stimulation, for each experiment.
(1.38 MB TIF)

Figure S3 Cluster-median differential expression profiles in wild-type macrophages stimulated with LPS show a diversity of time scales. Each data point shown is the median of the SDR-transformed (see Equation 1) differential expression levels of the genes within the indicated cluster, at the indicated time after stimulation.
(0.34 MB TIF)

Figure S4 Cluster-median differential expression profiles in wild-type macrophages stimulated with Pam_3_CSK_4_ show a diversity of time scales. Each data point shown is the median of the SDR-transformed (see Equation 1) differential expression levels of the genes within the indicated cluster, at the indicated time after stimulation. Cluster C26 shows sustained activation under this stimulus, as opposed to the case of stimulation with LPS (see Figure S3).
(0.32 MB TIF)

Figure S5 Cluster-median differential expression profiles in wild-type macrophages stimulated with poly I:C show a diversity of time scales. Each data point shown is the median of the SDR-transformed (see Equation 1) differential expression levels of the genes within the indicated cluster, at the indicated time after stimulation. The core response Clusters C27 and C28 induce later in this time-course experiment than in the case of stimulation with LPS (Figure S3).
(0.32 MB TIF)

Figure S6 Cluster-median differential expression profiles of wild-type macrophages stimulated with R848 show a diversity of time scales. Each data point shown is the median of the SDR-transformed (see Equation 1) differential expression levels of the genes within the indicated cluster, at the indicated time after stimulation. Cluster C26 shows sustained activation under this stimulus, as opposed to the case of stimulation with LPS (see Figure S3).
(0.34 MB TIF)

Figure S7 Discretized prior probability distribution *P*(τ|*H0*) of observing an optimal time-lag τ, for a gene pair that have a transcriptional regulatory interaction. Here, the symbol ∼*H0* denotes the complement of the null hypothesis, i.e., that there is a transcriptional regulatory interaction (this is denoted by an overbar in the main text and in the supporting text). The symbol τ denotes the optimal time lag. For a discussion and derivation of the prior probability distribution of transcriptional time lags, see Materials and Methods (Constructing the Prior Distribution of Time Lags) and Text S1 (Section 3).
(0.26 MB TIF)

Figure S8 Histogram of time lag values that maximize the absolute time-lagged correlation coefficient, for randomly drawn pairs of non-transcription factor genes. The non-uniformity of the histogram (the highest counts appear at high and low values of the time lag) shows the inherent bias in the standard method of selecting the optimal time lag, i.e., maximizing the absolute lagged correlation coefficient. Time-lagged correlations could not be reliably estimated for time lags greater than 80 min, due to limited effective sample size for higher time lags (see Materials and Methods, Constructing the Prior Distribution of Time Lags).
(0.22 MB TIF)

Figure S9 Differential expression levels (SDR, see Equation 1) in wild-type macrophages stimulated with LPS, for 38 pairs of transcription factor genes and gene clusters. The pairs all show high-significance time-lagged correlation based on the significance criterion *P* ^exp^ ≤ 5×10^-3^, and all satisfy the minimum average time lag criterion <*θ*> ≥ 10 min. Differential expression levels are relative to wild-type unstimulated macrophages, with positive/negative values indicating upregulation/downregulation. The names of the TF gene and the correlated cluster are shown above each plot. The cluster expression level, shown in green, is the centroid from the *K* -means clustering algorithm (see Materials and Methods, Expression Clustering). Of the pairs, 23 have a positive time-lagged correlation coefficient, and 15 have a negative time-lagged correlation coefficient.
(0.52 MB TIF)

Figure S10 Combined plot showing (i) the histogram of -log_10_ *P* ^exp^ values for the significance of the time-lagged correlation; and (ii) the estimated false discovery rate, as a function of the -log_10_ *P* ^exp^ value. The *P* ^exp^ values were computed for all possible pairs of (*f*,*C*) of transcription factor gene *f* and coexpressed gene cluster *C*. The histogram was generated using 40 bins.
(0.27 MB TIF)

Figure S11 Histogram of positions of transcription factor binding site motif matches relative to transcription start site. The median distance from the transcription start site is 537 bp. The density of motif matches can be seen to peak at −20 bp relative to the start site.
(0.25 MB TIF)

Figure S12 Combined plot showing (i) the histogram of -log_10_ *P* ^scan^ values for enrichment of TFBS motifs within co-expressed gene clusters; and (ii) the estimated false discovery rate as a function of the -log_10_ *P* ^scan^ value. The *P* ^scan^ values were computed for all possible pairs pairs (*f*,*C*) of transcription factor gene *f* and cluster *C*, using the position-weight matrix associated with *f* that had the smallest enrichment *P* value for the promoters of the genes in cluster *C*. The histogram was generated using 40 bins.
(0.28 MB TIF)

Figure S13 Integrating the two sources of evidence using Fisher's method. Each blue circle represents a unique (TF,cluster) pair. The solid line indicates the cutoff for the combined *P* value, at FDR = 0.1. Data points to the lower left of the line have a *P* ^comb^ value smaller than the cutoff (see Materials and Methods, Network Inference). The dotted green line indicates the cutoff for the promoter scanning-based *P* value, *P* ^scan^ = 0.05. Pairs that fall below the green dotted line and to the lower-left of the solid magenta line and for which the average time lag <*θ*> ≥ 10 min, were included in the final network.
(0.59 MB TIF)

Figure S14 The set of transcription factor genes has a 20-fold variation in out-degree (number of target genes), within the transcriptional network. (a) Estimated out degree of transcription factor genes. The out degree of a transcription factor gene is the number of genes estimated to be regulated by the transcription factor(s) associated with that TF gene (i.e., of which that TF gene is a component). For each gene cluster with which a TF gene was associated, the number of genes within the cluster for which a motif match was found (corresponding to the TF gene), was tabulated. The number of target genes was summed over all clusters with which the TF was associated, based on the combined expression and promoter scanning data (see Materials and Methods, Network Inference). Among the 36 TF genes in the network, the estimated out degree had a median of 49, and a maximum value of 285. (b) Estimated significance of the association of the TF gene in the network. For each TF gene *f* implicated in the network, the minimum *P* value *P* ^comb^(*f*,*C*) of association with any cluster *C*, was used as a measure of the overall significance of the association of TF gene in the transcriptional network. Transcription factor genes are displayed in decreasing order of estimated out degree (number of target genes). Transcription factors associated with larger clusters are seen to correlate with higher significances in the network, as a consequence of the sample size-dependence of the statistical tests used for the motif scanning and expression dynamics evidences.
(0.51 MB TIF)

Figure S15 Transcription factors involved in macrophage activation are highly interconnected in the protein interaction network, and the interacting TFs co-associate with clusters. Nodes indicate TF genes whose transcript levels are differentially expressed in LPS-stimulated macrophages, and that are associated with the transcriptional network through the combination of scanning- and expression-based evidences. Node labels are gene names. A red node indicates upregulated gene expression under LPS, and green indicates downregulation, and a purple node indicates transient up- and downregulation. A blue arc indicates that the human orthologs of the murine proteins associated with the murine TF genes connected by the arc, have an interaction in the Human Protein Reference Database [68] or in the Biomolecular Interaction Network Database [69]. A thick black arc indicates that the two connected TF genes co-associate with one or more clusters within the network, *and* share a protein interaction (suggesting a possible transcriptional complex). A purple arrow indicates a known protein-DNA interaction between the source node's human ortholog protein and the promoter of the human ortholog of the gene indicated by the target node. Brown ellipses denote the core transcription factor complexes NFκB and AP1.
(0.64 MB TIF)

Figure S16 TGIF1 interacts with many members of the SMAD/AP-1 transcription complex. Shown here is a network diagram of 16 proteins that interact with the SMAD family of transcription factors SMAD1/2/3/6, the histone deacetylaces HDAC1/2, and the TG-interacting factors TGIF1/2. Nodes indicate proteins, and a blue line between two nodes indicates that the human orthologs of the two proteins have an interaction, in either the Human Protein Reference Database (HPRD) [68] or in the literature [72],[75]. Red arrows indicate human protein-DNA interactions annotated in the TRANSFAC database [34]. The diagram includes nearest-neighbors of the SMAD, HDAC, and TGIF families in the protein interaction network. Each node shown in the diagram corresponds to a transcript that is likely expressed in murine bone marrow-derived macrophages, based on having an above-threshold microarray intensity within at least one experiment (see Materials and Methods, Probeset Selection).
(2.02 MB TIF)

Figure S17 Histogram of the cumulative density function of *ω*, for the *ω* values for all sample points with *ψ* = 80 min. Strict uniformity of this distribution (for each and every outcome *ψ* = *τεL*) would imply that ω is totally independent of *ω*|*ψ*. Here, conditioning on *ψ* is seen to not introduce a significant bias in the distribution of *ω* values (see Supporting Text, Section 2).
(0.30 MB TIF)

Table S1 Summary of mutant mouse strains used in this study. Expression data from available mouse strains with mutations of known TLR signaling adapter molecules or known transcriptional regulators were included in the cluster analysis, in order to maximize the diversity of expression patterns in the data set used for clustering. Column 1 is the mutant strain name. Column 2 is the name of the molecule affected by the mutation. Column 3 gives the gene title. Column 4 briefly summarizes the relevance of the molecule in TLR-stimulated macrophages.
(0.03 MB DOC)

Table S2 Stimuli used for macrophage gene expression experiments. Column 1 indicates the purified TLR agonist. Column 2 gives the description of the agonist. Column 3 indicates the receptor(s) that are stimulated by the agonist. Column 4 indicates the adapter molecule(s) associated with the receptor. Column 5 indicates the concentration used for *in vitro* stimulation of macrophages.
(0.04 MB DOC)

Table S3 List of microarray experiments included in this study. Each row indicates a microarray experiment. Column 1 indicates the mouse strain, with “Wild-type” indicating C57BL/6. Column 2 indicates the stimulus (or combination of stimuli, separated by a slash “/”). Column 3 indicates the elapsed time post stimulation. Column 4 indicates the number of biological replicates combined in the experiment. Column 5 indicates whether the expression measurements for the experiment were used in identifying differentially expressed genes. Column 6 indicates if the experiment was used for the clustering analysis. Column 7 indicates if the experiment was used for time-lagged correlation (TLC) analysis. The alternating shaded pattern for rows is used to visually distinguish between experiments from different genotypes.
(0.21 MB DOC)

Table S4 Target genes with microarray expression data. This spreadsheet contains the replicate-combined probeset intensities for all 1,960 differentially expressed genes (see Materials and Methods, Probeset Selection) across all 95 microarray experiments (see Table S3). Column 1 indicates the NCBI gene symbol of the gene. Column 2 indicates the NCBI Entrez Gene ID. Column 3 indicates the probeset selected as representative for the gene. Column 4 provides a brief gene description, obtained from the Affymetrix Mouse GeneChip annotations file. Column 5 indicates the co-expressed gene cluster to which the gene was assigned (see Materials and Methods, Expression Clustering). Columns 6–8 provide listings of the gene's Gene Ontology annotations in the process, component, and function GO hierarchies, respectively (see Materials and Methods, Functional Enrichment Analysis). Column 9 indicates the maximum log_2_ intensity observed, across all experiments. Columns 10-104 provide the log_2_ intensity measurements of the probesets across all 95 microarray experiments.
(4.30 MB XLS)

Table S5 Differentially expressed transcription factor genes considered as possible regulators of co-expressed gene clusters in this study. Column 1 contains gene symbol. Column contains the NCBI Entrez GeneID for the gene. Column 3 contains the representative Affymetrix probeset selected for the gene. Column 4 contains the co-expressed gene cluster of which the transcription factor is a member. Column 5 contains the TRANSFAC position-weight matrices that are associated with the transcription factor (or TF component) coded for by this gene (see Materials and Methods, Selection of Transcription Factors). The “V$” prefixes on TRANSFAC matrices are not shown.
(0.13 MB DOC)

Table S6 Summary of co-expressed gene clusters. Column 1 indicates the cluster name. Clusters were numbered in order of decreasing size. Column 2 indicates the number of genes in the cluster. Column 3 is a heat-map representation of the within-cluster median of the normalized differential expression intensity (SDR, see Equation 1), over time, in wild-type macrophages stimulated with LPS. The color red indicates upregulation relative to wild-type unstimulated macrophages, and green indicates downregulation (see color bar in Figure S2). Column 4 indicates the cluster response time under LPS stimulation, defined as the time scale (in minutes) for the log_2_ fold change to reach 25% of its extremal value (see Materials and Methods, Expression Clustering); the time scale uncertainty is ± 5 min. Column 5 lists the known (excluding those solely inferred from electronic annotation, i.e., “IEA” evidence code) transcription factor genes that are *members* of the cluster (these are *not* the inferred transcriptional regulators of the cluster). Column 6 lists the known cytokines and chemokines that are members of the indicated cluster.
(0.13 MB DOC)

Table S7 The timing of induction of core response clusters C27 and C28 is adapter molecule-dependent. Column 1 indicates the stimulus. Column 2 indicates the microarray conditions compared, for example, fold-change (stimulated relative to unstimulated) in *Myd88* ^(−/−)^ macrophages vs. the fold-change in wild-type. Column 3 indicates the time post-stimulation. Columns 4 and 5 are the within-cluster medians of the log_2_ of the ratios for the condition comparison indicated in column 2, for the clusters C27 and C28, respectively. The data indicate that the early response of these clusters is largely dependent on the MyD88 signaling pathway, and that the later response (2 hours) is more strongly dependent on the TRIF signaling pathway.
(0.03 MB DOC)

Table S8 Gene Ontology enrichments in co-expressed gene clusters. Column 1 indicates the cluster. Column 2 contains the Gene Ontology ID (GOID) for the GO term. Column 3 contains the GO term. Column 4 indicates the GO hierarchy (process, component, or function) to which the GO term belongs. Column 5 contains the -log_10_ *P* value (significance) for the enrichment of the GO term in the indicated cluster. Column 6 contains the level of the GO term in the gene ontology hierarchy. Column 7 indicates the number of genes within the cluster that possess this GO term. Column 8 indicates the frequency at which this GO term appears in the set of all annotated genes in the genome (see Materials and Methods, Functional Enrichment Analysis). Column 8 indicates the frequency at which the GO term appears among genes in the indicated cluster.
(0.54 MB XLS)

Table S9 Time-course macrophage stimulation microarray experiments used for time-lagged correlation analysis. Only time-course expression studies with a sufficient number of time points to admit time-lagged correlation analysis are shown (see Materials and Methods, Time-lagged Correlation). Column 1 indicates the genotype from which macrophages were derived. Column 2 indicates the stimulus used. Column 3 indicates the times post-stimulation, at which gene expression was measured.
(0.04 MB DOC)

Table S10 Transcription factor genes with microarray expression data. This spreadsheet contains microarray probeset intensities for all 80 differentially expressed transcription factor genes (see Materials and Methods, Selection of Transcription Factors) across all 95 microarray experiments (see Table S3). Column 1 indicates the NCBI gene symbol of the gene. Column 2 indicates the NCBI Entrez Gene ID. Column 3 indicates the probeset selected as representative for the gene. Column 4 provides a brief gene description, obtained from the Affymetrix Mouse GeneChip annotations file. Column 5 indicates the co-expressed gene cluster to which the gene was assigned (see Materials and Methods, Expression Clustering). Columns 6–8 provide listings of the gene's Gene Ontology annotations in the process, component, and function GO hierarchies, respectively (see Materials and Methods, Functional Enrichment Analysis). Column 9 indicates the set of TRANSFAC matrices associated with this transcription factor gene (see Materials and Methods, Selection of Transcription Factors). Column 10 indicates the maximum log_2_ intensity observed, across all experiments. Columns 11–105 provide the log_2_ intensity measurements of the probesets, across all 95 microarray experiments.
(0.21 MB XLS)

Table S11 Transcription factor binding site (TFBS) motif position-weight matrices, threshold scores, and number of matches for promoter TFBS motif searching. This spreadsheet contains the results from scanning the promoters of all genes in the reference set and in each co-expressed cluster, for transcription factor binding site motifs from TRANSFAC (see Materials and Methods, Promoter Scanning). Column 1 contains the TRANSFAC matrix name. Column 2 contains the minimum MotifLocator match score required for the given PWM to be identified as matching the sequence at a given chromosomal location. Column 3 contains the number of matches within the set of 7,492 reference promoter sequences. Columns 4–35 contain the number of matches for the PWM for each of the 32 co-expressed gene clusters. Section 2 contains the *P* values of the enrichments of the PWM matches within each of the 32 clusters (see Materials and Methods, Promoter Scanning). Row 2 indicates the number of genes whose promoters were scanned, for each cluster. The number of matches for each motif within each of the clusters is shown in a second section of the spreadsheet, starting at row 154).
(0.15 MB XLS)

Table S12 Time-lagged correlation data for all (TF,target) gene pairs in which a motif associated with the TF gene was found to match within the promoter region of the target gene. Column 1 contains the transcription factor gene symbol. Column 2 contains the transcription factor gene's Affymetrix probeset ID. Column 3 contains the target gene symbol. Column 4 contains the target gene's Affymetrix probeset ID. Column 5 indicates the co-expressed gene cluster (1-32) of which the target gene is a member. Column 6 indicates the time-lagged correlation coefficient between the TF and the target genes, at the optimal time lag. Column 7 indicates the optimal time lag selected for the gene pair. Column 8 contains the score assigned to the motif match by MotifLocator.
(7.84 MB XLS)

Table S13 ChIP-on-chip data. Results of five ChIP-on-chip assays for predicted (TF,cluster) pairs. Each row in the table shows integrated data sources for a specific gene target. Column 1 indicates the TF gene predicted to regulate the target cluster. Column 2 gives the probeset of the TF gene. Column 3 indicates the gene symbol of the target gene. Column 4 gives the target gene probeset. Column 5 gives the co-expressed cluster of which the target gene is a member. Column 6 gives the score for the best motif match for the indicated TF, within the promoter of the target gene (a blank cell indicates that no above-threshold motif match was found, at the 1 match per 10 kbp level of stringency). Column 7 indicates the *P* ^tlc^ from time-lagged correlation. Column 8 indicates whether the gene's promoter region was represented on the promoter array. Column 9 indicates the ChIP-on-chip *P* value; a blank cell in this column indicates that no significant ChIP-on-chip binding was found (see Materials and Methods, ChIP-on-chip Validation).
(0.05 MB XLS)

Table S14 ChIP-on-chip enrichment results for co-expressed gene clusters that are well-represented on the promoter array. Each row in the table gives results for the ChIP-on-chip assay for a particular cluster and for a particular TF target. Each row in the table is associated with a particular cluster and a particular TF target, for all pairings of p50/NFKB1 and IRF1 with the nine clusters for which at least 30% of the member genes were represented on the tiling array. The first column indicates the TF target. The second column gives the cluster number. The third column gives the number of genes on the ChIP-on-chip array for which binding was observed upstream of the transcription start site. The fourth column gives the number of genes within the cluster, that were represented on the ChIP-on-chip array. The fifth column gives the number of genes within the cluster that showed evidence of TF binding in the upstream region, in the ChIP-on-chip assay. The sixth column gives the fraction of genes in the cluster that are represented on the array. The seventh column gives the enrichment *P* value for the ChIP-on-chip hits within the cluster (see Materials and Methods, ChIP-on-chip Validation). The eighth column gives the motif match enrichment *P* value based on sequence scanning (see Materials and Methods, Promoter Scanning). The ninth column gives the *P* value based on the time-lagged correlation of expression profiles of the TF gene and the genes within the target cluster. The tenth column gives the average time lag, between the TF gene and the genes within the target cluster. The eleventh column gives the combined *P* value based on motif match enrichment and time-lagged correlation (see Equation 13).
(0.02 MB XLS)

Table S15 List of key materials and reagents. Column 1 indicates the type of material (mouse strain or stimulus reagent). Column 2 indicates the specific strain or reagent. For mutant mouse strains, the Mouse Genome Informatics accession number of the allele is provided. Column 3 indicates the source laboratory from which the mouse strain or reagent was obtained.
(0.05 MB DOC)

Table S16 Summary of probeset selection criteria. Each row describes a set of data selection criteria, for a specific purpose. For a detailed explanation of each set of criteria, see Materials and Methods, Probeset Selection. Column 1 states the purpose of the set of selection criteria. Column 2 indicates the minimum log_2_ absolute probeset intensity that must have been recorded in at least one experiment, for the gene to be included in the selection described in Column 1. Column 3 indicates the false discovery rate used to determine the *P* value cutoffs for each of the seven time-course experiments used for differential expression testing (see Materials and Methods, Differential Expression Testing); “n/a” means that no differential expression test was applied, for genes in the indicated row. Column 4 gives the number of probesets resultant from the indicated selection criteria.
(0.03 MB DOC)

Table S17 The total numbers of genes that possess gene ontology (GO) annotations, from each GO term hierarchy. Representative genes are selected from the set of annotated Affymetrix probesets as described in Materials and Methods, Probeset Selection.
(7.84 MB XLS)
