# Correction: Uncovering a Macrophage Transcriptional Program by Integrating Evidence from Motif Scanning and Expression Dynamics

**DOI:** 10.1371/annotation/1c55be5f-ecd7-49be-91c1-91881be60297

**Published:** 2008-03-25

**Authors:** Stephen A. Ramsey, Sandy L. Klemm, Daniel E. Zak, Kathleen A. Kennedy, Vesteinn Thorsson, Bin Li, Mark Gilchrist, Elizabeth S. Gold, Carrie D. Johnson, Vladimir Litvak, Garnet Navarro, Jared C. Roach, Carrie M. Rosenberger, Alistair G. Rust, Natalya Yudkovsky, Alan Aderem, Ilya Shmulevich

The wrong file was inadvertently uploaded for Table S17. The authors regret this error. The file should contain the following table:

Criteria Number of genes

======================================

Total representative genes: 20,905

Have at least one GO annotation: 12,515

Have at least one GO process annotation: 8,058

Have at least one GO component annotation: 7,797

Have at least one GO function annotation: 10,170

The correct Supplementary Table file can be obtained at:
