# Correction: Uncovering a Macrophage Transcriptional Program by Integrating Evidence from Motif Scanning and Expression Dynamics

**DOI:** 10.1371/annotation/e14ad837-e5ff-4bd5-a5f2-f32e784d75a2

**Published:** 2008-04-02

**Authors:** Stephen A. Ramsey, Sandy L. Klemm, Daniel E. Zak, Kathleen A. Kennedy, Vesteinn Thorsson, Bin Li, Mark Gilchrist, Elizabeth S. Gold, Carrie D. Johnson, Vladimir Litvak, Garnet Navarro, Jared C. Roach, Carrie M. Rosenberger, Alistair G. Rust, Natalya Yudkovsky, Alan Aderem, Ilya Shmulevich

The following sentences were omitted from the Funding information: Additional support was provided by grant P50-GM076547 from the National Institute of General Medical Sciences. IS acknowledges support from the National Institutes of Health grant R01-GM072855.

