# Supplementary material for: Uncovering a Macrophage Transcriptional Program by Integrating Evidence from Motif Scanning and Expression Dynamics
Source: PLoS Comput Biol. 2008 Mar 21;4(3):e1000021. doi: 10.1371/journal.pcbi.1000021 (PMC2265556; doi:10.1371/journal.pcbi.1000021)
Supplement: Text S1 — Mathematical Derivations. This document provides a complete mathematical description of the significance test used for the time-lagged correlation. In addition, it provides background information on the Gaussian kernel density estimation method and some key theorems supporting the derivation of the method. (0.23 MB PDF) [file pcbi.1000021.s001.pdf]

Uncovering a macrophage transcriptional program by integrating evidence from motif scanning and expression dynamics

Supporting Text: Mathematical Derivations

Stephen A. Ramsey, Sandy L. Klemm, Daniel E. Zak, Kathleen A. Kennedy, Vesteinn Thorsson, Bin Li, Mark Gilchrist, Elizabeth Gold, Carrie D. Johnson, Vladimir Litvak, Garnet Navarro, Jared C. Roach, Carrie M. Rosenberger, Alistair G. Rust, Natalya Yudkovsky, Alan Aderem*, Ilya Shmulevich*

Institute for Systems Biology, Seattle, Washington, United States

December 7, 2007

1 Motivation

The goal of this section is to derive a mathematical measure by which a null hypothesis (denoted by H_0) can be evaluated for an arbitrary pair of differentially expressed genes (g_1, g_2), where g_1 is a transcription factor (TF) gene, and g_2 is a possible target gene. Here, H_0 asserts that there is no direct transcriptional regulatory interaction between g_1 and g_2 – i.e., the protein product of g_1 does not bind the promoter of g_2 as a TF or a TF component. This measure will take into account two different sources of evidence, the strength of the time-lagged correlation (TLC) between time-course measurements of the expression levels of the gene pair, and the time lag at which the time-lagged correlation coefficient (TLCC) is statistically most significant. In this document, the significance measure is first described procedurally in Sections 2–3. A self-contained presentation of all background material,

*To whom correspondence should be addressed. Email: aderem@systemsbiology.org, ishmulevich@systemsbiology.org

including formal definitions of the kernel density method and proofs of all Propositions, is given in the second half of this document (Sections 4–5).

2 Description of the method

Because the true set of all pairs of differentially expressed genes (g_1, g_2) satisfying the null hypothesis H_0 under TLR stimulation cannot be known, it is necessary to adopt a model for the set of pairs satisfying H_0, from which a background distribution of TLCCs can be estimated. In this work, we choose to model H_0 by using a set H of pairs of differentially expressed genes (h_1, h_2) for which the protein product of h_1 is not a transcription factor. Thus, each gene pair in H automatically satisfies H_0. A description of how the set H is compiled can be found in the main text (see Materials and Methods). Since both the individual genes making up of pairs within H and the set of differentially expressed transcription factor genes are distributed throughout the various co-expressed clusters (see Table S6), using the set H to model the set of all gene pairs for which H_0 holds, is likely a reasonable approximation. We denote by G the set of gene pairs for which the null hypothesis H_0 is to be evaluated, namely, the set of pairs (g_1, g_2) of genes for which g_1 is a differentially expressed transcription factor gene, and g_2 is a differentially expressed gene. Let L be the set of time lags at which the TLCC is to be computed, which in this work is the set $\{0, 10, 20, 30, 40, 50, 60, 70, 80\}$ min (see Materials and Methods).

For each $\tau \in L$, and for any gene pair $(h_1, h_2) \in H$, one can compute the squared TLCC, $\rho_\tau^2(h_1, h_2)$, using Equation 2 in the main text. A single TLCC is obtained representing the correlation across multiple time-course experiments (see Materials and Methods). It is convenient to represent the squared coefficient correlation as a family of functions $\pi_\tau : H \to [0, 1]$ defined by

$$\pi_\tau(h_1, h_2) \equiv \rho_\tau^2(h_1, h_2),$$

for all $(h_1, h_2) \in H$ and for each $\tau \in L$. For each time lag $\tau \in L$, we construct the histogram of values of $\pi_\tau(H)$. To the extent that the elements of H are representative of the set of all differentially expressed gene pairs satisfying H_0, the smooth probability density function (PDF) estimated from the values of $\pi_\tau(H)$ using kernel density estimation (see Section4.2) should approximate the continuous probability density function (PDF) for values of π_τ for randomly selected pairs of differentially expressed genes satisfying H_0. In this work, the Gaussian kernel method (formally defined in Definition 3) is used to obtain a continuous PDF $\mathcal{D}_{\boldsymbol{\pi}_\tau}$ estimating the

true distribution of TLCCs for gene pairs satisfying H_0. For notational simplicity, the dependence of $\mathcal{D}_{\boldsymbol{\pi}_\tau}$ on the smoothing length s used in the Gaussian kernel (see Materials and Methods) is not shown. The PDF $\mathcal{D}_{\boldsymbol{\pi}_\tau}$ is a function from $\mathbb{R}$ to $\mathbb{R}^+$ (the positive reals), but its values outside of the unit interval $[0,1]$ are exponentially suppressed in the limit of small s. By integrating each $\mathcal{D}_{\boldsymbol{\pi}_\tau}$ we obtain a cumulative distribution function (CDF) $\mathcal{F}_{\boldsymbol{\pi}_\tau}$, which is a smooth function from $\mathbb{R}$ to $[0,1]$. It can be shown that $\mathcal{F}_{\boldsymbol{\pi}_\tau}$ is a smooth function approximating the fractional rank of squared TLCC values $\pi_\tau(H)$ (see Proposition 1). As explained in Section 4.3, each of the $\mathcal{F}_{\boldsymbol{\pi}_\tau}$ functions (one for each $\tau \in L$) is continuous and strictly monotonic. For each $\tau \in L$ and for each $(h_1, h_2) \in H$ we compute (using numerical integration) a fractional rank of the score $\pi_\tau(h_1, h_2)$, which we denote by $\mu_\tau(h_1, h_2)$,

$$\mu_\tau(h_1, h_2) = \mathcal{F}_{\boldsymbol{\pi}_\tau}(\pi_\tau(h_1, h_2)). \tag{1}$$

Formally, each μ_τ is a function from H to $[0,1]$. The value $\mu_\tau(h_1, h_2)$ represents the fractional rank (obtained using the smoothed distribution $\mathcal{F}_{\boldsymbol{\pi}_\tau}$) of the squared TLCC $\rho_\tau^2(h_1, h_2)$ within the set of values $\rho_\tau^2(H)$. We are now ready to define the optimal time lag ψ, and an associated significance measure ω, in terms of $\{\mu_\tau\}_{\tau \in L}$. For each $(h_1, h_2) \in H$, we compute $\psi(h_1, h_2)$ and $\omega(h_1, h_2)$ as follows:

$$\psi(h_1, h_2) = \underset{\tau \in L}{\operatorname{argmax}} \left(\mu_\tau(h_1, h_2)\right), \tag{2}$$

$$\omega(h_1, h_2) = 1 - \max_{\tau \in L} \left(\mu_\tau(h_1, h_2)\right), \tag{3}$$

The value $\psi(h_1, h_2)$ represents the time lag at which the fractional rank is maximal, and the value $\omega(h_1, h_2)$ represents the complementary maximum (across time lags) fractional rank. Formally, ψ is a function from H to L, and ω is a function from H to $[0,1]$. We note in passing that the definition of ψ encodes the unbiased method of time lag selection. In the standard method of lag selection, one would define the optimal time lag ψ_{st} as

$$\psi_{\mathrm{st}}(h_1, h_2) = \underset{\tau \in L}{\operatorname{argmax}} \left(\pi_\tau(h_1, h_2)\right),$$

which would introduce a bias towards selecting a τ for which as few sample points as possible contribute to ρ_τ^2 (see the main text subsection, Expression Dynamics Analysis).

At this point, it is convenient to employ a theorem (see Proposition 2 and Definition 4 in Section 4.3) which says that given the continuous PDF $\mathcal{D}_{\boldsymbol{\pi}_\tau}$,

one can define a continuous random variable $\boldsymbol{\pi}_\tau$ whose probability density function is given by $\mathcal{D}_{\boldsymbol{\pi}_\tau}$ and whose cumulative distribution function (CDF) is given by $\mathcal{F}_{\boldsymbol{\pi}_\tau}$. The CDF $\mathcal{F}_{\boldsymbol{\pi}_\tau}$, in the limit of small s, reproduces the cumulative normalized histogram of $\pi_\tau(H)$ (see Proposition 3). In the same sense that $\mathcal{D}_{\boldsymbol{\pi}_\tau}$ approximately represents the histogram of TLCC values $\pi_\tau(H)$, the random variable $\boldsymbol{\pi}_\tau$ represents computing the TLCC for pairs of genes selected from H at random with uniform probability (see Section 4.2). Since a function of a random variable is itself a random variable (see Definition 2), the smoothed fractional rank of the random variable $\boldsymbol{\pi}_\tau$ is itself a continuous random variable denoted by $\boldsymbol{\mu}_\tau$,

$$\boldsymbol{\mu}_\tau \equiv \mathcal{F}_{\boldsymbol{\pi}_\tau}\left(\boldsymbol{\pi}_\tau\right), \tag{4}$$

for each $\tau \in L$. Recall that for a finite set of N measurements, the rank-transformed measurements are uniformly distributed between 1 and N. Similarly, the fractional rank-transformed TLCCs $\{\boldsymbol{\mu}_\tau\}$ are each uniformly distributed on the unit interval (see Proposition 8). This means that the family of random variables $\{\boldsymbol{\mu}_\tau\}$ are identically distributed; however, they are not (in general) independent.

In terms of the random variables $\boldsymbol{\mu}_\tau$, the the optimal time lag ψ and complementary maximum fractional rank ω become random variables $\boldsymbol{\omega}$ and $\boldsymbol{\psi}$,

$$\boldsymbol{\psi} \equiv \underset{\tau \in L}{\operatorname{argmax}}\left(\boldsymbol{\mu}_\tau\right), \tag{5}$$

$$\boldsymbol{\omega} \equiv 1 - \max_{\tau \in L}\left(\boldsymbol{\mu}_\tau\right). \tag{6}$$

The random variable $\boldsymbol{\psi}$ is discrete (with possible outcomes given by the set L), and $\boldsymbol{\omega}$ is continuous (with possible outcomes spanning the unit interval $[0, 1]$). We now briefly address the question of under what assumptions about $\mathcal{D}_{\boldsymbol{\pi}_\tau}$ will $\boldsymbol{\psi}$ and $\boldsymbol{\omega}$ be independent. For the set H of non-interacting gene pairs (i.e., ordered pairs of genes for which the first gene is not a direct transcriptional regulator of the second), to a good approximation the non-independence of the $\{\boldsymbol{\mu}_\tau\}_{\tau \in L}$ can be expressed in terms of a τ-independent bias $\boldsymbol{\alpha}$ that contributes additively to each $\boldsymbol{\mu}_\tau$. Under this assumption, $\boldsymbol{\psi}$ and $\boldsymbol{\omega}$ can be shown to be independent (see Proposition 9). Empirical evidence for the approximate independence of $\boldsymbol{\psi}$ and $\boldsymbol{\omega}$ for the set H used in this work, is shown in Figure S17.

Having described a method for selecting an optimal time lag ψ that is unbiased on the set H, we now propose a probabilistic method to account for the biological likelihood of the time lag, in the assessment of the significance

of the TLCC. Specifically, for a given outcome $\tau \in L$ for $\boldsymbol{\psi}$, we compute relative likelihood of the null hypothesis H_0, given the outcome τ for the optimal time lag. This relative likelihood $R(\tau)$ is defined by the equation

$$R(\tau) = \frac{P(H_0|\boldsymbol{\psi} = \tau)}{P(H_0)},$$

where $P(H_0)$ is the prior probability that for $(g_1, g_2) \in G$, (which means that g_1 a transcription factor gene), the pair satisfies H_0 (i.e., that g_1 does not directly regulate g_2). The probability $P(H_0)$ can be either estimated from prior knowledge of the transcriptional regulatory network, or treated as a tunable parameter that controls the strength of the τ-dependence of $R(\tau)$. Each value of $P(H_0|\psi = \tau)$, for $\tau \in L$, is the conditional probability of H_0 for an observed optimal time lag $\psi = \tau$. Formally, R is a function from L to $\mathbb{R}^+$. Using Bayes's Rule and the rule of total probability, the above expression for $R(\tau)$ can be written as

$$R(\tau) = \frac{1}{P(H_0)} \left(1 - \frac{(1 - P(H_0))\, P(\tau|\overline{H_0})}{P(\tau)}\right),$$

where $P(\tau)$ is the prior probability of a time lag τ, for a randomly selected pair of genes. It is straightforward to estimate $P(\tau)$ directly from the expression data set, by computing the frequency at which each time lag τ is found to be optimal according to Eq. 2 (see Materials and Methods). The function $P(\tau|\overline{H_0})$ is the conditional probability that, given a direct transcriptional regulatory interaction, the intrinsic transcriptional time delay (at which the TLC is most significant) will have the value τ. This function is given an explicit parametric form in Section 3. Given that $\boldsymbol{\psi}$ is independent of $\boldsymbol{\omega}$, it follows that $\boldsymbol{\omega}$ is independent of $R(\boldsymbol{\psi})$ (see Proposition 10). We note that for a biologically plausible time lag τ, $R(\tau)$ will take on a smaller value, and for a biologically implausible time lag, $R(\tau)$ will take on a larger value – this is because $R(\tau)$ is the relative likelihood of the *null* hypothesis, given the observed time lag.

We can now define our overall measure of significance for the TLCC. Based on the independence of $\boldsymbol{\omega}$ and $R(\boldsymbol{\psi})$, and in analogy with Fisher's method for combining P-values [1], let $\boldsymbol{\gamma}$ be the random variable defined by

$$\boldsymbol{\gamma} \equiv \ln\left(\boldsymbol{\omega} R(\boldsymbol{\psi})\right).$$

The formal construction for interpreting a function of a random variable as a random variable, is given in Definition 2. The variable $\boldsymbol{\gamma}$ is continuous

and takes values on $\mathbb{R}$. We compute the value of γ for all $(h_1, h_2) \in H$,

$$\gamma(h_1, h_2) = \ln\left(\omega(h_1, h_2) R(\psi(h_1, h_2))\right).$$

The kernel density estimation procedure is then used to obtain the PDF $\mathcal{D}_{\boldsymbol{\gamma}}$ for all values $\gamma(H)$ (see Definition 3). The CDF $\mathcal{F}_{\boldsymbol{\gamma}}$ is then obtained from $\mathcal{D}_{\boldsymbol{\gamma}}$ by integration. Formally, $\mathcal{D}_{\boldsymbol{\gamma}}$ is a function from $\mathbb{R}$ to $\mathbb{R}^+$, and $\mathcal{F}_{\boldsymbol{\gamma}}$ is a function from $\mathbb{R}$ to $[0, 1]$. By Definition 2, the composition $\mathcal{F}_{\boldsymbol{\gamma}}(\boldsymbol{\gamma})$ is a random variable, and by Proposition 8, it is uniformly distributed on $[0, 1]$. Since it is uniformly distributed on the unit interval, and since a high-significance TLCC with optimal time lag will result in a very small value for γ and thus a very small value for $\mathcal{F}_{\boldsymbol{\gamma}}(\gamma)$, the distribution $\mathcal{F}_{\boldsymbol{\gamma}}$ will be used for assigning a P value based on the TLCC.

Recalling that the set G is the collection of gene pairs for which the null hypothesis H_0 is to be evaluated, we now describe how $\mathcal{F}_{\boldsymbol{\gamma}}$ will be used to compute a P value for gene pair $(g_1, g_2) \in G$. As was done for the set H, we compute the squared TLCC for each time lag $\tau \in L$ and for each pair $(g_1, g_2) \in G$, and denote it by $\varphi_\tau(g_1, g_2)$,

$$\varphi_\tau(g_1, g_2) \equiv \rho_\tau^2(g_1, g_2).$$

We use different symbol in order to make explicit φ_τ is a function from G to $[0, 1]$, whereas π_τ is a function from H to $[0, 1]$. In analogy with the fractional rank μ_τ, we compute a fractional rank ν_τ of a TLCC for a pair in G, but this rank is computed within the distribution of TLCCs from the set H:

$$\nu_\tau(g_1, g_2) = \mathcal{F}_{\boldsymbol{\pi}_\tau}\left(\varphi_\tau(g_1, g_2)\right),$$

for $(g_1, g_2) \in G$ and $\tau \in L$. We note that each ν_τ is a function from G to $[0, 1]$. Just as was done for the set H, we compute the optimal time lag (here given the symbol θ) and complementary maximum fractional rank (here given the symbol ξ):

$$\theta(g_1, g_2) = \operatorname*{argmax}_{\tau \in L}\left(\nu_\tau(g_1, g_2)\right),$$
$$\xi(g_1, g_2) = 1 - \max_{\tau \in L}\left(\nu_\tau(g_1, g_2)\right),$$

for all $(g_1, g_2) \in G$. Finally, in analogy with γ defined on the set H, we incorporate the θ and ξ values into a combined log score, to which we assign the symbol σ:

$$\sigma(g_1, g_2) = \ln\left(\xi(g_1, g_2) R(\theta(g_1, g_2))\right),$$

for any $(g_1, g_2) \in G$. We note that σ is a function from G to $\mathbb{R}$. Let us denote by $\boldsymbol{\sigma}$ the continuous random variable corresponding to the function σ. To the extent that H is an unbiased and representative sampling of gene pairs for which the null hypothesis H_0 is true, $\boldsymbol{\sigma}|H_0$ should be distributed approximately as $\boldsymbol{\gamma}$, i.e.,

$$\mathcal{F}_{\boldsymbol{\sigma}|H_0} \simeq \mathcal{F}_{\boldsymbol{\gamma}}. \tag{7}$$

Finally, the overall significance P^{tlc} (where exp stands for "expression") of the TLC is computed as

$$\begin{aligned} P^{\mathrm{tlc}}(g_1, g_2) &= \mathcal{F}_{\boldsymbol{\gamma}}(\sigma(g_1, g_2)), \\ &= \mathcal{F}_{\boldsymbol{\gamma}}\left(\ln\left(\xi(g_1, g_2) R(\theta(g_1, g_2))\right)\right), \end{aligned} \tag{8}$$

for any $(g_1, g_2) \in G$. By Eq. 7, under the null hypothesis, P^{tlc} will be distributed approximately uniformly on the unit interval. The smaller the value of $P^{\mathrm{tlc}}(g_1, g_2)$, the more unlikely is a pair of outcomes (ψ, ω) to occur by chance (under the null hypothesis) with an associated γ value smaller than or equal to $\sigma(g_1, g_2)$. For this reason, P^{tlc} is taken as the overall significance measure.

3 Prior Distribution of Transcriptional Time Lags

In this section we describe how the prior distribution for transcriptional time lags, $P(\tau_{\mathrm{c}}, \overline{H_0})$, was selected. For a transcription factor gene g_1 and a target gene g_2, the overall transcriptional regulatory time delay τ_{c} (where "c" stands for the combined gene-gene delay) can be modeled as a sum of two delays,

$$\tau_{\mathrm{c}} = \tau_{\mathrm{rna}} + \tau_{\mathrm{prot}}.$$

The time τ_{prot} represents the delay associated with the transcription factor protein, including the translation time, folding time, nuclear translocation times, and (for a down-regulated TF gene) protein half-life. The time τ_{rna} represents the delay associated with the target gene and is the time required to produce a completed mRNA, once the transcription initiation complex has been assembled on the gene's promoter. Although data for τ_{prot} and τ_{rna} for all pairs of transcription factors and target genes are not available, it is possible to estimate moments of the distribution of τ_{c}. The components of the post-transcriptional delay have been estimated as 1–3 min for translation [2], 7 ± 2.5 min for post-translational assembly [3], and 1–2 min for nuclear translocation [4], for a total protein delay of approximately 10.5 ± 4 min. Furthermore, τ_{rna} is estimated to have a mean value of 40 min and a

median value of 20 min [5], with a distribution that is skewed [5, 6, 7]. The distribution of τ_{c} over the set of all interacting pairs is therefore modeled using the gamma distribution with a mean value of 45 min and a variance of approximately 250 min^2,

$$P(\tau_{\mathrm{c}}|\overline{H_0}) = \tau_{\mathrm{c}}^{k-1}\frac{e^{-\tau_{\mathrm{c}}/w}}{\Gamma(k)w^k}, \tag{9}$$

with $k = 8$ and $w = 5.625$. An overall delay of 45 min is consistent with the upper limit of the total delay (40 min) estimated in [8]. With the choice $k = 8$, this distribution gives a small probability (less than 2%) for a transcriptional regulatory interaction with an overall (gene-gene) delay less than 10 min. Because it is conditioned on the existence of a transcriptional regulatory interaction between g_1 and g_2, we denote this probability distribution by $P(\tau_{\mathrm{c}}|\overline{H_0})$. This conditional probability distribution is discretized to obtain a conditional probability of observing each of the possible discrete time lags $\tau \in L$. Here, we assume that the set of time lags L is a uniform binning with $\Delta\tau = 10$ min. In terms of this binning,

$$P(\tau|\overline{H_0}) = \int_{\tau-\Delta\tau/2}^{\tau+\Delta\tau/2} P(\tau_{\mathrm{c}}|\overline{H_0})d\tau_{\mathrm{c}}, \tag{10}$$

for all $\tau \in L$.

4 Gaussian kernel density estimation method

In this section we provide a self-contained description of the method of Gaussian kernel density estimation, and its application to multivariate statistical data analysis. We also show that P values derived from the kernel density smoothing method will be uniformly distributed under the null hypothesis.

4.1 Notation

Let $\mathbb{R}^+$ denote the set of positive real numbers. Let I denote the closed unit interval. Let $\mathbb{N}$ denote the natural numbers, and $\mathbb{N}_0$ denote the natural numbers plus the number zero. We shall use bold Greek letters to denote random variables, and the non-bold version to denote an outcome for a specific sample point. For example, $\boldsymbol{\theta}$ would denote a random variable, and θ would denote an outcome. All random variables are real-valued unless otherwise noted. The power set of a set A will be denoted $\mathbb{P}(A)$. We shall normally abbreviate an element $(h_1, h_2) \in H$ as $h = (h_1, h_2) \in H$, and

similarly $g = (g_1, g_2) \in G$. For cases where we have a set $A = \{A_b\}_{b \in B}$ whose elements are indexed by a set B, we may use the notation $\{A_b\}$, where the index set is understood by context. For a probability space $\mathcal{S}$ and a random variable $\boldsymbol{\xi}$ on $\mathcal{S}$, we use $E(\boldsymbol{\xi})$ to denote the expectation value of $\boldsymbol{\xi}$. We shall denote the cardinality of a set A by $|A|$. We denote a vector in $\mathbb{R}^n$ ($n \in \mathbb{N}$) by $\vec{v} = (v_1 \dots v_n)$, and the Euclidean norm of the vector $\vec{v}$ in $\mathbb{R}^n$ by $\|\vec{v}\|$. We denote by $[a, b]$ the closed interval $\{x \in \mathbb{R} | a \leq x \leq b\}$, and by (a, b) the open interval $\{x \in \mathbb{R} | a < x < b\}$. For a subset $A \subset X$, $\bar{A}$ shall denote the set-theoretic complement of A within X. If $A, B \subset X$, $A - B$ denotes the relative complement of B in A. The step function $\Theta : \mathbb{R} \to \{0, 1\}$ shall be defined by

$$\Theta(x) = \begin{cases} 1, \text{if } x \geq 0, \\ 0, \text{if } x < 0, \end{cases}$$

for all $x \in \mathbb{R}$. Please note that in this document, γ does *not* denote the incomplete gamma function, in contrast to the definition of γ used in the main text.

4.2 Preliminary definitions

Let $\mathcal{A}$ denote a probability space $(A, \mathbb{P}(A), P_A)$, where A is a finite set of sample points and $P_A : \mathbb{P}(A) \to I$ is the uniform probability measure defined by

$$P_A(S) = \frac{|S|}{|A|},$$

for all $S \in \mathbb{P}(A)$.

Definition 1 *To each measurable function $\zeta : A \to \mathbb{R}$ is associated a random variable on $\mathcal{A}$, which we denote by $\boldsymbol{\zeta}$. The cumulative distribution function (CDF) $F_{\boldsymbol{\zeta}} : \mathbb{R} \to I$ associated with $\boldsymbol{\zeta}$ is defined by*

$$\begin{aligned} F_{\boldsymbol{\zeta}}(z) &= \sum_{a \in A} P_A(\{a\}) \Theta(z - \zeta(a)), \\ &= \sum_{a \in A} \frac{1}{|A|} \Theta(z - \zeta(a)). \end{aligned} \tag{11}$$

for all $z \in \mathbb{R}$. The expectation value of any random variable $\boldsymbol{\zeta}$ on $\mathcal{A}$ is given

by

$$E(\boldsymbol{\zeta}) = \sum_{a \in A} P_A(\{a\})\zeta(a),$$
$$= \frac{1}{|A|} \sum_{a \in A} \zeta(a).$$

We note that the CDF function $F_{\boldsymbol{\zeta}}$ is monotonic and satisfies

$$\lim_{z \to -\infty} F_{\boldsymbol{\zeta}}(z) = 0,$$
$$\lim_{z \to +\infty} F_{\boldsymbol{\zeta}}(z) = 1.$$

Definition 2 *For any measurable function $f : \mathbb{R} \to \mathbb{R}$ and any random variable $\boldsymbol{\zeta}$ on $\mathcal{A}$, we define the composition $f \circ \boldsymbol{\zeta}$ to be the random variable on $\mathcal{A}$ associated with the measurable function $(f \circ \zeta) : A \to \mathbb{R}$.*

For finite A, the function $F_{\boldsymbol{\zeta}}$ will not be continuous. This makes it difficult to use $F_{\boldsymbol{\zeta}}$ as a measure of significance for a value $z \in \mathbb{R}$ obtained from extending ζ outside the set A (e.g., supposing that A is a representative set of sample points for which the null hypothesis is true, one often wishes to extend ζ to sample points for which the likelihood of observing ζ, given the null hypothesis, is to be evaluated). It is therefore convenient to define a smooth CDF that approximates $F_{\boldsymbol{\zeta}}$. Let $\mathcal{K} : \mathbb{R} \to \mathbb{R}^+$ be a continuous function such that

$$\lim_{x \to \pm\infty} \mathcal{K}(x) = 0,$$

where $x \in \mathbb{R}$, and

$$\int_{-\infty}^{\infty} \mathcal{K}(x)dx = 1. \tag{12}$$

Using the kernel $\mathcal{K}$, a smoothed probability density function (PDF) $\mathcal{D}_{\boldsymbol{\zeta}} : \mathbb{R} \to \mathbb{R}^+$ can be obtained,

$$\mathcal{D}_{\boldsymbol{\zeta},\mathcal{K}}(z) = \sum_{a \in A} P_A(\{a\})\mathcal{K}(z - \zeta(a)),$$
$$= \frac{1}{|A|} \sum_{a \in A} \mathcal{K}(z - \zeta(a)). \tag{13}$$

It follows immediately by Eqs. 12 and 13 that $\mathcal{D}_{\boldsymbol{\zeta},\mathcal{K}}$ satisfies the rule of total probability,

$$\int_{-\infty}^{+\infty} \mathcal{D}_{\boldsymbol{\zeta},\mathcal{K}}(z)dz = 1.$$

Given $\mathcal{D}_{\boldsymbol{\zeta},\mathcal{K}}$, a smoothed cumulative distribution function (CDF) $\mathcal{F}_{\boldsymbol{\zeta},\mathcal{K}} : \mathbb{R} \to I$ is defined by

$$\mathcal{F}_{\boldsymbol{\zeta},\mathcal{K}}(z) = \int_{-\infty}^{z} \mathcal{D}_{\boldsymbol{\zeta},\mathcal{K}}(z')dz'$$

for all $z \in \mathbb{R}$. It follows directly that

$$\lim_{z\to-\infty} \mathcal{F}_{\boldsymbol{\zeta},\mathcal{K}}(z) = 0, \tag{14}$$

$$\lim_{z\to+\infty} \mathcal{F}_{\boldsymbol{\zeta},\mathcal{K}}(z) = 1. \tag{15}$$

Because $\mathcal{D}_{\boldsymbol{\zeta},\mathcal{K}}(z) > 0$ for all $z \in \mathbb{R}$, the function $\mathcal{F}_{\boldsymbol{\zeta},\mathcal{K}}$ is strictly monotonically increasing, and thus onto.

4.3 Univariate Gaussian kernel density estimation

A particularly useful family of kernels is based on the Gaussian. Let $\{\mathcal{G}_s\}_{s\in\mathbb{R}^+} : \mathbb{R} \to \mathbb{R}^+$ be a family of maps defined by

$$\mathcal{G}_s(x) = \frac{1}{\sqrt{2\pi}s} e^{-\frac{x^2}{2s^2}}, \tag{16}$$

for all $x \in \mathbb{R}$ and for all $s \in \mathbb{R}^+$. The parameter s is called the smoothing length. Taking $\mathcal{K} = \mathcal{G}_s$, the PDF function $\mathcal{D}_{\boldsymbol{\zeta},\mathcal{G}_s}$ is said to be the Gaussian kernel density smoothed PDF of $\boldsymbol{\zeta}$, which for simplicity we will denote by $\mathcal{D}_{\boldsymbol{\zeta}_s}$. It is convenient to formalize this definition.

Definition 3 *Let $\mathcal{A} = (A, \mathbb{P}(A), P_A)$ be a probability space with A finite and P_A uniform. Let $\zeta : A \to \mathbb{R}$ be measurable, and denote by $\boldsymbol{\zeta}$ the associated random variable on $\mathcal{A}$. Let $s \in \mathbb{R}^+$. We define the Gaussian kernel density smoothed PDF function $\mathcal{D}_{\boldsymbol{\zeta}_s} : \mathbb{R} \to \mathbb{R}^+$ and CDF function $\mathcal{F}_{\boldsymbol{\zeta}_s} : \mathbb{R} \to I$ of ζ, on the space $\mathcal{A}$, by the equations*

$$\mathcal{D}_{\boldsymbol{\zeta}_s}(z) = \frac{1}{\sqrt{2\pi}|A|s} \sum_{a\in A} e^{-\frac{(z-\zeta(a))^2}{2s^2}}, \tag{17}$$

$$\mathcal{F}_{\boldsymbol{\zeta}_s}(z) = \int_{-\infty}^{z} \mathcal{D}_{\boldsymbol{\zeta}_s}(z')dz', \tag{18}$$

for all $z \in \mathbb{R}$.

The notation $\mathcal{D}_{\boldsymbol{\zeta}_s}$ and $\mathcal{F}_{\boldsymbol{\zeta}_s}$ shall be used in place of the equivalent (but more cumbersome) notation, $\mathcal{D}_{\boldsymbol{\zeta}_s,\mathcal{G}_s}$ and $\mathcal{F}_{\boldsymbol{\zeta}_s,\mathcal{G}_s}$. The function $\mathcal{F}_{\boldsymbol{\zeta}_s}$ can be formally

represented in terms of the error function $\mathrm{erf}(x)$,

$$\mathcal{F}_{\boldsymbol{\zeta}_s}(z) = \frac{1}{2|A|} \sum_{a \in A} \left(1 + \mathrm{erf}\left(\frac{z - \zeta(a)}{\sqrt{2}s} \right) \right). \tag{19}$$

For $s \in \mathbb{R}^+$, this function is continuously differentiable. For the sake of completeness, we note that, by the definition of $\mathcal{F}_{\boldsymbol{\zeta}_s}$ in Definition 3 above,

$$\mathcal{D}_{\boldsymbol{\zeta}_s}(z) = \frac{d}{dz}\mathcal{F}_{\boldsymbol{\zeta}_s}(z),$$

for all $z \in \mathbb{R}$.

Proposition 1 *Given $\mathcal{F}_{\boldsymbol{\zeta}_s}$ and $F_{\boldsymbol{\zeta}}$ defined by Eqs. 18 and 11, respectively, the following holds everywhere except perhaps on a set of measure zero:*

$$\lim_{s \to 0^+} \mathcal{F}_{\boldsymbol{\zeta}_s} = F_{\boldsymbol{\zeta}}.$$

Proof Take the $s \to 0^+$ limit of Eq. 19, and use the definition of the error function, to obtain the desired result. ∎

Thus for $s > 0$, $\mathcal{F}_{\boldsymbol{\zeta}_s}$ is a smooth function approximating $F_{\boldsymbol{\zeta}}$. Given that $\mathcal{D}_{\boldsymbol{\zeta}_s}$ is continuous and positive-definite (see Eq. 17), $\mathcal{F}_{\boldsymbol{\zeta}_s}$ is continuous and strictly monotonically increasing; therefore, it is invertible. The function $\mathcal{F}_{\boldsymbol{\zeta}_s}$ can be interpreted as the CDF of a continuous, real-valued random variable $\boldsymbol{\zeta}_s$ on a different probability space, as we now explain. Let $\mathcal{I} \equiv (I, \mathfrak{B}, P_I)$ be the probability space with σ-algebra $\mathfrak{B}$ and unit probability measure $P_I : \mathfrak{B} \to I$.

Definition 4 *Given a map $\zeta : A \to \mathbb{R}$ and the associated Gaussian kernel density smoothed PDF $\mathcal{D}_{\boldsymbol{\zeta}_s} : \mathbb{R} \to \mathbb{R}^+$ and CDF $\mathcal{F}_{\boldsymbol{\zeta}_s} : \mathbb{R} \to I$ as defined in Definition 3, let $\zeta_s : I \to \mathbb{R}$ be the map defined by*

$$\zeta_s(u) = \mathcal{F}_{\boldsymbol{\zeta}_s}^{-1}(u),$$

for all $u \in I$. The map ζ_s constitutes a continuous random variable on $\mathcal{I}$, denoted $\boldsymbol{\zeta}_s$, for which the value at sample point $u \in I$ is given by $\zeta_s(u)$. For any continuous function $f : \mathbb{R} \to \mathbb{R}$, the expectation value of $f(\boldsymbol{\zeta}_s)$ on $\mathcal{I}$ is given by

$$E\left(f(\boldsymbol{\zeta}_s)\right) \equiv \int_0^1 f(\zeta_s(u))du.$$

Proposition 2 *Given $\mathcal{F}_{\boldsymbol{\zeta}_s}$ defined as in Definition 3 and $\boldsymbol{\zeta}_s$ defined as in Definition 4, the CDF of $\boldsymbol{\zeta}_s$ is precisely $\mathcal{F}_{\boldsymbol{\zeta}_s}$.*

Proof Compute $P_I(\{u \in I | \zeta_s(u) \leq z\})$ for all $z \in \mathbb{R}$. Use the definition of $\boldsymbol{\zeta}_s$, and the fact that $\mathcal{F}_{\boldsymbol{\zeta}_s}$ is strictly monotonic. ∎

As a consequence of Proposition 2, by differentiation, we obtain that the continuous random variable $\boldsymbol{\zeta}_s$ is distributed according to the PDF $\mathcal{D}_{\boldsymbol{\zeta}_s}$. To summarize, given a random variable $\boldsymbol{\zeta}$ on a discrete probability space $\mathcal{A}$, and a smoothing length $s \in \mathbb{R}^+$, we can obtain a smooth CDF and PDF from Definition 3, and a corresponding continuous random variable $\boldsymbol{\zeta}_s$ on the probability space $\mathcal{I}$ using Definition 4. We now prove a key limit equivalence between expectation values involving $\boldsymbol{\zeta}$, and expectation values involving $\boldsymbol{\zeta}_s$.

Proposition 3 *For $\boldsymbol{\zeta}$ and $\boldsymbol{\zeta}_s$ defined in Definitions 3 and 4, and for any continuous function $f : \mathbb{R} \to \mathbb{R}$,*

$$\lim_{s \to 0^+} E\left(f(\boldsymbol{\zeta}_s)\right) = E\left(f(\boldsymbol{\zeta})\right).$$

Proof Any continuous function is measurable, so $f(\boldsymbol{\zeta})$ is a random variable on $\mathcal{A}$, and $f(\boldsymbol{\zeta}_s)$ is a random variable on $\mathcal{I}$. Use Definition 4, and perform a change-of-variable $u = \mathcal{F}_{\boldsymbol{\zeta}_s}(z)$ under the integral. Note that the interchange of the $s \to 0^+$ limit and the integral is permissible in the last step, because the integrand is uniformly continuous in s. Use the fact that the zero-variance limit of a Gaussian is the Dirac delta distribution $\delta(x)$. ∎

Proposition 3 implies that for sufficiently small s, any order moment of $\boldsymbol{\zeta}_s$ will approximate the corresponding moment of $\boldsymbol{\zeta}$. In this sense, the distribution of the continuous random variable $\boldsymbol{\zeta}_s$ approximates the distribution of $\boldsymbol{\zeta}$. This result enables us to approximately compute the expectation value of any function involving $\boldsymbol{\zeta}$ on the discrete probability space $\mathcal{A}$, as an expectation value (on the space $\mathcal{I}$) of the corresponding function of $\boldsymbol{\zeta}_s$.

4.4 Multivariate Gaussian kernel density estimation

The above construction of a PDF $\mathcal{D}_{\boldsymbol{\zeta}_s}$ from a map ζ is useful when we are concerned only with the univariate probability distribution of a particular variable, say, $\boldsymbol{\zeta}_s$. However, in many cases, we will have a collection of maps, and will wish to obtain a smoothed multivariate PDF for the collection.

Definition 5 *Let $\mathcal{A} = (A, \mathbb{P}(A), P_A)$ be a probability space over a finite set A with uniform probability measure P_A. Let $n \in \mathbb{N}$ and $N = \{1, \ldots, n\}$. Let $\{\zeta_i\}_{i \in N} : A \to \mathbb{R}$ be a family of maps, which we can write as a single vector-valued map $\vec{\zeta} : A \to \mathbb{R}^n$. Let $\{\boldsymbol{\zeta}_i\}_{i \in N}$ be the random variables on $\mathcal{A}$ associated with the maps $\vec{\zeta}$, which we can also denote by $\vec{\boldsymbol{\zeta}}$. Let $s \in \mathbb{R}^+$. The Gaussian kernel density smoothed joint PDF of $\{\zeta_i\}_{i \in D}$ on the space $\mathcal{A}$ shall be defined as a map $\mathcal{D}_{\vec{\boldsymbol{\zeta}}_s} : \mathbb{R}^n \to \mathbb{R}^+$ satisfying*

$$\mathcal{D}_{\vec{\boldsymbol{\zeta}}_s}(\vec{z}) = \sum_{a \in A} \frac{1}{(2\pi)^{\frac{n}{2}} s^n |A|} e^{-\frac{\|\vec{z} - \vec{\zeta}(a)\|^2}{2s^2}}, \tag{20}$$

for all $\vec{z} \in \mathbb{R}^n$. The corresponding joint CDF is a continuously differentiable map $\mathcal{F}_{\vec{\boldsymbol{\zeta}}_s} : \mathbb{R}^n \to I$ satisfying

$$\mathcal{F}_{\vec{\boldsymbol{\zeta}}_s}(\vec{z}) = \int_{-\infty}^{z_1} dz_1' \cdots \int_{-\infty}^{z_n} dz_n' \mathcal{D}_{\vec{\boldsymbol{\zeta}}_s}(\vec{z}'), \tag{21}$$

for all $\vec{z} \in \mathbb{R}^n$.

It should be noted that Definition 5 does not depend on the $\{\boldsymbol{\zeta}_i\}_{i \in N}$ being independent.

Proposition 4 *The marginal Gaussian kernel density smoothed probability distribution for ζ_i is the same as the univariate kernel density smoothed distribution for ζ_i from Definition 3.*

Proof Recall from Definition 3 the formulas for the univariate Gaussian kernel density smoothed PDF $\mathcal{D}_{(\boldsymbol{\zeta}_i)_s} : \mathbb{R} \to \mathbb{R}^+$ and CDF $\mathcal{F}_{(\boldsymbol{\zeta}_i)_s} : \mathbb{R} \to I$ for each map ζ_i. (For simplicity, we shall henceforth denote these two functions by $\mathcal{D}_{\boldsymbol{\zeta}_{i,s}}$ and $\mathcal{F}_{\boldsymbol{\zeta}_{i,s}}$). The marginal distribution for ζ_i is obtained by integrating $\mathcal{D}_{\vec{\boldsymbol{\zeta}}_s}$ over any hyperplane of $\mathbb{R}^n$ with z_i held fixed. ∎

The function $\mathcal{D}_{\vec{\boldsymbol{\zeta}}_s}$ satisfies the rule of total probability,

$$\int_{-\infty}^{+\infty} dz_1 \cdots \int_{-\infty}^{+\infty} dz_n \mathcal{D}_{\vec{\boldsymbol{\zeta}}_s}(\vec{z}) = 1.$$

Since $\mathcal{D}_{\vec{\boldsymbol{\zeta}}_s}(\vec{z}) > 0$ for all $\vec{z} \in \mathbb{R}^n$, it follows that $\mathcal{F}_{\vec{\boldsymbol{\zeta}}_s}$, is strictly monotonic in any of its variables taken one at a time, with the other variables being held fixed and nonzero. By Sklar's Theorem [9], given $\mathcal{F}_{\vec{\boldsymbol{\zeta}}_s}$, there exists a continuous function $\mathcal{C}_{\vec{\boldsymbol{\zeta}}_s} : I^n \to I$ such that

$$\mathcal{C}_{\vec{\boldsymbol{\zeta}}_s}\left(\mathcal{F}_{\boldsymbol{\zeta}_{1,s}}(z_1), \ldots, \mathcal{F}_{\boldsymbol{\zeta}_{n,s}}(z_n)\right) = \mathcal{F}_{\vec{\boldsymbol{\zeta}}_s}(\vec{z}), \tag{22}$$

for all $\vec{z} \in \mathbb{R}^n$. The function $\mathcal{C}_{\vec{\zeta}_s}$ is called a copula. It follows from the previous equation that

$$\mathcal{C}_{\vec{\zeta}_s}(1, \ldots, 1, u_i, 1, \ldots, 1) = u_i, \qquad (23)$$
$$\mathcal{C}_{\vec{\zeta}_s}(u_1, \ldots, u_{i-1}, 0, u_{i+1}, \ldots, u_n) = 0,$$

for all $\vec{u} \in I^n$ and for all $i \in N$. As a corollary, we have

$$\mathcal{C}_{\vec{\zeta}_s}(1, \ldots, 1) = 1, \qquad (24)$$

so $\mathcal{C}$ is onto. Furthermore, since the $\mathcal{F}_{\zeta_{i,s}}$ are strictly monotonic (for all $i \in N$), and since $\mathcal{F}_{\vec{\zeta}_s}$ is strictly monotonic in any one of its variables when the other variables are nonzero and held fixed, it follows from Eq. 22 that $\mathcal{C}_{\vec{\zeta}_s}$ is strictly monotonic as a function of any one of its variables, when the other variables are nonzero and held fixed. We shall now construct a σ-algebra $\mathfrak{F}$ on I^n, and an associated probability measure $P_{I^n} : \mathfrak{F} \to \mathbb{R}^+$ as follows. Let $V(\vec{u})$ be a box with one corner at the origin in I^n and the opposite corner given by the vector $\vec{u} \in I^n$,

$$V(\vec{u}) \equiv [0, u_1] \times \cdots \times [0, u_n], \qquad (25)$$

for any $\vec{u} \in I^n$. We are now in a position to define $\mathfrak{F}$ as a set consisting of all possible countable unions of such boxes and their complements, i.e., all possible subsets $S \subseteq I^n$ of the form

$$S = V(\vec{u}_1) \cup \cdots \cup V(\vec{u}_i) \cup \overline{V(\vec{u}_{i+1})} \cup \cdots \cup \overline{V(\vec{u}_j)}, \qquad (26)$$

for all $i, j \in \mathbb{N}_0$ such that $i \leq j$, and where $\vec{u}_k \in I^n$ for all k. It is easy to see from Eq. 26 that $\mathfrak{F}$ is closed under complementation and under countable unions of any of its members. Thus, $\mathfrak{F}$ is a σ-algebra on I^n. We shall now define the measure P_{I^n} generatively. Starting with the corner box ("cbox") set $V(\vec{u})$, we define

$$P_{I^n}(V(\vec{u})) = C_{\vec{\zeta}_s}(u_1, \ldots, u_n), \qquad (27)$$

for all $\vec{u} \in I^n$. On the complement corner box, or "ccbox", we define

$$P_{I^n}\left(\overline{V(\vec{u})}\right) = 1 - P_{I^n}(V(\vec{u})),$$
$$= 1 - C_{\vec{\zeta}_s}(u_1, \ldots, u_n), \qquad (28)$$

for all $\vec{u} \in I^n$. The density of $\mathcal{C}$ per unit n-dimensional volume of sample space is given by a function $\mathcal{Q} : I^n \to \mathbb{R}^+$ satisfying

$$\int_0^{u_1} du_1' \cdots \int_0^{u_n} du_n' \mathcal{Q}(\vec{u}') = \mathcal{C}(\vec{u}),$$

or equivalently,

$$\mathcal{Q}(\vec{u}) = \frac{d}{du_1} \cdots \frac{d}{du_n} \mathcal{C}(\vec{u}), \tag{29}$$

for all $\vec{u} \in I^n$. Using $\mathcal{Q}$, we can now define the measure on the arbitrary set $S \in \mathfrak{F}$,

$$P_{I^n}(S) = \int_S d\mathcal{C} = \int_S d^n u \mathcal{Q}(\vec{u}), \tag{30}$$

which reduces to Eqs. 27 and 28 when $S = V(\vec{u})$ and $S = \overline{V(\vec{u})}$, respectively. By the fact that $\mathcal{Q} \geq 0$, it follows that $P_{I^n}(S) \geq 0$ for all $S \in \mathfrak{F}$, and thus, P_{I^n} satisfies the first probability axiom. Computing $P_{I^n}(I^n)$, we find

$$\begin{aligned} P_{I^n}(I^n) &= \int_{I^n} d^n u \mathcal{Q}(\vec{u}), \\ &= \mathcal{C}(1, 1, \ldots, 1), \\ &= 1, \end{aligned}$$

with the last step due to Eq. 24. Thus, P_{I^n} satisfies the second probability axiom. Finally, for a countable sequence of disjoint sets $\{S_i\}_{i \in \mathbb{N}}$,

$$S = S_1 \cup S_2 \cup \cdots,$$

we find

$$\begin{aligned} P_{I^n}(S) &= P_{I^n}(S_1 \cup S_2 \cup \cdots), \\ &= \int_S d^n u \mathcal{Q}(\vec{u}), \\ &= \sum_i \int_{S_i} d^n u \mathcal{Q}(\vec{u}), \\ &= \sum_i P_{I^n}(S_i), \end{aligned}$$

and thus, P_{I^n} satisfies the third probability axiom. Thus, P_{I^n} is a probability measure on $\mathfrak{F}$, and the ordered triple $(I^n, \mathfrak{F}, P_{I^n})$ is a probability space, which we shall call $\mathcal{I}^n$.

Definition 6 *Given the family of maps* $\{\zeta_i\}_{i\in N} : A \to \mathbb{R}$ *and the associated univariate Gaussian kernel density smoothed PDFs* $\mathcal{D}_{\boldsymbol{\zeta}_{i,s}} : \mathbb{R} \to \mathbb{R}^+$ *and CDF* $\mathcal{F}_{\boldsymbol{\zeta}_{i,s}} : \mathbb{R} \to I$ *(with* $s \in \mathbb{R}^+$*) defined in Eqs. 20 and 21, we define a family of maps* $\{\widehat{\zeta}_{i,s}\}_{i\in N} : I^n \to \mathbb{R}$*, by*

$$\widehat{\zeta}_{i,s}(\vec{u}) = \mathcal{F}^{-1}_{\boldsymbol{\zeta}_{i,s}}(u_i), \tag{31}$$

for all $\vec{u} \in I^n$ *and for all* $i \in N$*. Since these maps are continuous, they constitute a family of random variables on* $\mathcal{I}^n$*, which we denote by* $\{\widehat{\boldsymbol{\zeta}}_{\boldsymbol{i,s}}\}_{i\in N}$*. For any continuous function* $f : \mathbb{R}^n \to \mathbb{R}$*, the expectation value of* $f(\{\widehat{\boldsymbol{\zeta}}_{i,s}\})$ *is given by*

$$E(f(\{\widehat{\boldsymbol{\zeta}}_{i,s}\})) = \int_0^1 du_1 \cdots \int_0^1 du_n \mathcal{Q}(\vec{u}) f(\{\widehat{\zeta}_{i,s}(\vec{u})\}),$$

where $\mathcal{Q}$ *is defined in Eq. 29*

Proposition 5 *For any* $i \in N$*, the marginal CDF of* $\widehat{\boldsymbol{\zeta}}_{i,s}$*, defined in Eq. 31, is given by* $\mathcal{F}_{\boldsymbol{\zeta}_{i,s}}$*.*

Proof We wish to evaluate $P_{I^n}(\{\vec{u} \in I^n | \widehat{\zeta}_{i,s}(\vec{u}) \le z\})$, for all $z \in \mathbb{R}$ and $i \in N$. Use the definition of $\widehat{\zeta}_{i,s}$ above, and then use the fact that $\mathcal{F}_{\boldsymbol{\zeta}_{i,s}}$ is strictly monotonic and onto, to compose $\mathcal{F}_{\boldsymbol{\zeta}_{i,s}}$ on both sides of the inequality, yielding

$$P_{I^n}(\{\vec{u} \in I^n | \mathcal{F}^{-1}_{\boldsymbol{\zeta}_{i,s}}(u_i) \le z\}) = P_{I^n}(\{\vec{u} \in I^n | u_i \le \mathcal{F}_{\boldsymbol{\zeta}_{i,s}}(z)\}).$$

We recognize the set under the curly braces as a box which can be represented using V defined in Eq. 25,

$$P_{I^n}(\{\vec{u} \in I^n | u_i \le \mathcal{F}_{\boldsymbol{\zeta}_{i,s}}(z)\}) = P_{I^n}(V(1, \ldots, 1, \mathcal{F}_{\boldsymbol{\zeta}_{i,s}}(z), 1, \ldots, 1)).$$

This can be evaluated using Eq. 27 and Eq. 23 to obtain the desired result,

$$P_{I^n}(\{\vec{u} \in I^n | \widehat{\zeta}_{i,s}(\vec{u}) \le z\}) = \mathcal{F}_{\boldsymbol{\zeta}_{i,s}}(z).$$

∎

Having established that the marginal CDF of $\widehat{\boldsymbol{\zeta}}_{i,s}$ is the same as the univariate CDF of $\boldsymbol{\zeta}_{i,s}$, we now dispense with the hat symbol. We shall denote the collection of random variables $\{\boldsymbol{\zeta}_{i,s}\}_{i\in N}$ by the vector notation, $\vec{\boldsymbol{\zeta}}_s$.

Proposition 6 *The joint CDF of* $\{\boldsymbol{\zeta}_{i,s}\}_{i\in N}$ *is* $\mathcal{F}_{\vec{\boldsymbol{\zeta}}_s}$.

Proof We wish to evaluate $P_{I^n}(\{\vec{u}\in I^n|\zeta_{i,s}(\vec{u})\le z_i,\forall i\in N\})$. As for the case with the marginal distribution, we use Eqs. 25, 27, and 22,

$$\begin{aligned}
P_{I^n}(\{\vec{u}\in I^n|\zeta_{i,s}(\vec{u})\le z_i,\forall i\in N\}) &= P_{I^n}(\{\vec{u}\in I^n|u_i\le \mathcal{F}_{\boldsymbol{\zeta}_{i,s}}(z_i),\forall i\in N\}),\\
&= P_{I^n}(V(\mathcal{F}_{\boldsymbol{\zeta}_{1,s}}(z_1),\ldots,\mathcal{F}_{\boldsymbol{\zeta}_{n,s}}(z_n))),\\
&= \mathcal{C}(\mathcal{F}_{\boldsymbol{\zeta}_{1,s}}(z_1),\ldots,\mathcal{F}_{\boldsymbol{\zeta}_{n,s}}(z_n)),\\
&= \mathcal{F}_{\vec{\boldsymbol{\zeta}}_s}(\vec{z}).
\end{aligned}$$

This proves the desired result. ∎

Proposition 7 *Given any continuous function* $f:\mathbb{R}^n\to\mathbb{R}$, *given* $\vec{\boldsymbol{\zeta}}$ *defined in Definition 5, and given* $\vec{\boldsymbol{\zeta}}_s=\{\boldsymbol{\zeta}_{i,s}\}_{i\in N}$ *where the* $\{\boldsymbol{\zeta}_{i,s}\}_{i\in N}$ *are defined as in Definition 6,*

$$\lim_{s\to 0^+}E(f(\vec{\boldsymbol{\zeta}}_s))=E(f(\vec{\boldsymbol{\zeta}})).$$

Proof

$$\begin{aligned}
E(f(\vec{\boldsymbol{\zeta}}_s)) &= \int_{I^n} d^n u\,\mathcal{Q}(\vec{u})f(\mathcal{F}^{-1}_{\boldsymbol{\zeta}_{1,s}}(u_1),\ldots,\mathcal{F}^{-1}_{\boldsymbol{\zeta}_{n,s}}(u_n)),\\
&= \int_{\mathbb{R}^n} d^n z\,f(\vec{z})\left(\prod_{i\in N}\mathcal{D}_{\boldsymbol{\zeta}_{i,s}}(z_i)\right)\mathcal{Q}\left(\mathcal{F}_{\boldsymbol{\zeta}_{1,s}}(z_1),\ldots,\mathcal{F}_{\boldsymbol{\zeta}_{n,s}}(z_n)\right),\\
&= \int_{\mathbb{R}^n} d^n z\,f(\vec{z})\mathcal{D}_{\vec{\boldsymbol{\zeta}}_s}(\vec{z}).
\end{aligned}$$

Note that the change of variable $\vec{z}=\{z_i\}_{i\in N}$, where $z_i=\mathcal{F}^{-1}_{\boldsymbol{\zeta}_{i,s}}(u_i)$ for all $i\in N$, has been used after the first line. Observing that the integrand above is uniformly continuous in s, and taking the limit $s\to 0^+$, we find

$$\begin{aligned}
\lim_{s\to 0^+}E(f(\vec{\boldsymbol{\zeta}}_s)) &= \int_{\mathbb{R}^n} d^n z\,f(\vec{z})\lim_{s\to 0^+}\mathcal{D}_{\vec{\boldsymbol{\zeta}}_s}(\vec{z}),\\
&= \frac{1}{|A|}\sum_{a\in A}\int_{\mathbb{R}^n} d^n z\,f(\vec{z})\delta(\vec{z}-\vec{\zeta}(a)),\\
&= E(f(\vec{\boldsymbol{\zeta}})),
\end{aligned}$$

which completes the proof. ∎

Definition 7 *The random variables* $\{\boldsymbol{\zeta}_{i,s}\}_{i \in N}$ *are said to be independent if and only if their multivariate PDF* $\mathcal{D}_{\vec{\boldsymbol{\zeta}}_s}$ *is separable, i.e., it satisfies*

$$\mathcal{D}_{\vec{\boldsymbol{\zeta}}_s}(\vec{z}) = \prod_{i=1}^{n} \mathcal{D}_{\boldsymbol{\zeta}_{i,s}}(z_i),$$

in terms of the univariate densities $\{\mathcal{D}_{\boldsymbol{\zeta}_{i,s}}\}$, *for all* $\vec{z} \in \mathbb{R}^n$.

We have presented a framework for approximately computing arbitrary moments of a random variable $\boldsymbol{\zeta}$ on the probability space $\mathcal{A}$ with finite sample set A. The appropriate estimate is shown to be the corresponding moment of a continuous random variable $\boldsymbol{\zeta}_s$ (on the probability space $\mathcal{I}$) which is induced from the Gaussian kernel density smoothing of the discrete PDF for $\boldsymbol{\zeta}$. We have also generalized the framework to a family of discrete random variables $\{\boldsymbol{\zeta}_i\}$, obtaining a corresponding family of continuous random variables $\{\boldsymbol{\zeta}_{i,s}\}$ on the probability space $\mathcal{I}^n$, and established the asymptotic equivalence of expectation values of scalar functions of $\vec{\boldsymbol{\zeta}}$ and $\vec{\boldsymbol{\zeta}}_s$. Having established this asymptotic equivalence, we shall henceforth exclusively work with the continuous random variable $\boldsymbol{\zeta}_s$ obtained using Gaussian kernel density estimation for a specific value of s (see Materials and Methods, main text), and for notational clarity, the s subscript will be now be dropped. Thus, the symbol $\boldsymbol{\zeta}$ shall be understood as the continuous random variable whose density distribution is the Gaussian kernel density smoothed PDF (see Definition 4).

4.5 Uniform distribution of CDF of a random variable

We now state a crucial property of a random variable derived from the CDF of another random variable. To begin with, let $B \subseteq \mathbb{R}$ be nonempty and connected (and not a single-point set). Let $\gamma : I \to B$ be a surjection, and let $\boldsymbol{\gamma}$ be the associated random variable on $\mathcal{I}$, which we assume is continuously distributed on B (in a sense that will be defined shortly). The CDF $F_{\boldsymbol{\gamma}} : B \to I$ can be defined as the probability that $\boldsymbol{\gamma} \leq u$, over the space $\mathcal{I}$,

$$F_{\boldsymbol{\gamma}}(b) \equiv P_I\left(\left\{u' \in I | \gamma(u') \leq b\right\}\right). \tag{32}$$

for all $b \in B$. The function $F_{\boldsymbol{\gamma}}$ also satisfies the same limits as Eqs. 14 and 15, and it is monotonically increasing. Let us also construct the probability density function (PDF) of $\boldsymbol{\gamma}$, which is a map $D_{\boldsymbol{\gamma}} : B \to \mathbb{R}^+$ defined by

$$D_{\boldsymbol{\gamma}}(b) = \frac{d}{db} F_{\boldsymbol{\gamma}}(b), \tag{33}$$

for all $b \in B$. We assume that the CDF $F_{\boldsymbol{\gamma}}$ is continuously differentiable, so that D_{γ} is continuous on B (with the topology inherited from $\mathbb{R}$), i.e., that $\boldsymbol{\gamma}$ is continuously distributed. We now prove a lemma about $F_{\boldsymbol{\gamma}}$.

Lemma 1 *Given γ, B, and $F_{\boldsymbol{\gamma}}$ defined as above, $F_{\boldsymbol{\gamma}}$ is strictly monotonic.*

Proof Assume that $F_{\boldsymbol{\gamma}}$ is not strictly monotonic. Since $F_{\boldsymbol{\gamma}}$ is by definition monotonic (and assumed to be continuously differentiable), and since B is connected, there must exist an interval $Q \subset B$ on which $F_{\boldsymbol{\gamma}}$ is constant. Then $D_{\boldsymbol{\gamma}}(Q) = 0$, which implies that

$$P_I\left(\{u \in I|\inf(Q) \leq \gamma(u) \leq \sup(Q)\}\right) = 0, \tag{34}$$

implying that γ is not a surjection, which is a contradiction. This proves the Lemma. ∎

As a corollary, we note that the function $\mathcal{F}_{\boldsymbol{\zeta}}$ obtained using Gaussian kernel density estimation, is strictly monotonic (which was deduced previously following Definition 3.

Proposition 8 *Let $\boldsymbol{\gamma}$ be a continuously distributed random variable on $\mathcal{I}$, such that the associated map $\gamma : I \to B$ is a surjection, and where $B \subseteq \mathbb{R}$ is connected and nonempty (and not a single-point set). Let $\eta : I \to I$ be a map defined by*

$$\eta(u) = P_I\left(\{u' \in I|\gamma(u') \leq \gamma(u)\}\right) \equiv \left(F_{\boldsymbol{\gamma}} \circ \gamma\right)(u) \tag{35}$$

for all $u \in I$. Then the associated random variable on $\mathcal{I}$, called $\boldsymbol{\eta}$, is uniformly distributed on the unit interval.

Proof Let $F_{\boldsymbol{\eta}} : I \to I$ be the CDF of $\boldsymbol{\eta}$. Since $\boldsymbol{\eta}$ is a random variable on $\mathcal{I}$, by the same definition as in Eq. 32, $F_{\boldsymbol{\eta}}$ obeys

$$F_{\boldsymbol{\eta}}(u) = P_I\left(\{u' \in I|\eta(u') \leq u\}\right), \tag{36}$$

for all $u \in I$. Using Eq. 35,

$$P_I\left(\{u' \in I|\eta(u') \leq u\}\right) = P_I\left(\{u' \in I|F_{\boldsymbol{\gamma}}(\gamma(u')) \leq u\}\right). \tag{37}$$

Since $F_{\boldsymbol{\gamma}}$ is continuous and strictly monotonic, $F_{\boldsymbol{\gamma}}^{-1} : I \to B$ exists and is a surjection, and we obtain

$$P_I\left(\{u' \in I|F_{\boldsymbol{\gamma}}(\gamma(u')) \leq u\}\right) = P_I\left(\{u' \in I|\gamma(u') \leq F_{\boldsymbol{\gamma}}^{-1}(u)\}\right). \tag{38}$$

Since γ is onto, for any $u \in I$, there exists at least one element $v \in I$ such that $\gamma(v) = F^{-1}(u)$. Select any map $g : I \to I$ such that for all $u \in I$,

$$\gamma(g(u)) = F^{-1}(u). \tag{39}$$

We then have

$$P_I\left(\left\{u' \in I | \gamma(u') \leq F_{\boldsymbol{\gamma}}^{-1}(u)\right\}\right) = P_I\left(\left\{u' \in I | \gamma(u') \leq \gamma\left(g(u)\right)\right\}\right). \tag{40}$$

By Eq. 32, this is just

$$P_I\left(\left\{u' \in I | \gamma(u') \leq \gamma\left(g(u)\right)\right\}\right) = F_{\boldsymbol{\gamma}}\left(\gamma(g(u))\right), \tag{41}$$

which by Eq. 39 is just

$$P_I\left(\left\{u' \in I | \gamma(u') \leq \gamma\left(g(u)\right)\right\}\right) = u. \tag{42}$$

We have thus established that

$$F_{\boldsymbol{\eta}}(u) = u, \tag{43}$$

and therefore, $\boldsymbol{\eta}$ is uniformly distributed on the unit interval. ∎

The random variable $\boldsymbol{\zeta}_s$ defined in Definition 4 (now called $\boldsymbol{\zeta}$) has a probability distribution $\mathcal{D}_{\boldsymbol{\zeta}}$ over $\mathbb{R}$ that is continuous and positive-definite. Therefore by Proposition 8, the random variable on the probability space $\mathcal{I}$ defined by $\mathcal{F}_{\boldsymbol{\zeta}}(\boldsymbol{\zeta}) = \mathcal{F}_{\boldsymbol{\zeta}} \circ \boldsymbol{\zeta}$ is uniformly distributed on the unit interval.

5 Independence Proofs

In this section, we show that $\boldsymbol{\psi}$ and $\boldsymbol{\omega}$ defined as in Eqs. 5–6, with certain reasonable assumptions about $\mathcal{D}_{\boldsymbol{\pi}_\tau}$, are independent. We then show that this implies that $\boldsymbol{\omega}$ and $R(\boldsymbol{\psi})$ are independent.

Recall that our starting point are the maps $\pi_\tau : H \to \mathbb{R}^+$. By kernel density estimation as described in Definition 5, we obtain the joint CDF $\mathcal{F}_{\vec{\boldsymbol{\pi}}} : \mathbb{R}^l \to I$ for the maps $\{\pi_\tau\}_{\tau \in L}$. By Definition 6, the maps $\{\pi_\tau\}$ are associated with a family of continuous random variables $\{\boldsymbol{\pi}_\tau\}_{\tau \in L}$ on $\mathcal{I}^l$. Recall that in Eq. 4, by composing $\mathcal{F}_{\boldsymbol{\pi}_\tau}$ with $\{\boldsymbol{\pi}_\tau\}$ (see Definition 2), we obtain family of random variables $\{\boldsymbol{\mu}_\tau\}$ (on $\mathcal{I}^l$) that take values on the unit interval. By Proposition 4, the marginal distribution of each $\boldsymbol{\mu}_\tau$ on $\mathcal{I}^l$ is the same as the (kernel density smoothed) univariate distribution of $\boldsymbol{\mu}_\tau$ on $\mathcal{I}$. According to Proposition 8, each $\{\boldsymbol{\mu}_\tau\}$ defined as a univariate function

on $\mathcal{I}$ is uniformly distributed on the unit interval. Therefore, the $\{\boldsymbol{\mu}_\tau\}$ are marginally identically distributed, however, they are not independent. Nevertheless, to the extent that the $\{\boldsymbol{\mu}_\tau\}$ can be decomposed into the sum of two random variables such that the τ-dependent term (taken as a collection) are mutually independent, we now show that $\boldsymbol{\psi}$ and $\boldsymbol{\omega}$ are independent.

Proposition 9 *Let $\{\boldsymbol{\mu}_\tau\}$ be a collection of identically distributed random variables. Assume there exists a continuous random variable $\boldsymbol{\alpha}$ on $\mathcal{I}$ and a family of random variables $\{\boldsymbol{\epsilon}_\tau\}_{\tau\in L}$ such that*

$$\boldsymbol{\mu}_\tau = \boldsymbol{\epsilon}_\tau + \boldsymbol{\alpha} \tag{44}$$

for all $\tau \in L$, and such that $\{\boldsymbol{\epsilon}_\tau\}$ are are mutually independent and independent of $\boldsymbol{\alpha}$. The random variables $\boldsymbol{\psi}$ and $\boldsymbol{\omega}$, as defined in Eqs. 5 and 6, are independent.

Proof Since the $\{\boldsymbol{\mu}_\tau\}_{\tau\in L}$ are identically distributed it follows from Eq. 44 that the variables $\{\boldsymbol{\epsilon}_\tau\}_{\tau\in L}$ are identically distributed. We further observe that

$$\boldsymbol{\psi} = \underset{\tau\in L}{\operatorname{argmax}} \left(\boldsymbol{\epsilon}_\tau\right). \tag{45}$$

Hence, $\boldsymbol{\psi}$ is independent of $\boldsymbol{\alpha}$. Let us now define a continuous random variable $\boldsymbol{\beta}$ by

$$\boldsymbol{\beta} \equiv \max_{\tau\in L} \left(\boldsymbol{\epsilon}_\tau\right). \tag{46}$$

Since the $\{\boldsymbol{\epsilon}_\tau\}_{\tau\in L}$ are independent and identically distributed, it also follows that $\boldsymbol{\psi}$ is independent of $\boldsymbol{\beta}$. Thus, $\boldsymbol{\psi}$ is independent of $\boldsymbol{\alpha}$ and $\boldsymbol{\beta}$. Since the $\{\boldsymbol{\epsilon}_\tau\}$ are independent of $\boldsymbol{\alpha}$, it follows from Eq. 46, that $\boldsymbol{\alpha}$ is independent of $\boldsymbol{\beta}$. Therefore $\boldsymbol{\psi}$ is independent of $\boldsymbol{\alpha} + \boldsymbol{\beta}$. Now, from Eqs. 6, 44, and 46, we obtain

$$\boldsymbol{\omega} = 1 - (\boldsymbol{\beta} + \boldsymbol{\alpha}). \tag{47}$$

It follows immediately that $\boldsymbol{\omega}$ is independent of $\boldsymbol{\psi}$. ∎

The assumption that the residuals $\{\boldsymbol{\epsilon}_\tau\}_{\tau\in L}$ are independent of the bias $\boldsymbol{\alpha}$ is much weaker than assuming that the $\{\boldsymbol{\mu}_\tau\}_{\tau\in L}$ are independent. The validity of the assumption embodied in Eq. 44 for the particular case of the set H of non-interacting gene pairs has been demonstrated empirically by observing that the distribution $\mathcal{F}_{\boldsymbol{\omega}}\left(\boldsymbol{\omega}|\psi\right)$ is (to a reasonable approximation) uniform, and equivalent to the marginal distribution of $\mathcal{F}_{\boldsymbol{\omega}}\left(\boldsymbol{\omega}\right)$ (see Figure S17, Supplementary Information). A common situation involving the decomposition

of Eq. 44 is the scenario where $\boldsymbol{\alpha}$ is defined as the average (over τ) of $\boldsymbol{\mu}_\tau$; in that case, the premise of Proposition 9 would be that the τ-dependent variation of $\boldsymbol{\mu}_\tau$ about the mean, is independent of the mean value.

We now prove that given two real-valued random variables $\boldsymbol{\alpha}$ and $\boldsymbol{\beta}$ and a differentiable function $f : \mathbb{R} \to \mathbb{R}$, $\boldsymbol{\alpha}$ (assumed to have countable solutions to $f(\beta) = \gamma$ for each possible γ) is independent of $f(\boldsymbol{\beta})$.

Proposition 10 *Let $\boldsymbol{\alpha}$ and $\boldsymbol{\beta}$ be independent real-valued random variables, and let $f : \mathbb{R} \to \mathbb{R}$ be a differentiable function. Then $\boldsymbol{\alpha}$ and $f(\boldsymbol{\beta})$ are independent.*

Proof We denote by $\mathcal{D}_{\boldsymbol{\alpha}\beta}$ the joint probability density of outcomes α and β. Since $\boldsymbol{\alpha}$ and $\boldsymbol{\beta}$ are independent, the joint probability density for outcomes α and β satisfies

$$\mathcal{D}_{\boldsymbol{\alpha}\beta}(\alpha, \beta) = \mathcal{D}_{\boldsymbol{\alpha}}(\alpha)\mathcal{D}_{\boldsymbol{\beta}}(\beta),$$

where $\mathcal{D}_{\boldsymbol{\alpha}}$ is the marginal probability density for the outcome α, and $\mathcal{D}_{\boldsymbol{\beta}}$ is the marginal probability density for the outcome β. A formula for computing the probability density function (PDF) of a function of a random variable in terms of the PDF of the random variable, is given in [10]. Using this formula, the joint probability density of outcomes α and γ is:

$$\mathcal{D}_{\boldsymbol{\alpha}\gamma}(\alpha, \gamma) = \sum_{i=1}^{n(\gamma)} \frac{\mathcal{D}_{\boldsymbol{\alpha}\beta}(\alpha, \beta_i)}{f'(\beta_i)},$$

from which it follows that $\boldsymbol{\alpha}$ and $\boldsymbol{\gamma}$ are independent. Note that monotonicity of f is not required in this proof, which is important for its application to $R(\tau)$ in the significance test (see Section 2). ▮

References

[1] Fisher RA (1925) Statistical methods for research workers. London: Oliver & Loyd.

[2] Monk NAM (2003) Oscillatory expression of Hes1, p53, and NF-κB driven by transcriptional time delay. Current Biol 13:1409–13.

[3] Yu J, Xiao J, Ren X, Lao K, Xie S (2006) Probing gene expression in live cells, one protein molecule at a time. Science 311:1600–3.

[4] Deisseroth K, Heist EK, Tsien RW (1998) Translocation of calmodulin to the nucleus supports CREB phosphorylation in hippocampal neurons. Nature 392:198–202.

[5] Zak DE (2005) Structured modeling of mammalian transcriptional networks. Ph.D. thesis, University of Delaware, Newark, DE.

[6] Adelman K, Porta AL, Santangelo TJ, Lis JT, Roberts JW, et al. (2002) Single molecule analysis of RNA polymerase elongation reveals uniform kinetic behavior. Proc Natl Acad Sci U S A 99:13538–43.

[7] Roussel MR, Zhu R (2006) Stochastic kinetics description of a simple transcription model. Bull Math Biol 68:1681–1713.

[8] Barrio M, Burrage K, Leier A, Tian T (2006) Oscillatory regulation of Hes1: discrete stochastic delay modelling and simulation. PLoS Computational Biology 2:e117.

[9] Sklar A (1959) Fonctions de répartition à n dimensions et leures marges. Publications de l'Institut de Statistique de L'Université de Paris 8:229–31.

[10] Papoulis A (1991) Probability, random variables, and stochastic processes. New York: McGraw-Hill.
