# Supplementary figures and images for: Uncovering a Macrophage Transcriptional Program by Integrating Evidence from Motif Scanning and Expression Dynamics
Source: PLoS Comput Biol. 2008 Mar 21;4(3):e1000021. doi: 10.1371/journal.pcbi.1000021 (PMC2265556; doi:10.1371/journal.pcbi.1000021)

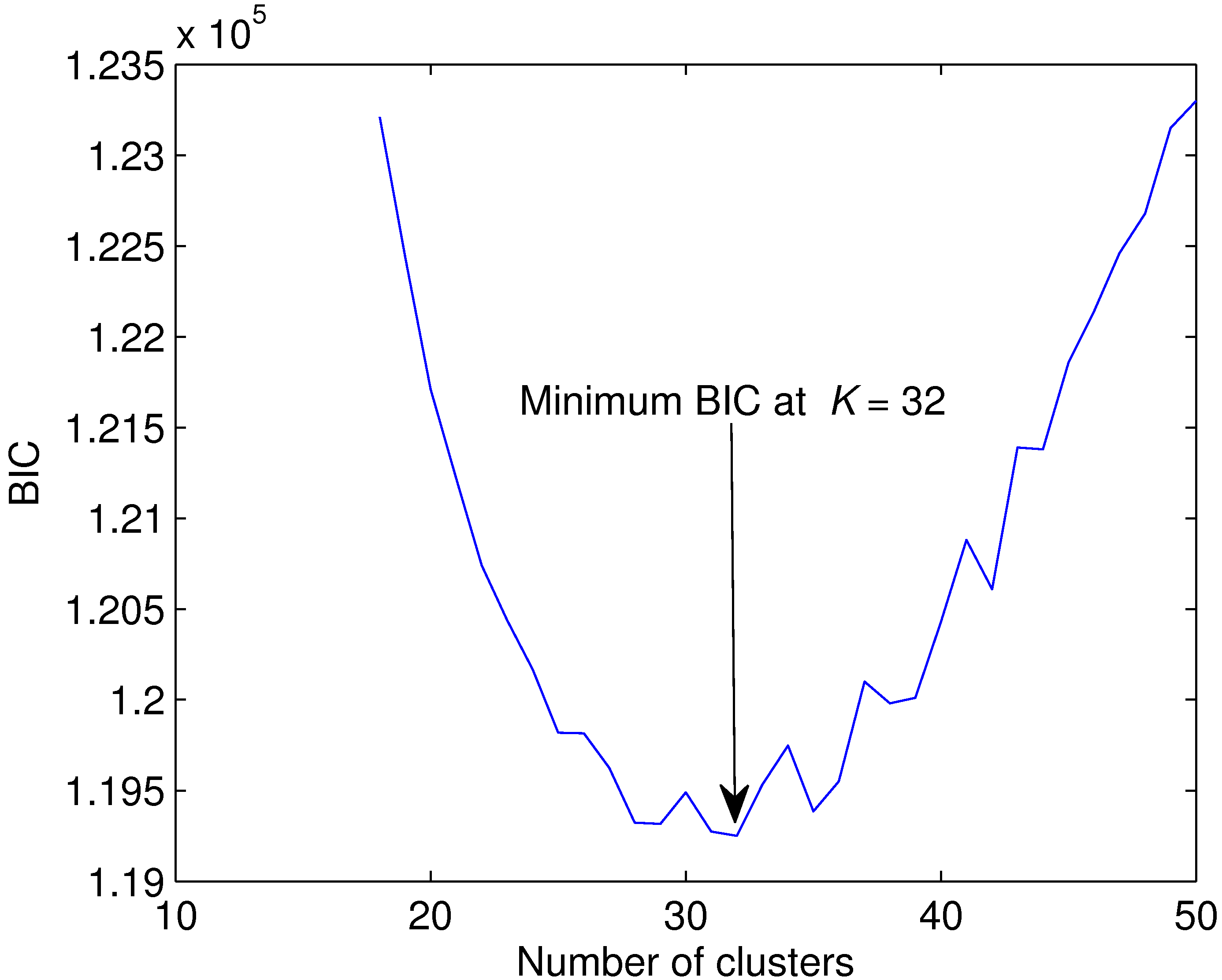

Supplement: Figure S1 — The optimal number of clusters was determined using the Bayesian Information Criterion (BIC). The horizontal axis indicates the number of clusters K used for K-means clustering. The cluster analysis was repeated for K varying between 18 and 50, with the BIC computed for each number of clusters. The optimal number of clusters, for which the BIC is minimized, was found to be K = 32 (see Materials and Methods, Expression Clustering). (0.15 MB TIF) [file pcbi.1000021.s002.tif]

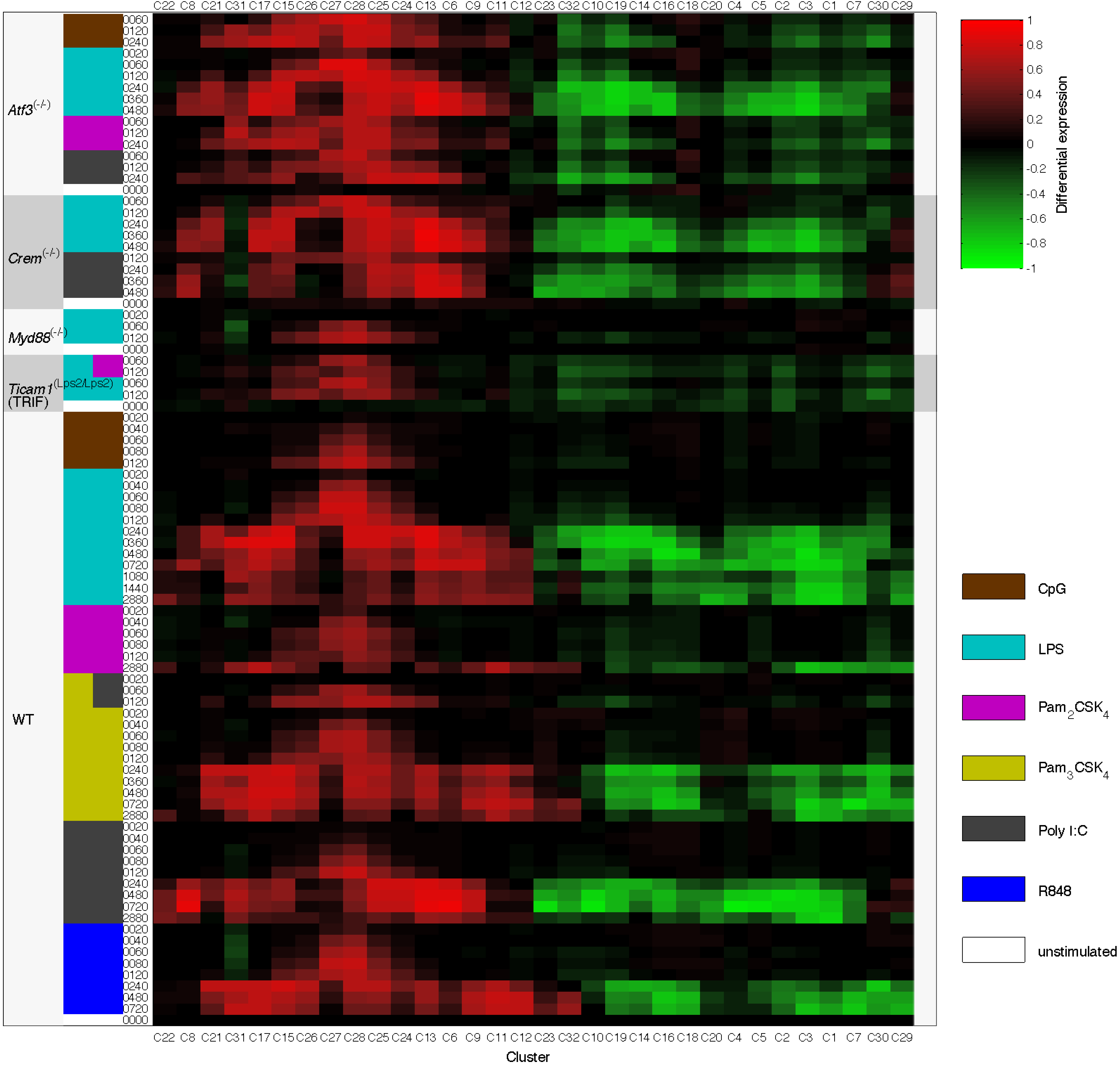

Supplement: Figure S2 — Differential expression profiles of gene clusters, in TLR-stimulated macrophages, across all microarray expression experiments. Each row represents an experiment (a specific combination of strain, stimulus, and time point), and each column represents a cluster. Clusters are displayed in the order that minimizes the sum of pairwise distances between adjacent clusters (see Materials and Methods, Expression Clustering). Each colored rectangle within the heat-map indicates the centroid of the expression levels for genes within the indicated cluster, for the indicated experiment. The differential expression level (SDR, see Equation 1) is indicated in red/green color, and varies between -1 (bright green) and 1 (bright red), with 0 (black) indicating no change from the expression level in the unstimulated wild-type macrophage. The shaded light gray/charcoal regions in the far left column indicate the genotype. The color-coding in the second-to-left column indicates the stimulus (color code legend in lower right; and see Table S2 for the concentrations). The four-digit numbers to the right of the color-code column, indicate the elapsed time (min) post-stimulation, for each experiment. (1.38 MB TIF) [file pcbi.1000021.s003.tif]

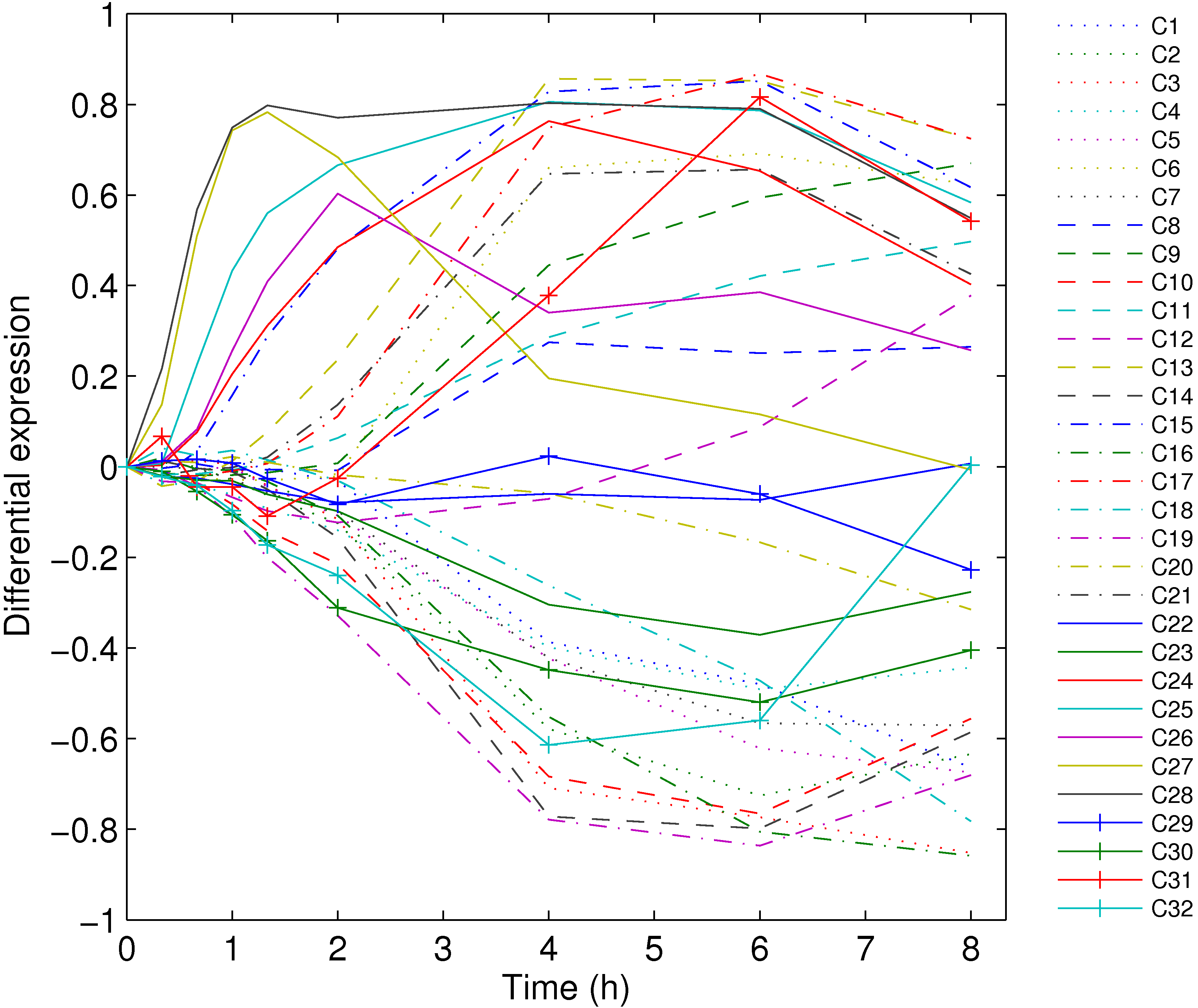

Supplement: Figure S3 — Cluster-median differential expression profiles in wild-type macrophages stimulated with LPS show a diversity of time scales. Each data point shown is the median of the SDR-transformed (see Equation 1) differential expression levels of the genes within the indicated cluster, at the indicated time after stimulation. (0.34 MB TIF) [file pcbi.1000021.s004.tif]

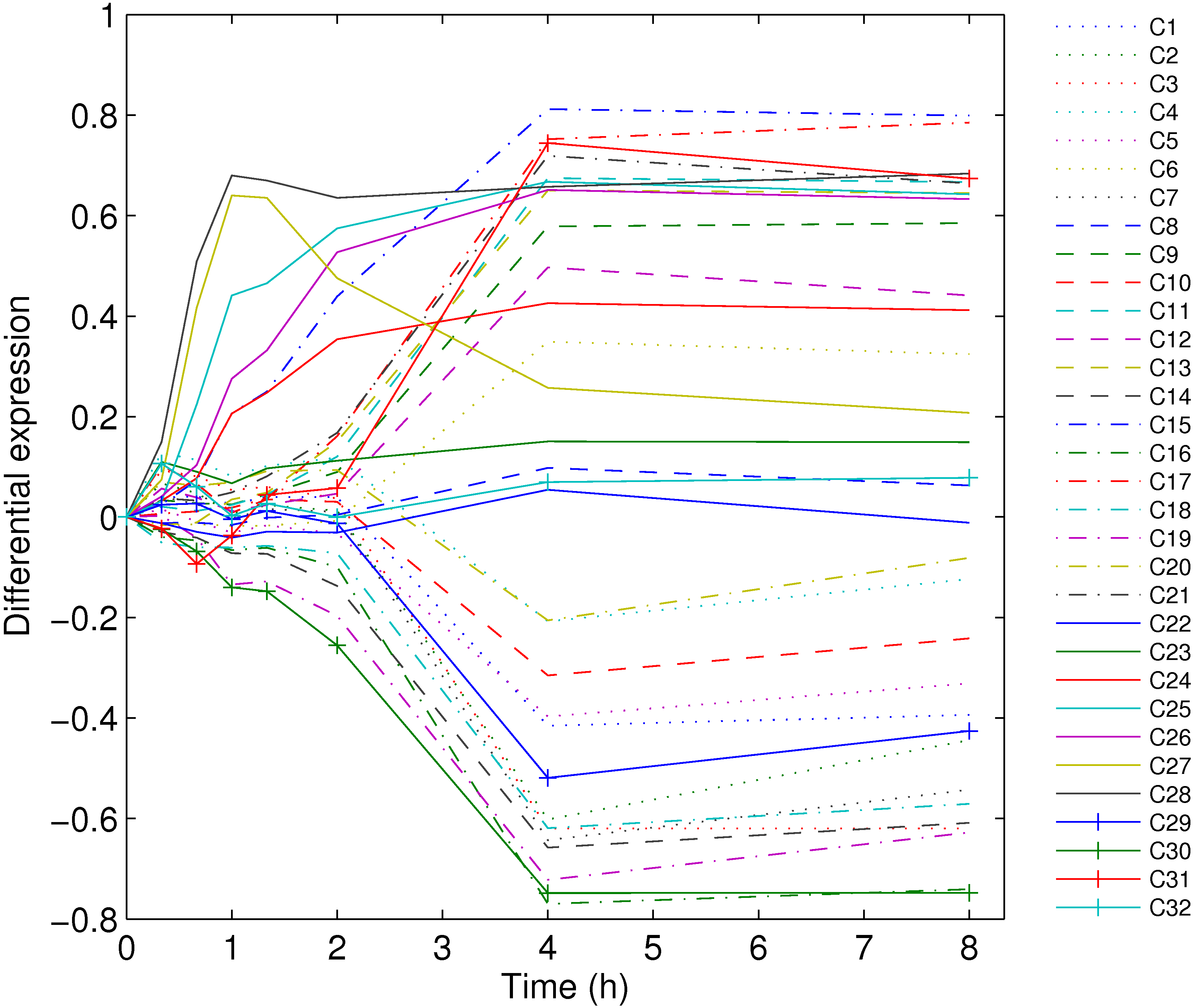

Supplement: Figure S4 — Cluster-median differential expression profiles in wild-type macrophages stimulated with Pam3CSK4 show a diversity of time scales. Each data point shown is the median of the SDR-transformed (see Equation 1) differential expression levels of the genes within the indicated cluster, at the indicated time after stimulation. Cluster C26 shows sustained activation under this stimulus, as opposed to the case of stimulation with LPS (see Figure S3). (0.32 MB TIF) [file pcbi.1000021.s005.tif]

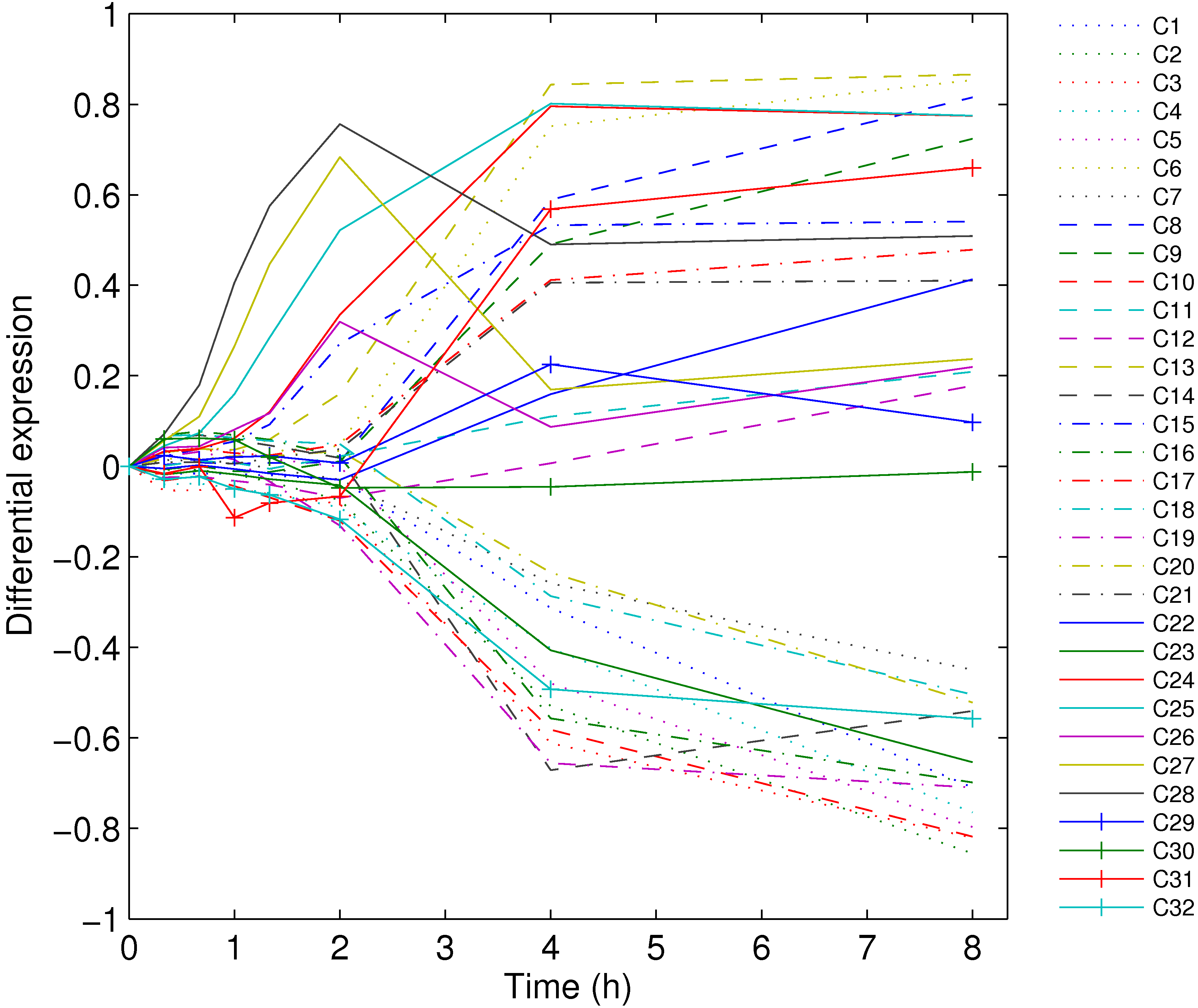

Supplement: Figure S5 — Cluster-median differential expression profiles in wild-type macrophages stimulated with poly I:C show a diversity of time scales. Each data point shown is the median of the SDR-transformed (see Equation 1) differential expression levels of the genes within the indicated cluster, at the indicated time after stimulation. The core response Clusters C27 and C28 induce later in this time-course experiment than in the case of stimulation with LPS (Figure S3). (0.32 MB TIF) [file pcbi.1000021.s006.tif]

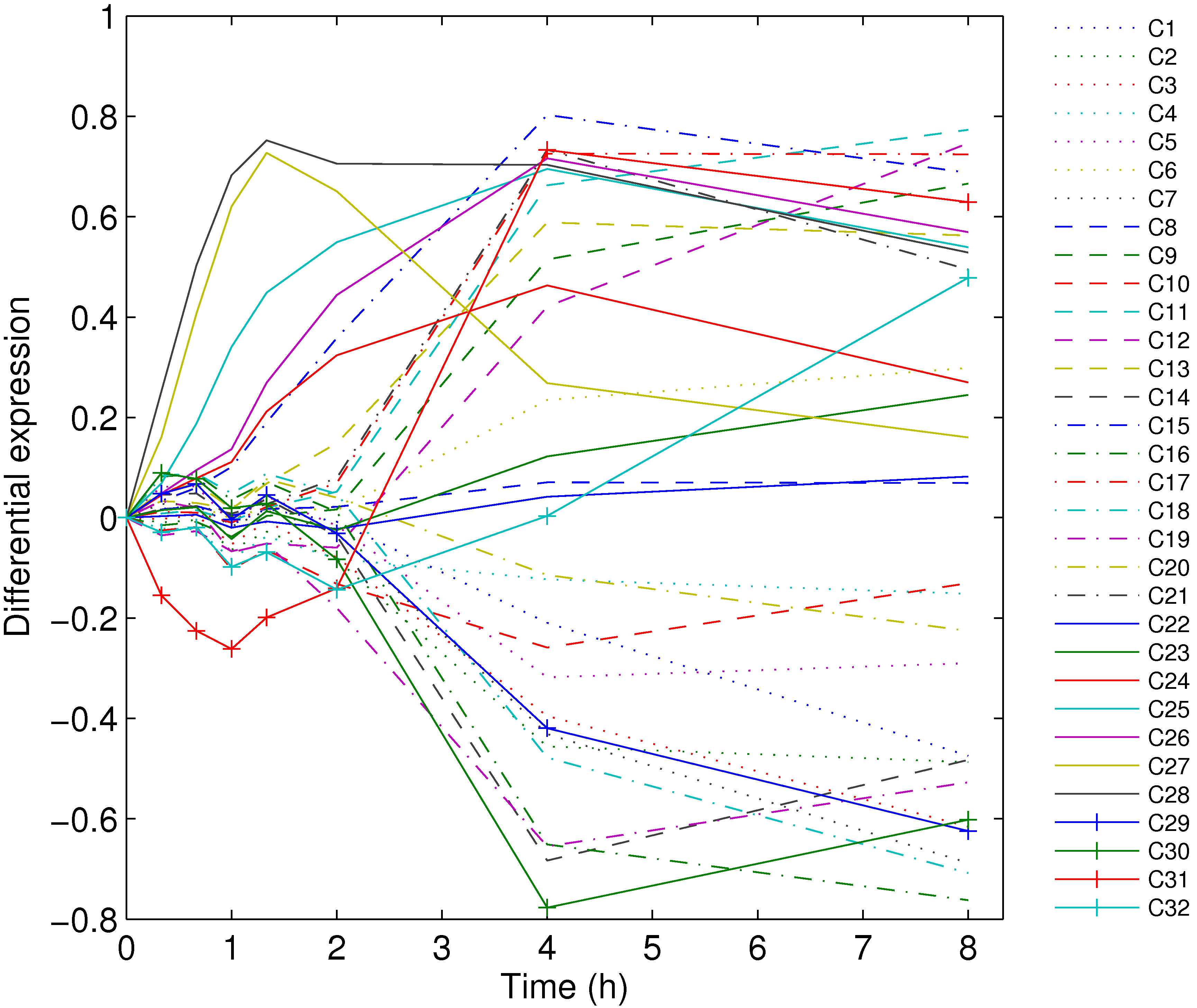

Supplement: Figure S6 — Cluster-median differential expression profiles of wild-type macrophages stimulated with R848 show a diversity of time scales. Each data point shown is the median of the SDR-transformed (see Equation 1) differential expression levels of the genes within the indicated cluster, at the indicated time after stimulation. Cluster C26 shows sustained activation under this stimulus, as opposed to the case of stimulation with LPS (see Figure S3). (0.34 MB TIF) [file pcbi.1000021.s007.tif]

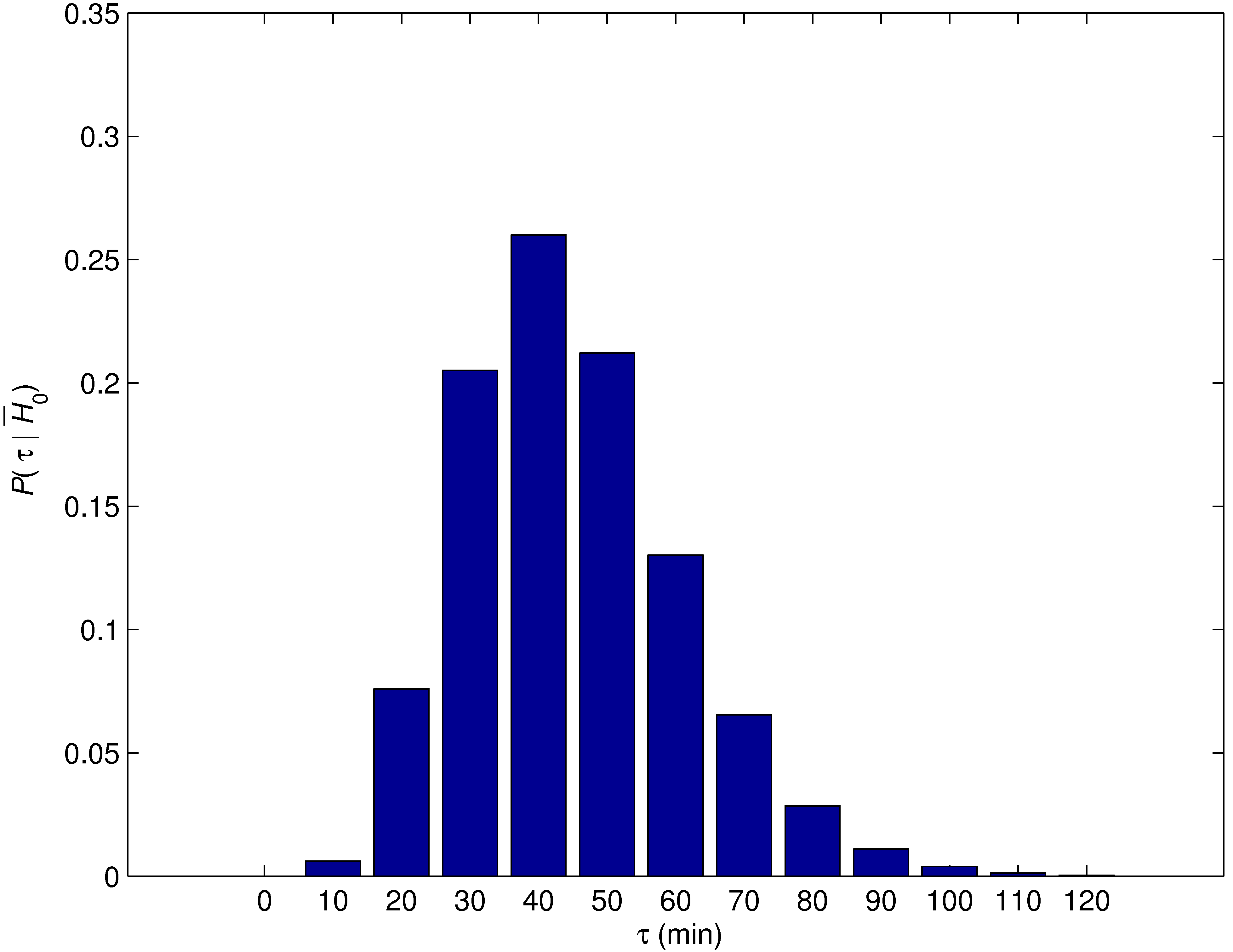

Supplement: Figure S7 — Discretized prior probability distribution P(τ|H0) of observing an optimal time-lag τ, for a gene pair that have a transcriptional regulatory interaction. Here, the symbol ∼H0 denotes the complement of the null hypothesis, i.e., that there is a transcriptional regulatory interaction (this is denoted by an overbar in the main text and in the supporting text). The symbol τ denotes the optimal time lag. For a discussion and derivation of the prior probability distribution of transcriptional time lags, see Materials and Methods (Constructing the Prior Distribution of Time Lags) and Text S1 (Section 3). (0.26 MB TIF) [file pcbi.1000021.s008.tif]

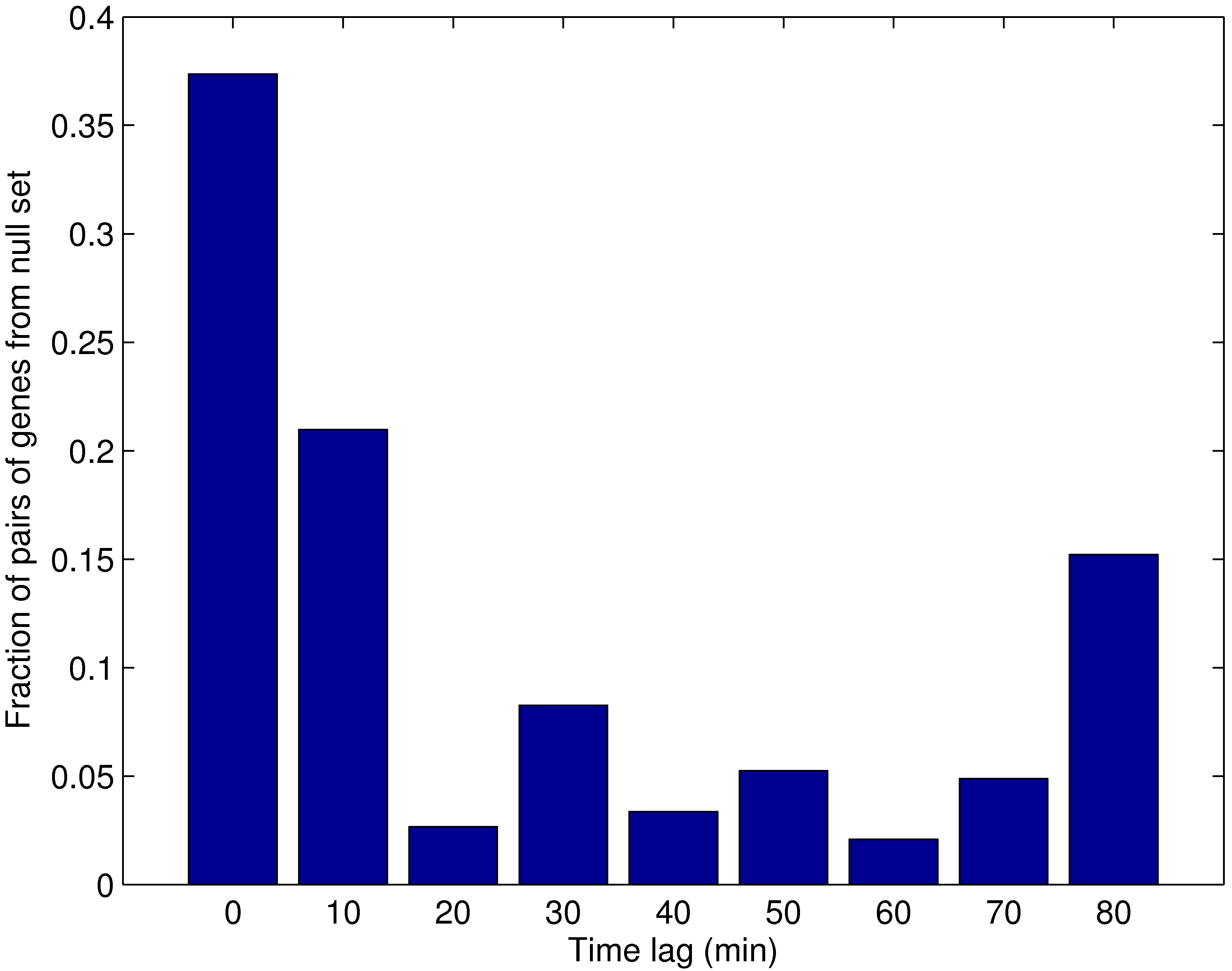

Supplement: Figure S8 — Histogram of time lag values that maximize the absolute time-lagged correlation coefficient, for randomly drawn pairs of non-transcription factor genes. The non-uniformity of the histogram (the highest counts appear at high and low values of the time lag) shows the inherent bias in the standard method of selecting the optimal time lag, i.e., maximizing the absolute lagged correlation coefficient. Time-lagged correlations could not be reliably estimated for time lags greater than 80 min, due to limited effective sample size for higher time lags (see Materials and Methods, Constructing the Prior Distribution of Time Lags). (0.22 MB TIF) [file pcbi.1000021.s009.tif]

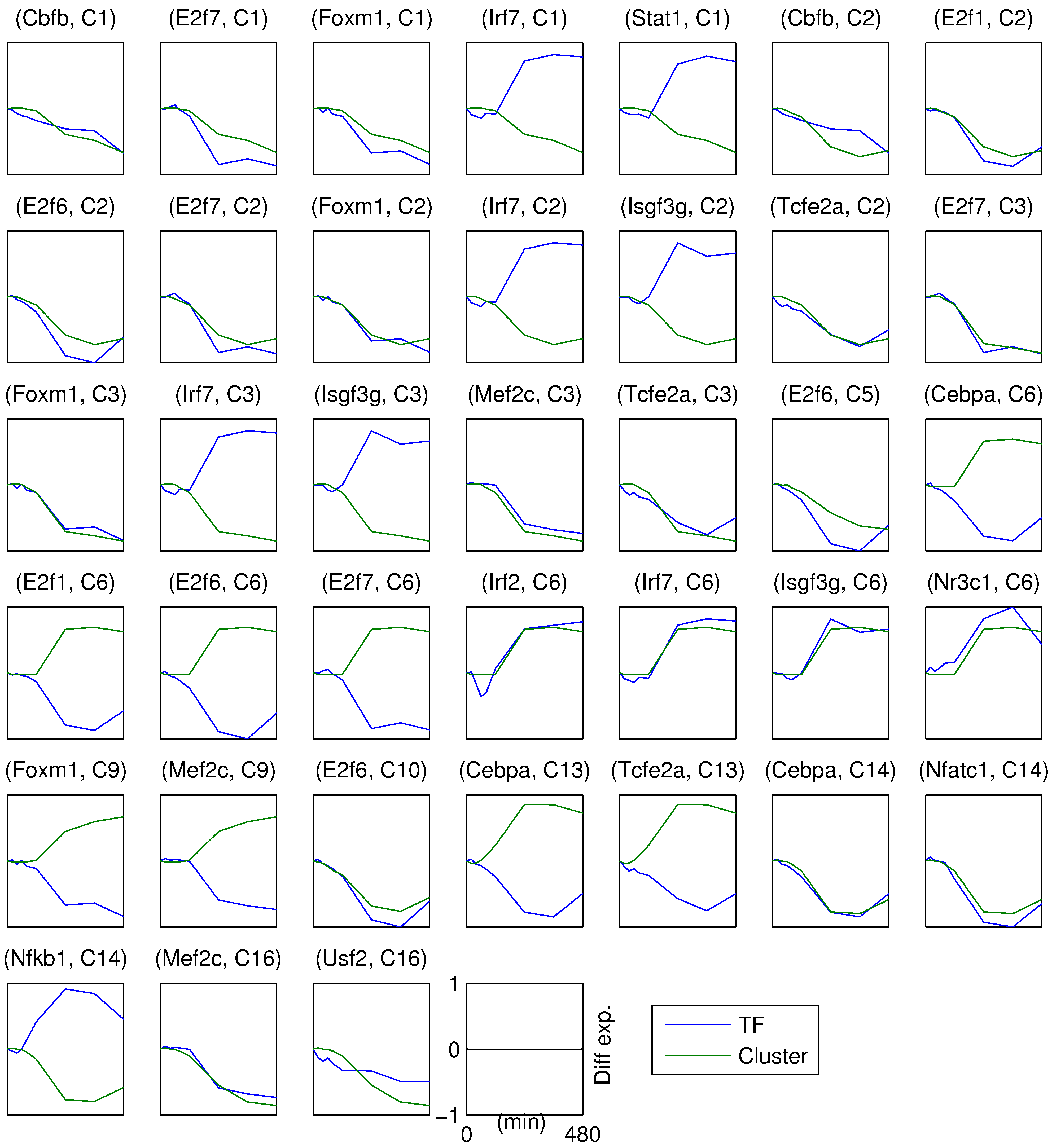

Supplement: Figure S9 — Differential expression levels (SDR, see Equation 1) in wild-type macrophages stimulated with LPS, for 38 pairs of transcription factor genes and gene clusters. The pairs all show high-significance time-lagged correlation based on the significance criterion P exp ≤ 5×10^-3^, and all satisfy the minimum average time lag criterion <θ> ≥ 10 min. Differential expression levels are relative to wild-type unstimulated macrophages, with positive/negative values indicating upregulation/downregulation. The names of the TF gene and the correlated cluster are shown above each plot. The cluster expression level, shown in green, is the centroid from the K -means clustering algorithm (see Materials and Methods, Expression Clustering). Of the pairs, 23 have a positive time-lagged correlation coefficient, and 15 have a negative time-lagged correlation coefficient. (0.52 MB TIF) [file pcbi.1000021.s010.tif]

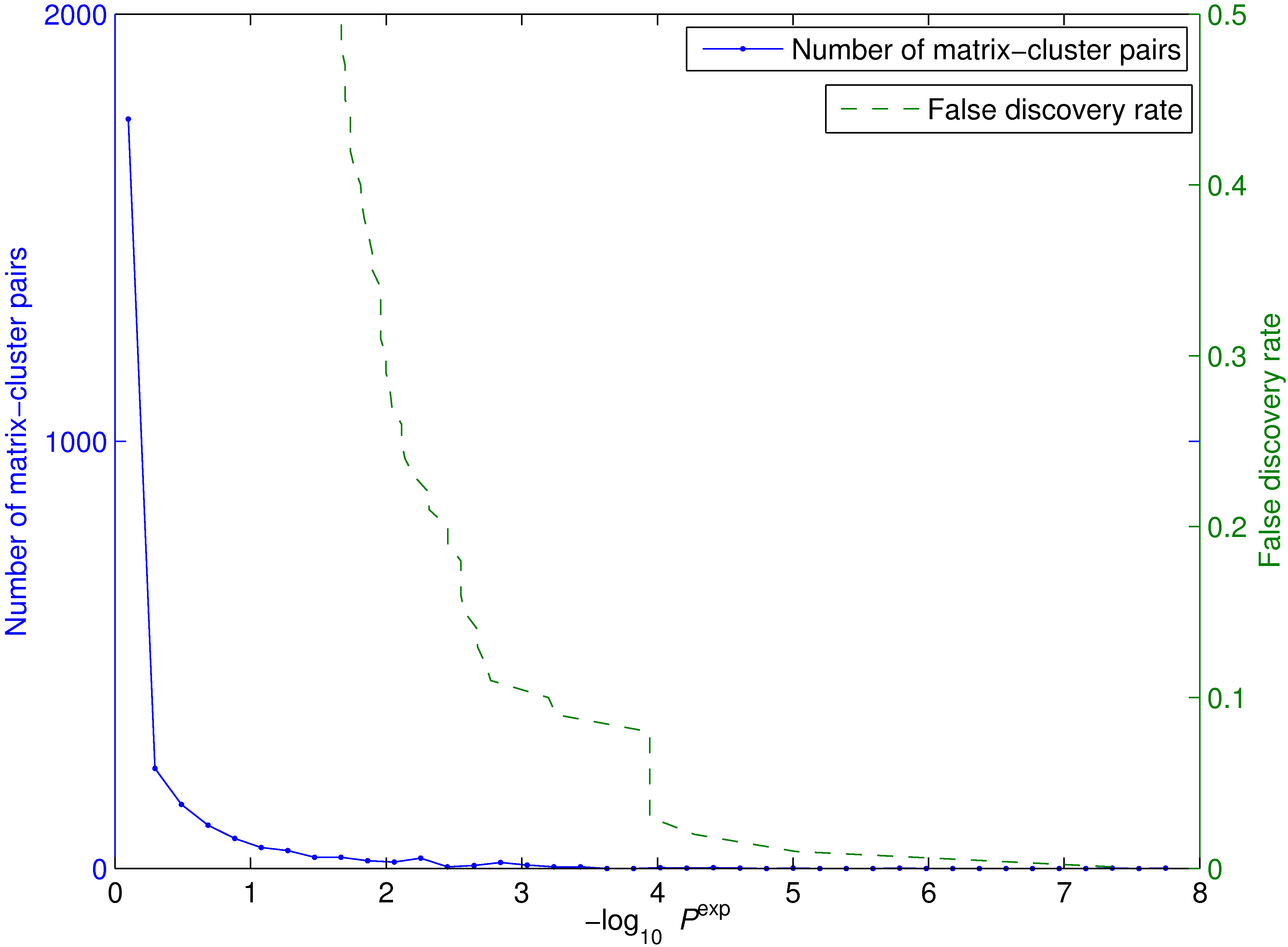

Supplement: Figure S10 — Combined plot showing (i) the histogram of -log10 P exp values for the significance of the time-lagged correlation; and (ii) the estimated false discovery rate, as a function of the -log10 P exp value. The P exp values were computed for all possible pairs of (f,C) of transcription factor gene f and coexpressed gene cluster C. The histogram was generated using 40 bins. (0.27 MB TIF) [file pcbi.1000021.s011.tif]

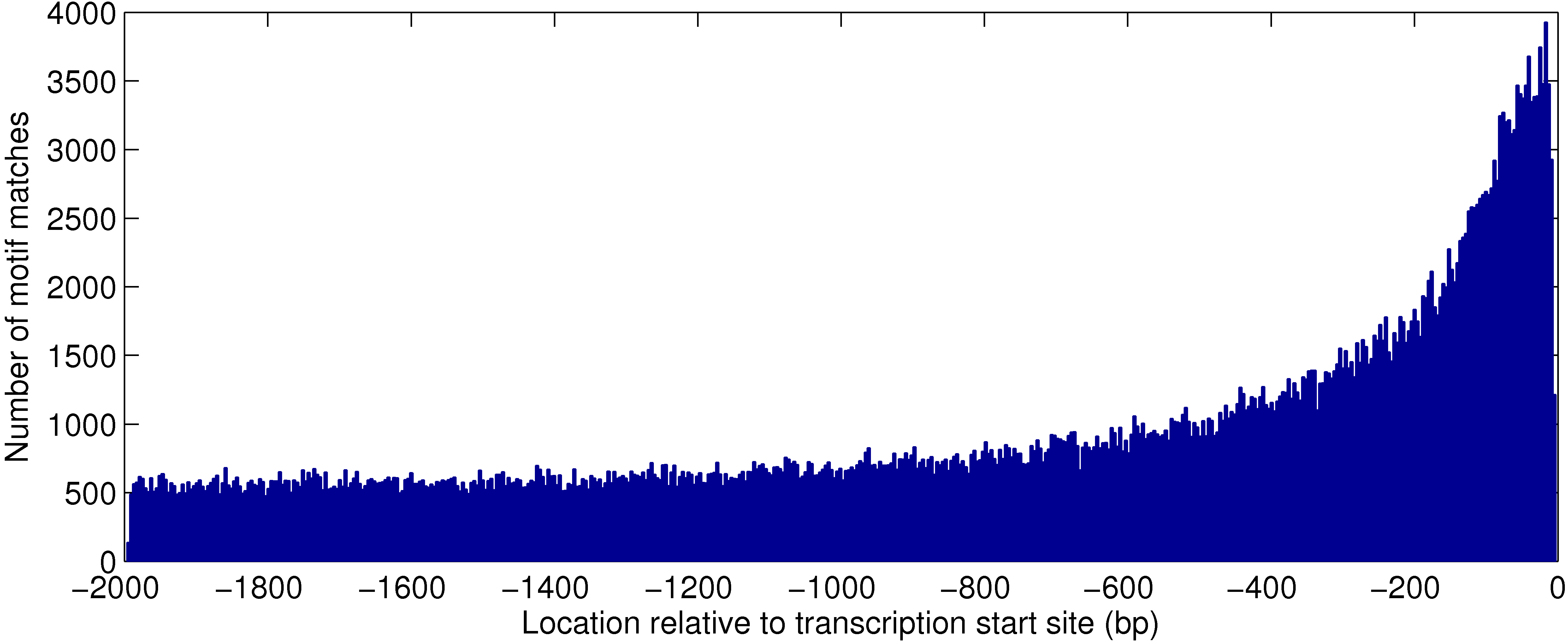

Supplement: Figure S11 — Histogram of positions of transcription factor binding site motif matches relative to transcription start site. The median distance from the transcription start site is 537 bp. The density of motif matches can be seen to peak at −20 bp relative to the start site. (0.25 MB TIF) [file pcbi.1000021.s012.tif]

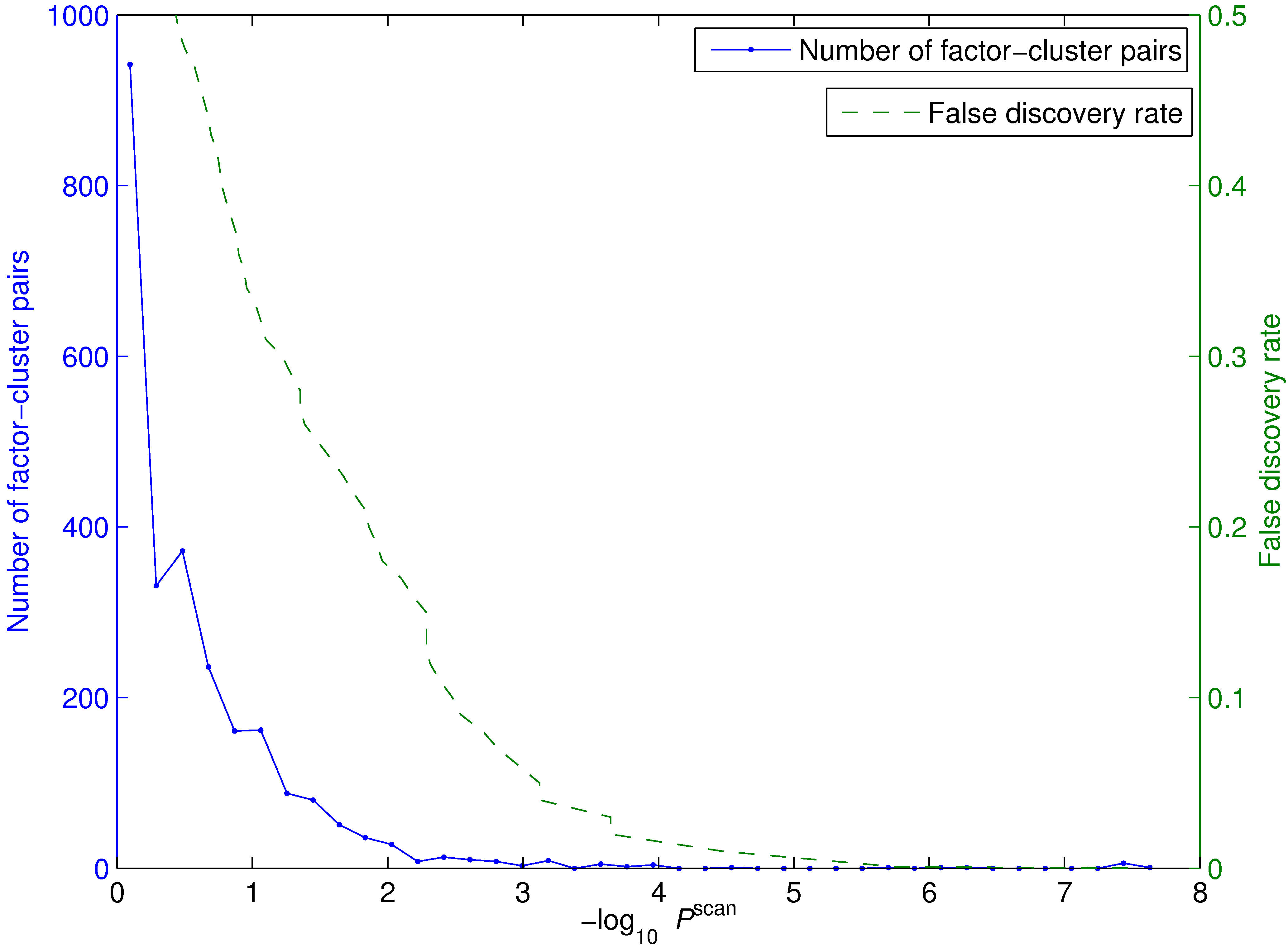

Supplement: Figure S12 — Combined plot showing (i) the histogram of -log10 P scan values for enrichment of TFBS motifs within co-expressed gene clusters; and (ii) the estimated false discovery rate as a function of the -log10 P scan value. The P scan values were computed for all possible pairs pairs (f,C) of transcription factor gene f and cluster C, using the position-weight matrix associated with f that had the smallest enrichment P value for the promoters of the genes in cluster C. The histogram was generated using 40 bins. (0.28 MB TIF) [file pcbi.1000021.s013.tif]

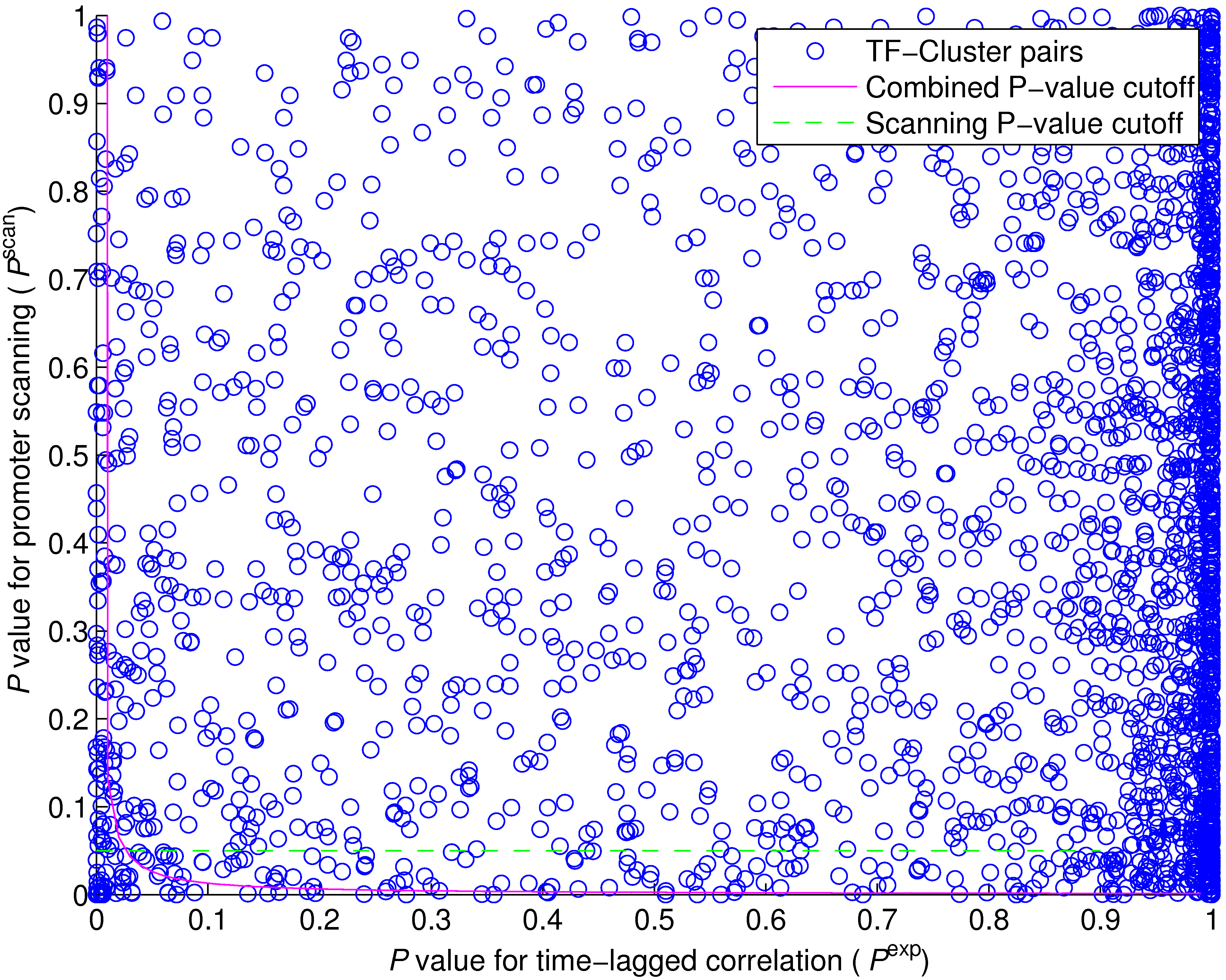

Supplement: Figure S13 — Integrating the two sources of evidence using Fisher's method. Each blue circle represents a unique (TF,cluster) pair. The solid line indicates the cutoff for the combined P value, at FDR = 0.1. Data points to the lower left of the line have a P comb value smaller than the cutoff (see Materials and Methods, Network Inference). The dotted green line indicates the cutoff for the promoter scanning-based P value, P scan = 0.05. Pairs that fall below the green dotted line and to the lower-left of the solid magenta line and for which the average time lag <θ> ≥ 10 min, were included in the final network. (0.59 MB TIF) [file pcbi.1000021.s014.tif]

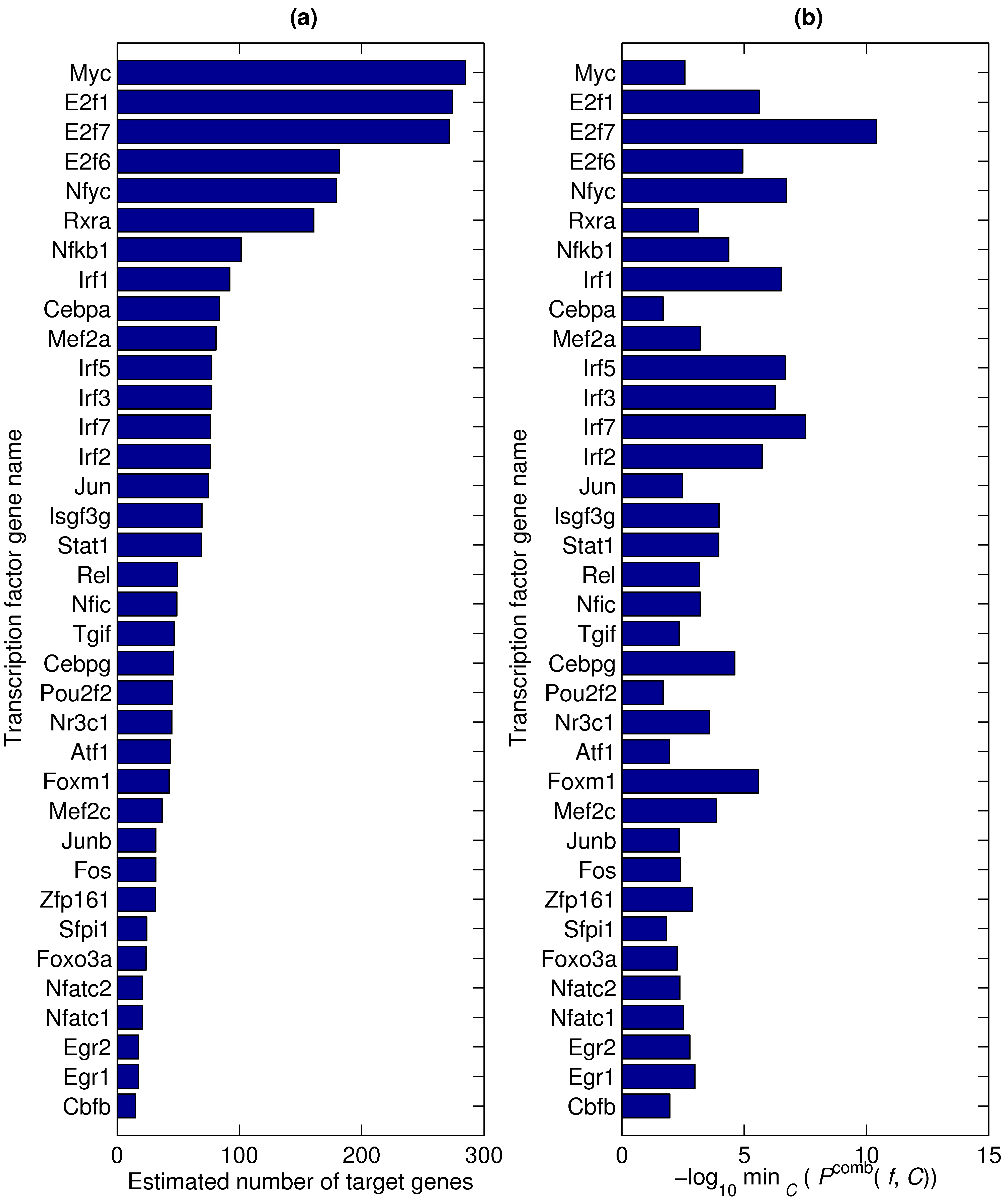

Supplement: Figure S14 — The set of transcription factor genes has a 20-fold variation in out-degree (number of target genes), within the transcriptional network. (a) Estimated out degree of transcription factor genes. The out degree of a transcription factor gene is the number of genes estimated to be regulated by the transcription factor(s) associated with that TF gene (i.e., of which that TF gene is a component). For each gene cluster with which a TF gene was associated, the number of genes within the cluster for which a motif match was found (corresponding to the TF gene), was tabulated. The number of target genes was summed over all clusters with which the TF was associated, based on the combined expression and promoter scanning data (see Materials and Methods, Network Inference). Among the 36 TF genes in the network, the estimated out degree had a median of 49, and a maximum value of 285. (b) Estimated significance of the association of the TF gene in the network. For each TF gene f implicated in the network, the minimum P value P comb(f,C) of association with any cluster C, was used as a measure of the overall significance of the association of TF gene in the transcriptional network. Transcription factor genes are displayed in decreasing order of estimated out degree (number of target genes). Transcription factors associated with larger clusters are seen to correlate with higher significances in the network, as a consequence of the sample size-dependence of the statistical tests used for the motif scanning and expression dynamics evidences. (0.51 MB TIF) [file pcbi.1000021.s015.tif]

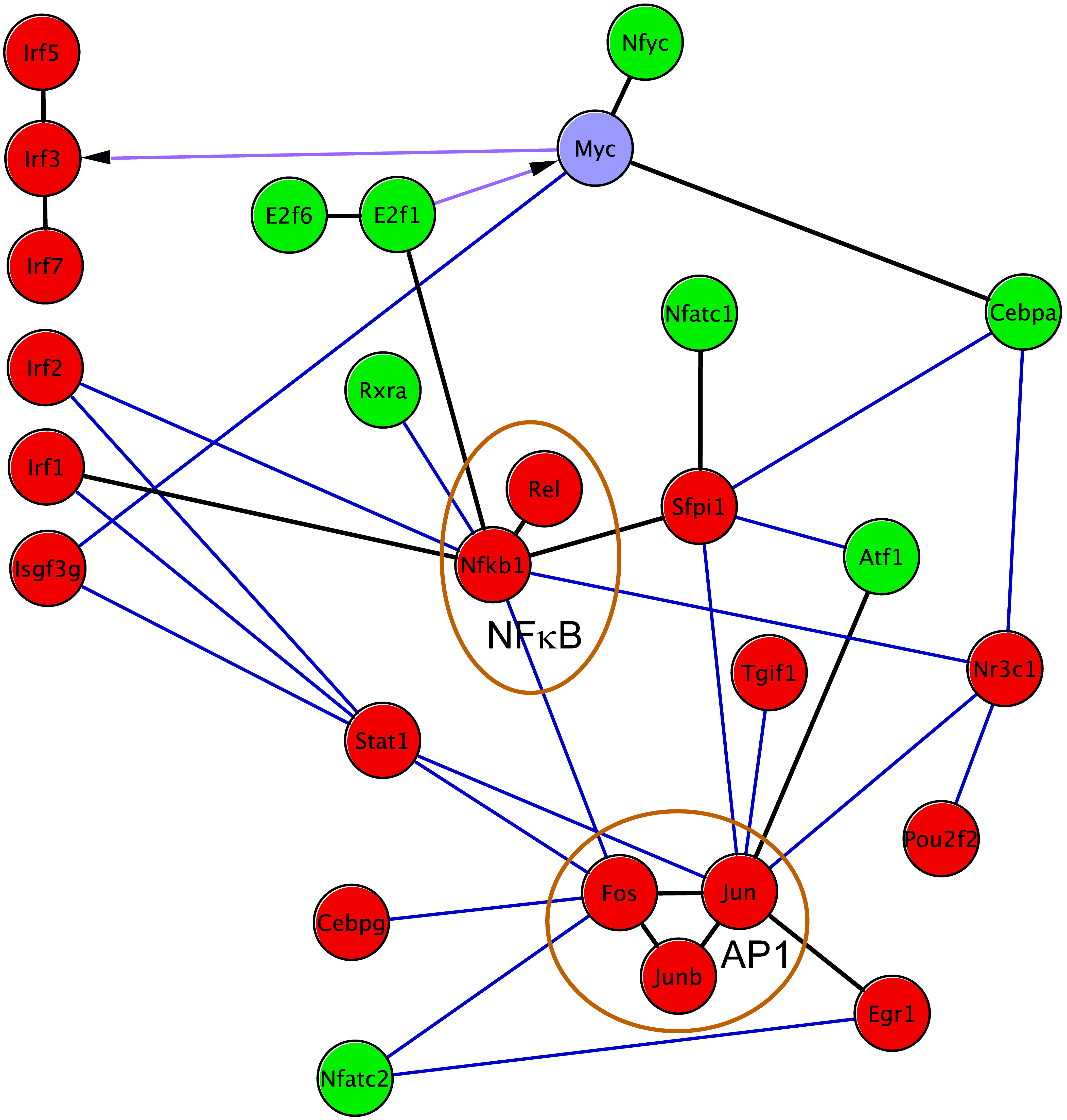

Supplement: Figure S15 — Transcription factors involved in macrophage activation are highly interconnected in the protein interaction network, and the interacting TFs co-associate with clusters. Nodes indicate TF genes whose transcript levels are differentially expressed in LPS-stimulated macrophages, and that are associated with the transcriptional network through the combination of scanning- and expression-based evidences. Node labels are gene names. A red node indicates upregulated gene expression under LPS, and green indicates downregulation, and a purple node indicates transient up- and downregulation. A blue arc indicates that the human orthologs of the murine proteins associated with the murine TF genes connected by the arc, have an interaction in the Human Protein Reference Database [68] or in the Biomolecular Interaction Network Database [69]. A thick black arc indicates that the two connected TF genes co-associate with one or more clusters within the network, and share a protein interaction (suggesting a possible transcriptional complex). A purple arrow indicates a known protein-DNA interaction between the source node's human ortholog protein and the promoter of the human ortholog of the gene indicated by the target node. Brown ellipses denote the core transcription factor complexes NFκB and AP1. (0.64 MB TIF) [file pcbi.1000021.s016.tif]

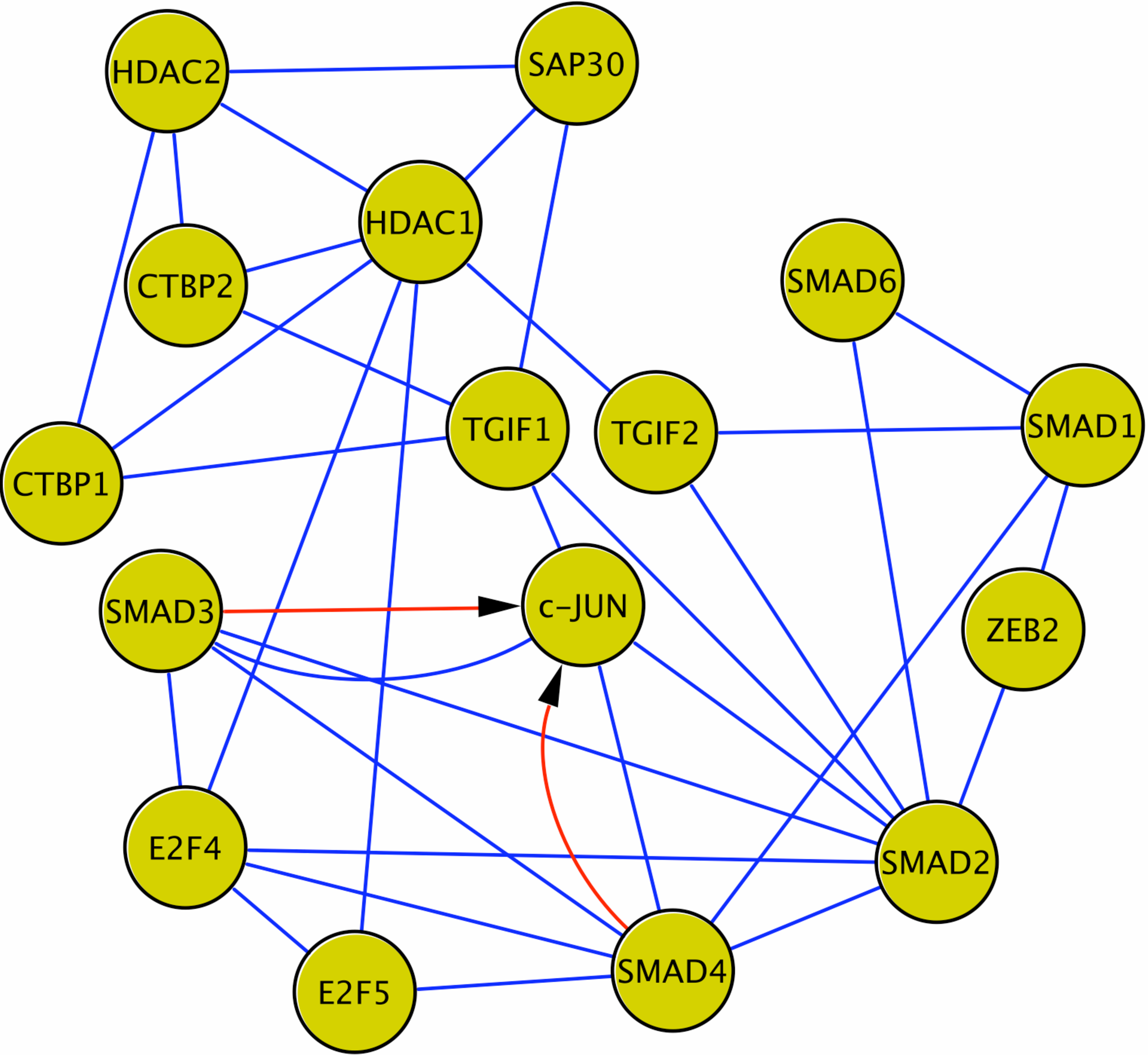

Supplement: Figure S16 — TGIF1 interacts with many members of the SMAD/AP-1 transcription complex. Shown here is a network diagram of 16 proteins that interact with the SMAD family of transcription factors SMAD1/2/3/6, the histone deacetylaces HDAC1/2, and the TG-interacting factors TGIF1/2. Nodes indicate proteins, and a blue line between two nodes indicates that the human orthologs of the two proteins have an interaction, in either the Human Protein Reference Database (HPRD) [68] or in the literature [72],[75]. Red arrows indicate human protein-DNA interactions annotated in the TRANSFAC database [34]. The diagram includes nearest-neighbors of the SMAD, HDAC, and TGIF families in the protein interaction network. Each node shown in the diagram corresponds to a transcript that is likely expressed in murine bone marrow-derived macrophages, based on having an above-threshold microarray intensity within at least one experiment (see Materials and Methods, Probeset Selection). (2.02 MB TIF) [file pcbi.1000021.s017.tif]

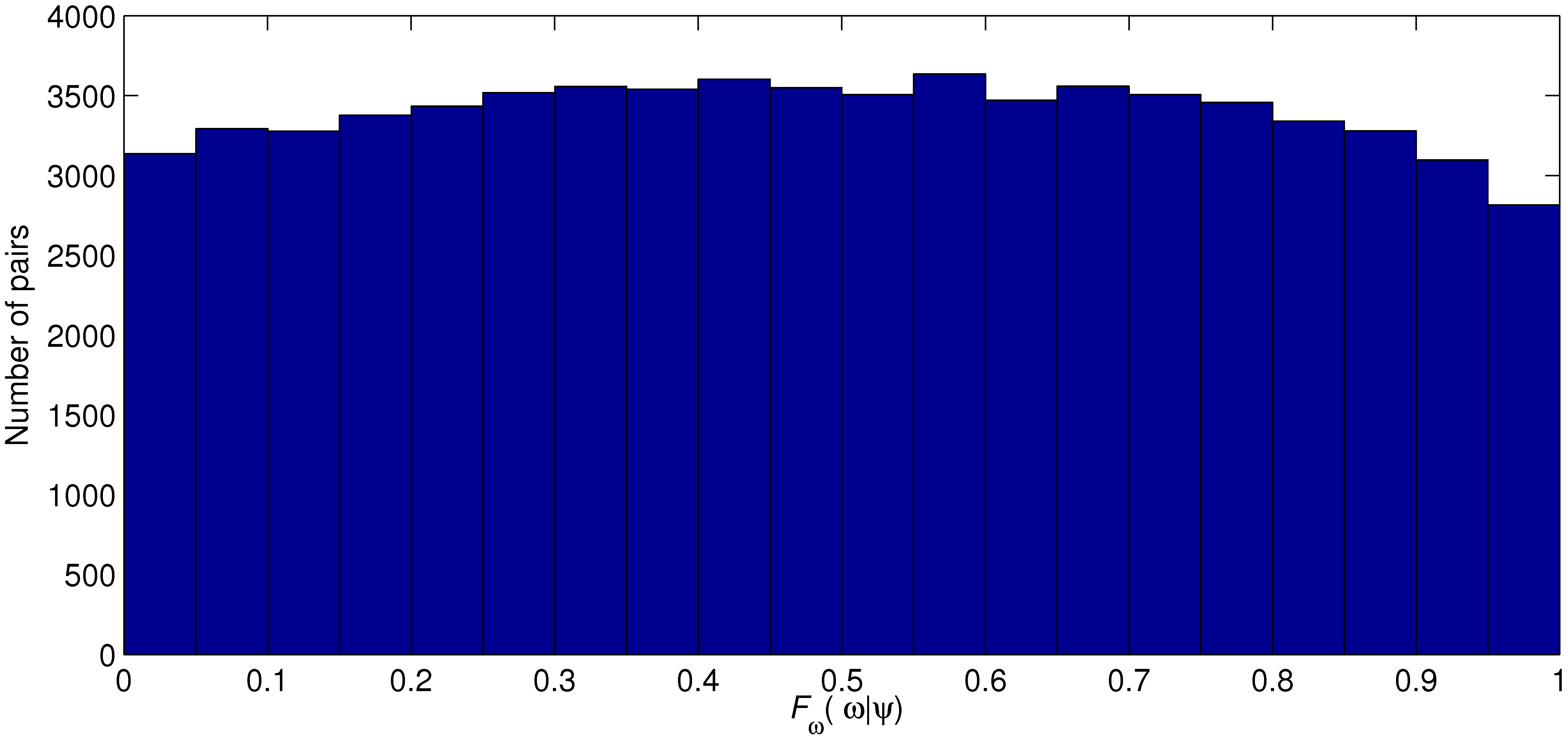

Supplement: Figure S17 — Histogram of the cumulative density function of ω, for the ω values for all sample points with ψ = 80 min. Strict uniformity of this distribution (for each and every outcome ψ = τεL) would imply that ω is totally independent of ω|ψ. Here, conditioning on ψ is seen to not introduce a significant bias in the distribution of ω values (see Supporting Text, Section 2). (0.30 MB TIF) [file pcbi.1000021.s018.tif]
