# Supplementary material for: Uncovering a Macrophage Transcriptional Program by Integrating Evidence from Motif Scanning and Expression Dynamics
Source: PLoS Comput Biol. 2008 Mar 21;4(3):e1000021. doi: 10.1371/journal.pcbi.1000021 (PMC2265556; doi:10.1371/journal.pcbi.1000021)
Supplement: Table S1 — Summary of mutant mouse strains used in this study. Expression data from available mouse strains with mutations of known TLR signaling adapter molecules or known transcriptional regulators were included in the cluster analysis, in order to maximize the diversity of expression patterns in the data set used for clustering. Column 1 is the mutant strain name. Column 2 is the name of the molecule affected by the mutation. Column 3 gives the gene title. Column 4 briefly summarizes the relevance of the molecule in TLR-stimulated macrophages. (0.03 MB DOC) [file pcbi.1000021.s019.doc]

| **Genotype** | **Protein** | **Gene title** | **Protein function and relevance** |
| --- | --- | --- | --- |
| *Atf3*(-/-) [31] | ATF3 | activating transcription factor 3 | transcription factor; negative regulator of TLR4 response [6] |
| *Crem*^(-/-)^ [32] | CREM | cAMP responsive element modulator | transcription factor differentially expressed under TLR 2/3/4 stimulation (see Table S10) |
| *Myd88*^(-/-)^ [5] | MyD88 | myeloid differentiation primary response gene 88 | adapter molecule, TLR2/4/7/8/9 [5] |
| *Ticam1*^(Lps2/Lps2)^ [3] | TRIF | toll-like receptor adapter molecule 1 | adapter molecule, TLR3/4 [4,5] |
