# Supplementary material for: Uncovering a Macrophage Transcriptional Program by Integrating Evidence from Motif Scanning and Expression Dynamics
Source: PLoS Comput Biol. 2008 Mar 21;4(3):e1000021. doi: 10.1371/journal.pcbi.1000021 (PMC2265556; doi:10.1371/journal.pcbi.1000021)
Supplement: Table S2 — Stimuli used for macrophage gene expression experiments. Column 1 indicates the purified TLR agonist. Column 2 gives the description of the agonist. Column 3 indicates the receptor(s) that are stimulated by the agonist. Column 4 indicates the adapter molecule(s) associated with the receptor. Column 5 indicates the concentration used for in vitro stimulation of macrophages. (0.04 MB DOC) [file pcbi.1000021.s020.doc]

| Stimulus | Description | Receptor(s) | Adapter molecule(s) | Concentration |
| --- | --- | --- | --- | --- |
| LPS | lipopolysaccharide | TLR4 | MyD88 / MAL / TRIF / TRAM | 10 ng/mL |
| Pam_2_CSK_4_ | synthetic diacylated lipopeptide | TLR2/6 | MyD88 / MAL | 300 ng/mL |
| Pam_3_CSK_4_ | synthetic triacylated lipopeptide | TLR2/1 | MyD88 / MAL | 300 ng/mL |
| poly I:C | polyriboinosinic polyribocytidylic acid | TLR3 | TRIF | 6 μg/mL |
| R848 | synthetic imidazoquinoline resiquimod | TLR7/8 | MyD88 | 50 μg/mL |
| CpG | unmethylated CpG motif (cytosine and guanine separated by a phosphate) | TLR9 | MyD88 | 10 μg/mL |
