# Supplementary material for: Uncovering a Macrophage Transcriptional Program by Integrating Evidence from Motif Scanning and Expression Dynamics
Source: PLoS Comput Biol. 2008 Mar 21;4(3):e1000021. doi: 10.1371/journal.pcbi.1000021 (PMC2265556; doi:10.1371/journal.pcbi.1000021)
Supplement: Table S3 — List of microarray experiments included in this study. Each row indicates a microarray experiment. Column 1 indicates the mouse strain, with “Wild-type” indicating C57BL/6. Column 2 indicates the stimulus (or combination of stimuli, separated by a slash “/”). Column 3 indicates the elapsed time post stimulation. Column 4 indicates the number of biological replicates combined in the experiment. Column 5 indicates whether the expression measurements for the experiment were used in identifying differentially expressed genes. Column 6 indicates if the experiment was used for the clustering analysis. Column 7 indicates if the experiment was used for time-lagged correlation (TLC) analysis. The alternating shaded pattern for rows is used to visually distinguish between experiments from different genotypes. (0.21 MB DOC) [file pcbi.1000021.s021.doc]

| **Strain** | **Stimulus** | **Time (min)** | **Replicates** | **Used for diff exp test** | **Used for clustering** | **Used for TLC analysis** |
| --- | --- | --- | --- | --- | --- | --- |
| *Atf3*^(-/-)^ | unstimulated | 0 | 3 |  | x | x |
| *Atf3*^(-/-)^ | CpG | 60 | 1 |  | x | x |
| *Atf3*^(-/-)^ | CpG | 120 | 1 |  | x | x |
| *Atf3*^(-/-)^ | CpG | 240 | 1 |  | x | x |
| *Atf3*^(-/-)^ | LPS | 20 | 1 |  | x | x |
| *Atf3*^(-/-)^ | LPS | 60 | 3 |  | x | x |
| *Atf3*^(-/-)^ | LPS | 120 | 3 |  | x | x |
| *Atf3*^(-/-)^ | LPS | 240 | 3 |  | x | x |
| *Atf3*^(-/-)^ | LPS | 360 | 3 |  | x | x |
| *Atf3*^(-/-)^ | LPS | 480 | 3 |  | x | x |
| *Atf3*^(-/-)^ | Pam_2_CSK_4_ | 60 | 1 |  | x | x |
| *Atf3*^(-/-)^ | Pam_2_CSK_4_ | 120 | 1 |  | x | x |
| *Atf3*^(-/-)^ | Pam_2_CSK_4_ | 240 | 1 |  | x | x |
| *Atf3*^(-/-)^ | poly I:C | 60 | 1 |  | x | x |
| *Atf3*^(-/-)^ | poly I:C | 120 | 1 |  | x | x |
| *Atf3*^(-/-)^ | poly I:C | 240 | 1 |  | x | x |
| *Crem*^(-/-)^ | unstimulated | 0 | 3 |  | x | x |
| *Crem*^(-/-)^ | LPS | 60 | 3 |  | x | x |
| *Crem*^(-/-)^ | LPS | 120 | 3 |  | x | x |
| *Crem*^(-/-)^ | LPS | 240 | 2 |  | x | x |
| *Crem*^(-/-)^ | LPS | 360 | 2 |  | x | x |
| *Crem*^(-/-)^ | LPS | 480 | 2 |  | x | x |
| *Crem*^(-/-)^ | poly I:C | 120 | 2 |  | x | x |
| *Crem*^(-/-)^ | poly I:C | 240 | 2 |  | x | x |
| *Crem*^(-/-)^ | poly I:C | 360 | 2 |  | x | x |
| *Crem*^(-/-)^ | poly I:C | 480 | 2 |  | x | x |
| *Myd88*^(-/-)^ | unstimulated | 0 | 2 |  | x |  |
| *Myd88*^(-/-)^ | LPS | 20 | 2 |  | x |  |
| *Myd88*^(-/-)^ | LPS | 60 | 2 |  | x |  |
| *Myd88*^(-/-)^ | LPS | 120 | 2 |  | x |  |
| *Myd88*^(-/-)^ | Pam_3_CSK_4_ | 20 | 1 |  | x |  |
| *Myd88*^(-/-)^ | Pam_3_CSK_4_ | 60 | 1 |  | x |  |
| *Myd88*^(-/-)^ | Pam_3_CSK_4_ | 120 | 1 |  | x |  |
| *Myd88*^(-/-)^ | poly I:C | 20 | 2 |  | x |  |
| *Myd88*^(-/-)^ | poly I:C | 60 | 2 |  | x |  |
| *Myd88*^(-/-)^ | poly I:C | 120 | 2 |  | x |  |
| *Ticam1*^(Lps2/Lps2)^ | unstimulated | 0 | 1 |  | x |  |
| *Ticam1*^(Lps2/Lps2)^ | LPS | 60 | 2 |  | x |  |
| *Ticam1*^(Lps2/Lps2)^ | LPS | 120 | 2 |  | x |  |
| *Ticam1*^(Lps2/Lps2)^ | LPS / Pam_2_CSK_4_ | 60 | 1 |  | x |  |
| *Ticam1*^(Lps2/Lps2)^ | LPS / Pam_2_CSK_4_ | 120 | 1 |  | x |  |
| Wild-type | unstimulated | 0 | 13 | x | x |  |
| Wild-type | CpG | 20 | 2 | x | x |  |
| Wild-type | CpG | 40 | 2 | x | x |  |
| Wild-type | CpG | 60 | 2 | x | x |  |
| Wild-type | CpG | 80 | 2 | x | x |  |
| Wild-type | CpG | 120 | 2 | x | x |  |
| Wild-type | LPS | 20 | 3 | x | x | x |
| Wild-type | LPS | 40 | 3 | x | x | x |
| Wild-type | LPS | 60 | 3 | x | x | x |
| Wild-type | LPS | 80 | 3 | x | x | x |
| Wild-type | LPS | 120 | 3 | x | x | x |
| Wild-type | LPS | 240 | 3 | x | x | x |
| Wild-type | LPS | 360 | 2 | x | x | x |
| Wild-type | LPS | 480 | 3 | x | x | x |
| Wild-type | LPS | 720 | 2 | x | x | x |
| Wild-type | LPS | 1080 | 2 | x | x | x |
| Wild-type | LPS | 1440 | 4 | x | x | x |
| Wild-type | LPS | 2880 | 1 | x | x | x |
| Wild-type | Pam_2_CSK_4_ | 20 | 3 | x | x | x |
| Wild-type | Pam_2_CSK_4_ | 40 | 3 | x | x | x |
| Wild-type | Pam_2_CSK_4_ | 60 | 3 | x | x | x |
| Wild-type | Pam_2_CSK_4_ | 80 | 3 | x | x | x |
| Wild-type | Pam_2_CSK_4_ | 120 | 3 | x | x | x |
| Wild-type | Pam_2_CSK_4_ | 2880 | 1 | x | x | x |
| Wild-type | Pam_3_CSK_4_ | 20 | 3 | x | x | x |
| Wild-type | Pam_3_CSK_4_ | 40 | 3 | x | x | x |
| Wild-type | Pam_3_CSK_4_ | 60 | 3 | x | x | x |
| Wild-type | Pam_3_CSK_4_ | 80 | 3 | x | x | x |
| Wild-type | Pam_3_CSK_4_ | 120 | 3 | x | x | x |
| Wild-type | Pam_3_CSK_4_ | 240 | 2 | x | x | x |
| Wild-type | Pam_3_CSK_4_ | 360 | 2 | x | x | x |
| Wild-type | Pam_3_CSK_4_ | 480 | 4 | x | x | x |
| Wild-type | Pam_3_CSK_4_ | 720 | 2 | x | x | x |
| Wild-type | Pam_3_CSK_4_ | 2880 | 1 | x | x | x |
| Wild-type | Pam_3_CSK_4_ / poly I:C | 20 | 2 | x | x | x |
| Wild-type | Pam_3_CSK_4_ / poly I:C | 60 | 2 | x | x | x |
| Wild-type | Pam_3_CSK_4_ / poly I:C | 120 | 2 | x | x | x |
| Wild-type | poly I:C | 20 | 3 | x | x | x |
| Wild-type | poly I:C | 40 | 3 | x | x | x |
| Wild-type | poly I:C | 60 | 3 | x | x | x |
| Wild-type | poly I:C | 80 | 3 | x | x | x |
| Wild-type | poly I:C | 120 | 3 | x | x | x |
| Wild-type | poly I:C | 240 | 2 | x | x | x |
| Wild-type | poly I:C | 480 | 2 | x | x | x |
| Wild-type | poly I:C | 720 | 2 | x | x | x |
| Wild-type | poly I:C | 2880 | 1 | x | x | x |
| Wild-type | R848 | 20 | 2 | x | x | x |
| Wild-type | R848 | 40 | 2 | x | x | x |
| Wild-type | R848 | 60 | 2 | x | x | x |
| Wild-type | R848 | 80 | 2 | x | x | x |
| Wild-type | R848 | 120 | 2 | x | x | x |
| Wild-type | R848 | 240 | 2 | x | x | x |
| Wild-type | R848 | 480 | 2 | x | x | x |
| Wild-type | R848 | 720 | 2 | x | x | x |
