# Supplementary material for: Uncovering a Macrophage Transcriptional Program by Integrating Evidence from Motif Scanning and Expression Dynamics
Source: PLoS Comput Biol. 2008 Mar 21;4(3):e1000021. doi: 10.1371/journal.pcbi.1000021 (PMC2265556; doi:10.1371/journal.pcbi.1000021)
Supplement: Table S5 — Differentially expressed transcription factor genes considered as possible regulators of co-expressed gene clusters in this study. Column 1 contains gene symbol. Column contains the NCBI Entrez GeneID for the gene. Column 3 contains the representative Affymetrix probeset selected for the gene. Column 4 contains the co-expressed gene cluster of which the transcription factor is a member. Column 5 contains the TRANSFAC position-weight matrices that are associated with the transcription factor (or TF component) coded for by this gene (see Materials and Methods, Selection of Transcription Factors). The “V$” prefixes on TRANSFAC matrices are not shown. (0.13 MB DOC) [file pcbi.1000021.s023.doc]

| **Symbol** | **GeneID** | **Probeset** | **Cluster** | **TRANSFAC matrices** |
| --- | --- | --- | --- | --- |
| *Ahr* | 11622 | 1422631_at | 11 | AHRARNT_01, AHRARNT_02, AHRHIF_Q6, AHR_01, AHR_Q5 |
| *Atf1* | 11908 | 1417296_at | 14 | ATF1_Q6, CREBATF_Q6, CREB_Q3 |
| *Atf3* | 11910 | 1449363_at | 25 | ATF3_Q6, CREBATF_Q6 |
| *Cbfb* | 12400 | 1460716_a_at | 4 | AML_Q6, PEBP_Q6 |
| *Cebpa* | 12606 | 1418982_at | 19 | CEBPA_01, CEBP_01, CEBP_C, CEBP_Q2, CEBP_Q2_01, CEBP_Q3, CHOP_01 |
| *Cebpb* | 12608 | 1418901_at | 15 | CEBPB_01, CEBPB_02, CEBP_Q2_01, CEBP_Q3 |
| *Cebpd* | 12609 | 1423233_at | 17 | CEBPDELTA_Q6, CEBP_Q2_01, CEBP_Q3 |
| *Cebpg* | 12611 | 1451639_at | 22 | CEBPGAMMA_Q6, CEBP_Q2_01, CEBP_Q3 |
| *Ddit3* | 13198 | 1417516_at | 22 | CHOP_01 |
| *E2f1* | 13555 | 1431875_a_at | 2 | E2F1_Q4, E2F1_Q4_01, E2F1_Q6, E2F1_Q6_01, E2F_01, E2F_02, E2F_03, E2F_Q2, E2F_Q3_01, E2F_Q4_01, E2F_Q6_01 |
| *E2f6* | 50496 | 1448835_at | 10 | E2F1_Q4_01, E2F_03, E2F_Q2, E2F_Q3_01, E2F_Q4_01, E2F_Q6_01 |
| *E2f7* | 52679 | 1437187_at | 3 | E2F1_Q4_01, E2F_03, E2F_Q2, E2F_Q3_01, E2F_Q4_01, E2F_Q6_01 |
| *Egr1* | 13653 | 1417065_at | 27 | EGR1_01, EGR_Q6, KROX_Q6 |
| *Egr2* | 13654 | 1427683_at | 27 | EGR2_01, KROX_Q6 |
| *Egr3* | 13655 | 1436329_at | 27 | KROX_Q6 |
| *Elf1* | 13709 | 1417540_at | 29 | ELF1_Q6, ETS_Q4, ETS_Q6 |
| *Elk3* | 13713 | 1448797_at | 10 | ETS_Q6 |
| *Esrra* | 26379 | 1442864_at | 11 | ERR1_Q2 |
| *Fli1* | 14247 | 1433512_at | 14 | ETS_Q4, ETS_Q6 |
| *Fos* | 14281 | 1423100_at | 27 | AP1FJ_Q2, AP1_01, AP1_C, AP1_Q2, AP1_Q2_01, AP1_Q4, AP1_Q4_01, AP1_Q6, AP1_Q6_01 |
| *Foxm1* | 14235 | 1448834_at | 3 | FOXM1_01 |
| *Foxo3a* | 56484 | 1434832_at | 14 | FOXO3_01 |
| *Foxp1* | 108655 | 1421141_a_at | 21 | FOXP1_01 |
| *Gabpa* | 14390 | 1450664_at | 20 | ETS_Q6, GABP_B |
| *Hif1a* | 15251 | 1457231_at | 17 | AHRHIF_Q6, HIF1_Q3, HIF1_Q5 |
| *Hmga2* | 15364 | 1422851_at | 7 | HMGIY_Q3, HMGIY_Q6 |
| *Hoxa4* | 15401 | 1441070_at | 22 | HOXA4_Q2 |
| *Ikzf1* | 22778 | 1436312_at | 21 | IK1_01, IK2_01, IK3_01, LYF1_01 |
| *Irf1* | 16362 | 1448436_a_at | 25 | IRF1_01, IRF1_Q6, IRF_Q6, IRF_Q6_01 |
| *Irf2* | 16363 | 1447527_at | 13 | IRF2_01, IRF_Q6, IRF_Q6_01 |
| *Irf3* | 54131 | 1416898_a_at | 12 | IRF_Q6, IRF_Q6_01 |
| *Irf5* | 27056 | 1460231_at | 6 | IRF_Q6, IRF_Q6_01 |
| *Irf7* | 54123 | 1417244_a_at | 6 | IRF_Q6, IRF_Q6_01 |
| *Isgf3g* | 16391 | 1421322_a_at | 6 | IRF_Q6_01 |
| *Jun* | 16476 | 1417409_at | 20 | AP1FJ_Q2, AP1_01, AP1_C, AP1_Q2, AP1_Q2_01, AP1_Q4, AP1_Q4_01, AP1_Q6, AP1_Q6_01, CREBP1CJUN_01 |
| *Junb* | 16477 | 1415899_at | 28 | AP1_01, AP1_C, AP1_Q2_01, AP1_Q4_01, AP1_Q6_01 |
| *Lmo2* | 16909 | 1454086_a_at | 18 | LMO2COM_01, LMO2COM_02 |
| *Maf* | 17132 | 1444073_at | 12 | MAF_Q6_01 |
| *Mafb* | 16658 | 1451716_at | 19 | MAF_Q6_01 |
| *Maff* | 17133 | 1418936_at | 27 | MAF_Q6_01 |
| *Mafg* | 17134 | 1448916_at | 23 | MAF_Q6_01 |
| *Mef2a* | 17258 | 1427185_at | 2 | MEF2_01, MEF2_02, MEF2_03, MEF2_04, MEF2_Q6_01, MMEF2_Q6 |
| *Mef2c* | 17260 | 1424852_at | 16 | MEF2_Q6_01 |
| *Mitf* | 17342 | 1455214_at | 13 | EBOX_Q6_01 |
| *Mtf1* | 17764 | 1428979_at | 17 | MTF1_Q4 |
| *Mxd1* | 17119 | 1455104_at | 24 | EBOX_Q6_01 |
| *Mxd4* | 17122 | 1434378_a_at | 14 | EBOX_Q6_01 |
| *Myc* | 17869 | 1424942_a_at | 20 | EBOX_Q6_01, MYCMAX_01, MYCMAX_02, MYCMAX_03, MYCMAX_B, MYC_Q2 |
| *Nfatc1* | 18018 | 1428479_at | 14 | NFAT_Q4_01, NFAT_Q6 |
| *Nfatc2* | 18019 | 1439205_at | 14 | NFAT_Q4_01, NFAT_Q6 |
| *Nfatc3* | 18021 | 1452497_a_at | 23 | NFAT_Q6 |
| *Nfe2l1* | 18023 | 1416331_a_at | 22 | MAF_Q6_01 |
| *Nfe2l2* | 18024 | 1457117_at | 27 | MAF_Q6_01, NRF2_Q4 |
| *Nfic* | 18029 | 1435527_at | 19 | NF1_Q6 |
| *Nfkb1* | 18033 | 1427705_a_at | 15 | NFKAPPAB_01, NFKB_C, NFKB_Q6, NFKB_Q6_01 |
| *Nfyc* | 18046 | 1448963_at | 4 | NFY_Q6, NFY_Q6_01 |
| *Nr1h3* | 22259 | 1450444_a_at | 9 | DR4_Q2, LXR_DR4_Q3, LXR_Q3, PXR_Q2 |
| *Nr3c1* | 14815 | 1421867_at | 6 | GRE_C, GR_Q6, GR_Q6_01, PR_Q2 |
| *Pou2f2* | 18987 | 1427725_a_at | 9 | OCT_C, OCT_Q6 |
| *Pou3f1* | 18991 | 1422068_at | 8 | OCT_Q6, TST1_01 |
| *Pparg* | 19016 | 1420715_a_at | 18 | DR1_Q3, PPARG_01, PPARG_02, PPARG_03, PPAR_DR1_Q2 |
| *Rel* | 19696 | 1420710_at | 25 | CREL_01 |
| *Rest* | 19712 | 1425565_at | 31 | NRSE_B |
| *Rfx5* | 53970 | 1423103_at | 8 | RFX_Q6 |
| *Rfxap* | 170767 | 1418859_at | 32 | RFX_Q6 |
| *Rxra* | 20181 | 1454773_at | 14 | DR1_Q3, DR3_Q4, DR4_Q2, FXR_IR1_Q6, FXR_Q3, LXR_DR4_Q3, LXR_Q3, PPARA_02, PPAR_DR1_Q2, PXR_Q2, T3R_Q6 |
| *Sfpi1* | 20375 | 1418747_at | 17 | ETS_Q6, PU1_Q6 |
| *Smad1* | 17125 | 1448208_at | 14 | SMAD_Q6, SMAD_Q6_01 |
| *Sp1* | 20683 | 1454852_at | 5 | SP1_Q2_01, SP1_Q6, SP1_Q6_01 |
| *Srebf1* | 20787 | 1426690_a_at | 5 | SREBP_Q3 |
| *Stat1* | 20846 | 1420915_at | 6 | STAT1_01, STAT1_02, STAT1_03, STAT_01, STAT_Q6 |
| *Stat3* | 20848 | 1426587_a_at | 6 | STAT3_01, STAT3_02, STAT_01, STAT_Q6 |
| *Stat4* | 20849 | 1448713_at | 22 | STAT4_01, STAT_01, STAT_Q6 |
| *Stat5a* | 20850 | 1421469_a_at | 21 | STAT5A_01, STAT5A_02, STAT5A_03, STAT5A_04, STAT_01, STAT_Q6 |
| *Tcf12* | 21406 | 1421908_a_at | 5 | EBOX_Q6_01 |
| *Tcfe2a* | 21423 | 1426297_at | 2 | E12_Q6, E2A_Q2, E2A_Q6, EBOX_Q6_01, MYOD_Q6_01 |
| *Tgif1* | 21815 | 1422286_a_at | 27 | TGIF_01 |
| *Trp53* | 22059 | 1427739_a_at | 2 | P53_01, P53_02, P53_DECAMER_Q2 |
| *Usf2* | 22282 | 1460228_at | 16 | EBOX_Q6_01, USF2_Q6, USF_Q6_01 |
| *Zfp161* | 22666 | 1420865_at | 19 | ZF5_01, ZF5_B |
