# Supplementary material for: Uncovering a Macrophage Transcriptional Program by Integrating Evidence from Motif Scanning and Expression Dynamics
Source: PLoS Comput Biol. 2008 Mar 21;4(3):e1000021. doi: 10.1371/journal.pcbi.1000021 (PMC2265556; doi:10.1371/journal.pcbi.1000021)
Supplement: Table S6 — Summary of co-expressed gene clusters. Column 1 indicates the cluster name. Clusters were numbered in order of decreasing size. Column 2 indicates the number of genes in the cluster. Column 3 is a heat-map representation of the within-cluster median of the normalized differential expression intensity (SDR, see Equation 1), over time, in wild-type macrophages stimulated with LPS. The color red indicates upregulation relative to wild-type unstimulated macrophages, and green indicates downregulation (see color bar in Figure S2). Column 4 indicates the cluster response time under LPS stimulation, defined as the time scale (in minutes) for the log2 fold change to reach 25% of its extremal value (see Materials and Methods, Expression Clustering); the time scale uncertainty is ± 5 min. Column 5 lists the known (excluding those solely inferred from electronic annotation, i.e., “IEA” evidence code) transcription factor genes that are members of the cluster (these are not the inferred transcriptional regulators of the cluster). Column 6 lists the known cytokines and chemokines that are members of the indicated cluster. (0.13 MB DOC) [file pcbi.1000021.s024.doc]

| **Cluster** | **Size** | **LPS resp** | **Ind time** | **Transcription factor genes that are members of the cluster** | **Cytokine & chemokine genes that are members of the cluster** |
| --- | --- | --- | --- | --- | --- |
| C1 | 113 | 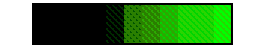 | 194 | *Ezh2, Hmgb2, Hmgn1, Hmgn2, Phf19* |  |
| C2 | 108 | 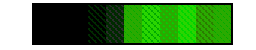 | 154 | *Bin1, Etv5, Gtf3a, Mef2a, Taf9* |  |
| C3 | 106 | 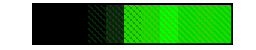 | 148 | *Brca2, E2f7, Foxm1, Tcf19, Tfdp1, Tfdp2, Tieg3, Uhrf1* |  |
| C4 | 103 | 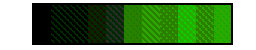 | 172 | *Aatf, Cbfb, Cebpz, Crsp2, Lass5, Mybbp1a, Pa2g4* | *Scye1* |
| C5 | 103 | 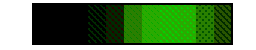 | 175 | *Gtf2ird1, Lrrfip1, Tcf12, Yaf2* |  |
| C6 | 101 | 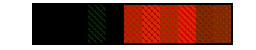 | 168 | *Etv6, Ncoa3, Nmi, Nr3c1, Snw1, Stat1* | *Ccl8, Cxcl11, Cxcl9* |
| C7 | 82 | 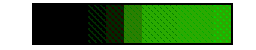 | 177 | *Dek, Hmga2, Rbl1* |  |
| C8 | 82 | 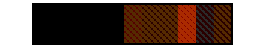 | 230 | *Mecp2, Mycl1, Pou3f1, Rfx5* | *ORF9, Tnfsf4* |
| C9 | 80 | 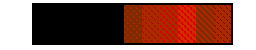 | 187 | *Aff1, Arid5b, Lmo4, Nr1h3* |  |
| C10 | 78 | 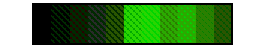 | 130 | *E2f6, Mxi1* |  |
| C11 | 70 | 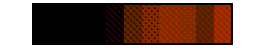 | 221 | *Ahr, Esrra, Jarid1b, Snapc1* | *Il1f9* |
| C12 | 65 | 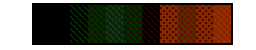 | 428 | *Edf1* | *Csf3, Il1f6* |
| C13 | 64 | 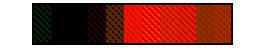 | 123 |  | *Ccl5, Fbs1* |
| C14 | 62 | 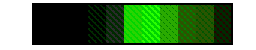 | 139 | *Fli1, Gtf2a2, Lyl1, Nfatc1, Nfatc2, Zfp36l1* |  |
| C15 | 61 | 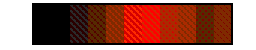 | 75 | *Bcl3, Hivep2, Klf7, Nfil3, Nfkb1, Nfkb2, Plagl2* | *Il12b, Il1b, Il6* |
| C16 | 61 | 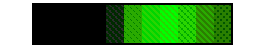 | 159 | *Cnot6l, Mef2c, Smarca2, Thrap3* |  |
| C17 | 58 | 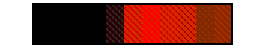 | 147 | *Batf, Cebpd, Hif1a, Klf3, Lztfl1, Pou2f2, Rnf24* | *Ebi3* |
| C18 | 57 | 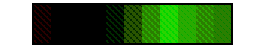 | 234 | *Atf6, Lmo2* | *Fam3c* |
| C19 | 57 | 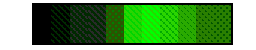 | 96 | *Mafb, Zfp36l2* |  |
| C20 | 55 | 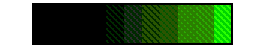 | 428 | *Jun, Nrip1* |  |
| C21 | 45 | 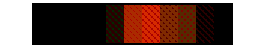 | 147 |  | *Ccl22, Ccl9, Il1a* |
| C22 | 44 | 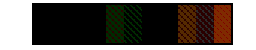 | 698 | *Atf7ip, Ddit3* | *Vegfa* |
| C23 | 42 | 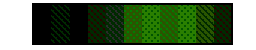 | 209 |  |  |
| C24 | 40 | 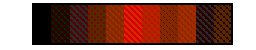 | 69 | *Arid5a, Creb5, Etv3, Hivep1, Mxd1* | *Csf1, Ifnb1* |
| C25 | 37 | 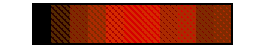 | 43 | *Irf1, Prdm1, Rel* | *Cxcl10, Il10* |
| C26 | 35 | 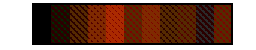 | 60 | *Bcor, Jundm2, Prdm2* | *Csf2, Gm1960* |
| C27 | 30 | 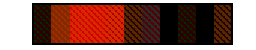 | 27 | *Egr1, Egr2, Egr3, Maff* | *Tnfsf9* |
| C28 | 28 | 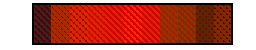 | 22 | *Ccrn4l, Junb, Klf6* | *Ccl3, Ccl4, Cxcl1, Cxcl2, Tnf* |
| C29 | 27 | 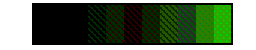 | 963 |  |  |
| C30 | 26 | 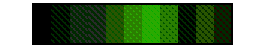 | 104 | *Hhex* |  |
| C31 | 22 | 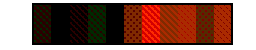 | 202 | *Bbx, Rest* |  |
| C32 | 18 | 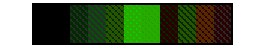 | 124 |  |  |
