# Supplementary material for: Uncovering a Macrophage Transcriptional Program by Integrating Evidence from Motif Scanning and Expression Dynamics
Source: PLoS Comput Biol. 2008 Mar 21;4(3):e1000021. doi: 10.1371/journal.pcbi.1000021 (PMC2265556; doi:10.1371/journal.pcbi.1000021)
Supplement: Table S7 — The timing of induction of core response clusters C27 and C28 is adapter molecule-dependent. Column 1 indicates the stimulus. Column 2 indicates the microarray conditions compared, for example, fold-change (stimulated relative to unstimulated) in Myd88 (−/−) macrophages vs. the fold-change in wild-type. Column 3 indicates the time post-stimulation. Columns 4 and 5 are the within-cluster medians of the log2 of the ratios for the condition comparison indicated in column 2, for the clusters C27 and C28, respectively. The data indicate that the early response of these clusters is largely dependent on the MyD88 signaling pathway, and that the later response (2 hours) is more strongly dependent on the TRIF signaling pathway. (0.03 MB DOC) [file pcbi.1000021.s025.doc]

| **Stimulus** | **Conditions compared** | **Time (min)** | **C27** | **C28** |
| --- | --- | --- | --- | --- |
| LPS | *Myd88*^(-/-)^ vs. wild-type | 60 | -1.02 | -0.87 |
| LPS | *Myd88*^(-/-)^ vs. wild-type | 120 | -0.21 | -0.41 |
| LPS | *Ticam*^(Lps2/Lps2)^ vs. wild-type | 60 | -0.19 | -0.53 |
| LPS | *Ticam*^(Lps2/Lps2)^ vs. wild-type | 120 | -1.07 | -0.99 |
