# Supplementary material for: Uncovering a Macrophage Transcriptional Program by Integrating Evidence from Motif Scanning and Expression Dynamics
Source: PLoS Comput Biol. 2008 Mar 21;4(3):e1000021. doi: 10.1371/journal.pcbi.1000021 (PMC2265556; doi:10.1371/journal.pcbi.1000021)
Supplement: Table S9 — Time-course macrophage stimulation microarray experiments used for time-lagged correlation analysis. Only time-course expression studies with a sufficient number of time points to admit time-lagged correlation analysis are shown (see Materials and Methods, Time-lagged Correlation). Column 1 indicates the genotype from which macrophages were derived. Column 2 indicates the stimulus used. Column 3 indicates the times post-stimulation, at which gene expression was measured. (0.04 MB DOC) [file pcbi.1000021.s027.doc]

| Genotype | Stimulus | Time points (min.) |
| --- | --- | --- |
| Wild-type | LPS | 0, 20, 40, 60, 80, 120, 240, 360, 480, 720, 1080, 1440, 2880 |
| Wild-type | poly I:C | 0, 20, 40, 60, 80, 120, 240, 480, 720, 2880 |
| Wild-type | Pam_2_CSK_4_ | 0, 20, 40, 60, 80, 120, 2880 |
| Wild-type | Pam_3_CSK_4_ | 0, 20, 40, 60, 80, 120, 240, 360, 480, 720, 2880 |
| Wild-type | R848 | 0, 20, 40, 60, 80, 120, 240, 480, 720 |
| *Atf3*^(-/-)^ | CpG | 0, 60, 120, 240 |
| *Atf3*^(-/-)^ | LPS | 0, 20, 60, 120, 240, 360, 480 |
| *Atf3*^(-/-)^ | Pam_2_CSK_4_ | 0, 60, 120, 240 |
| *Atf3*^(-/-)^ | poly I:C | 0, 60, 120, 240 |
| *Crem*^(-/-)^ | LPS | 0, 60, 120, 240, 360, 480 |
| *Crem*^(-/-)^ | poly I:C | 0, 120, 240, 360, 480 |
