# Supplementary material for: Uncovering a Macrophage Transcriptional Program by Integrating Evidence from Motif Scanning and Expression Dynamics
Source: PLoS Comput Biol. 2008 Mar 21;4(3):e1000021. doi: 10.1371/journal.pcbi.1000021 (PMC2265556; doi:10.1371/journal.pcbi.1000021)
Supplement: Table S15 — List of key materials and reagents. Column 1 indicates the type of material (mouse strain or stimulus reagent). Column 2 indicates the specific strain or reagent. For mutant mouse strains, the Mouse Genome Informatics accession number of the allele is provided. Column 3 indicates the source laboratory from which the mouse strain or reagent was obtained. (0.05 MB DOC) [file pcbi.1000021.s033.doc]

| **Type** | **Identifier** | **Source** |
| --- | --- | --- |
| mouse strain | *Atf3*^(-/-)^ (MGI:3531501) | Dr. Tsonwin Hai, Ohio State University, Columbus, OH, USA |
| mouse strain | *Crem*^(-/-)^ (MGI:2181391) | Dr. Günther Schütz, German Cancer Research Center, Heidelberg, Germany; (with Dr. Julie Blendy) |
| mouse strain | *Myd88*^(-/-)^ (MGI:2385681) | Dr. Shizuo Akira, Osaka University, Osaka, Japan |
| mouse strain | *Ticam1*^(Lps2/Lps2)^ (MGI:2679794) | Dr. Bruce Beutler, Scripps Research Institute, La Jolla, CA, USA |
| mouse strain | C57BL/6 (wild-type) | Jackson Laboratories, Bar Harbor, ME, USA |
| stimulus | CpG | Invivogen, San Diego, CA, USA |
| stimulus | LPS (from  *S. minnesota*) | List Biological Labs, Campbell, CA, USA |
| stimulus | Pam_2_CSK_4_ | EMC microcollections GmbH, Tuebingen, Germany |
| stimulus | Pam_3_CSK_4_ | EMC microcollections GmbH, Tuebingen, Germany |
| stimulus | poly I:C | Amersham Biosciences, Piscataway, NJ, USA |
| stimulus | R848 | GL Synthesis, Worcester, MA, USA |
| antibody | p50/p105 polyclonal antibody | eBioscience, San Diego, CA, USA |
| antibody | murine IRF1, sc-640x | Santa Cruz Biotechnology, Santa Cruz, CA, USA |
| qPCR primer / probe | *Tgif1*, Mm00493650_m1 | Applied Biosystems, Foster City, CA, USA |
| qPCR primer / probe | *Eef1a1,*  (custom synthesized) | Integrated DNA Technologies, Coralville, IA, USA  Forward: GCA AAA ACG ACC CAC CAA TG  Reverse: GGC CTG GAT GGT TCA GGA TA  Probe: 5'FAM CAC CTG AGC AGT GAA GCC AG 3'TAMRA |
