# Supplementary material for: Uncovering a Macrophage Transcriptional Program by Integrating Evidence from Motif Scanning and Expression Dynamics
Source: PLoS Comput Biol. 2008 Mar 21;4(3):e1000021. doi: 10.1371/journal.pcbi.1000021 (PMC2265556; doi:10.1371/journal.pcbi.1000021)
Supplement: Table S16 — Summary of probeset selection criteria. Each row describes a set of data selection criteria, for a specific purpose. For a detailed explanation of each set of criteria, see Materials and Methods, Probeset Selection. Column 1 states the purpose of the set of selection criteria. Column 2 indicates the minimum log2 absolute probeset intensity that must have been recorded in at least one experiment, for the gene to be included in the selection described in Column 1. Column 3 indicates the false discovery rate used to determine the P value cutoffs for each of the seven time-course experiments used for differential expression testing (see Materials and Methods, Differential Expression Testing); “n/a” means that no differential expression test was applied, for genes in the indicated row. Column 4 gives the number of probesets resultant from the indicated selection criteria. (0.03 MB DOC) [file pcbi.1000021.s034.doc]

| **Purpose** | **log_2_ cutoff** | | FDR **cutoff** | **Number of probesets** |
| --- | --- | --- | --- | --- |
| Differentially expressed genes | 7 | 10^-4^ | | 1,960 |
| Differentially expressed TF genes (with known motifs) | 7 | | 0.05 | 80 |
| All genes expressed at log_2_ ≥ 7 | 7 | | n/a | 8,788 |
| All unique genes represented by “_at” or “_a_at” probesets on the Affymetrix GeneChip | 0 | | n/a | 20,905 |
