# Supplementary material for: Correction: Uncovering a Macrophage Transcriptional Program by Integrating Evidence from Motif Scanning and Expression Dynamics
Source: PLoS Comput Biol. 2008 Mar 25;4(3):10.1371/annotation/1c55be5f-ecd7-49be-91c1-91881be60297. doi: 10.1371/annotation/1c55be5f-ecd7-49be-91c1-91881be60297 (PMC2638128; doi:10.1371/annotation/1c55be5f-ecd7-49be-91c1-91881be60297)
Supplement: Supplementary file 1 [file pcbi.1c55be5f-ecd7-49be-91c1-91881be60297.s001.doc]

| Criteria | Number of genes |
| --- | --- |
| Total representative genes (see **Materials and Methods – Probeset Selection**): | 20,905 |
| Have at least one GO annotation: | 12,515 |
| Have at least one GO process annotation: | 8,058 |
| Have at least one GO component annotation: | 7,797 |
| Have at least one GO function annotation: | 10,170 |
